# Inferences of p-values, statistically significant results, and confidence intervals – A systematic review of empirical studies

**DOI:** 10.1371/journal.pone.0362071

**Published:** 2026-11-11

**Authors:** Per Lytsy, Per Johansson, Ronnie Pingel, Jenny Berg, Charlotta Ryk

**Affiliations:** 1 Department of Public Health and Caring Sciences, University of Uppsala, Uppsala, Sweden; 2 Department of Clinical Neuroscience, Karolinska Institutet, Stockholm, Sweden; 3 Department of Statistics, University of Uppsala, Uppsala, Sweden; 4 YMSC, Tsinghua University, Tsinghua, China; 5 Department of Learning, Informatics, Management and Ethics (LIME), Karolinska Institutet, Stockholm, Sweden; 6 Independent researcher, Stockholm, Sweden; University of Bologna, ITALY

## Abstract

This systematic review aimed to investigate how researchers, educators, students, and professionals understand and interpret statistical inference. Following PRISMA guidelines, four databases were searched through 8 August 2025 with no time restrictions for empirical studies on interpretations of p-values, statistical significance, and confidence intervals. Fifty-five studies published between 1963 and 2024 were included, encompassing over 15,000 participants from diverse regions, including Europe, North America, Australia, China, South America, and the Middle East. Studies primarily used written or online cross-sectional surveys, employing various single questions or questionnaires to assess understanding and inferential reasoning about statistical results. Participants ranged from students and PhD candidates to researchers, clinicians, statisticians, and methodology instructors with substantial statistical education and experience. Results showed widespread and persistent misunderstandings across all groups. Common errors about p-values and null hypothesis testing included the inverse probability fallacy, misinterpreting p-values as probabilities of hypotheses, the replication fallacy, and conflating statistical significance with clinical or practical relevance. Confidence intervals were often misinterpreted as probabilistic ranges for true parameters or as predictors of future outcomes. Presenting effect estimates with confidence intervals rather than p-values reduced some misinterpretations, but high error rates persisted. These findings highlight the need for improved statistical education, clearer communication of core concepts, and careful presentation of results in scientific literature. Misinterpretations of statistical inference are pervasive, with implications for research reproducibility, scientific literacy, and evidence-based decision-making.

## Introduction

Empirical research draws conclusions from observed data. To answer research questions using quantitative data, researchers use various statistical frameworks and procedures to make inferences—that is, to draw conclusions about a sample and, more commonly, to generalize beyond the study sample.

This systematic review examines empirical studies that investigate inferences drawn from the most common outputs of frequentist statistics, specifically results from significance testing, hypothesis testing, and confidence interval estimation. These methods, introduced in the 1920s and 1930s, have since become foundational in statistical analysis, though they remain subjects of considerable debate. An in-depth review of these methods and their controversies lies beyond the scope of this article, and several perspective and summary papers on the topic are already available [1–9]. However, a basic understanding of these methods, their theoretical foundations, and historical origins is essential to contextualize the results of this systematic review.

The p-value, a key outcome of significance testing, builds on Karl Pearson’s earlier development of the chi-squared test but is commonly attributed to Ronald Fisher and the theory of inductive inference or inductive reasoning [10]. The p-value represents the probability of obtaining a test statistic as extreme as, or more extreme than, the one observed, given the null hypothesis is true.

Fisher focused on what could be learnt from the data when contrasted with a null hypothesis and he regarded the p-value as a tool for assessing the strength of evidence against a null hypothesis, not as a definitive yes-or-no test. Initially, Fisher proposed using a conventional level like 0.05 as a threshold to indicate whether results were worth further attention. Later, however, he suggested reporting the actual p-value after the experiment was conducted to determine whether the results were significant or not [10–12].

Fisher later extended the theory about statistical inferences with the randomization test, a non-parametric approach which uses the randomization process to justify inference [11]. In a randomized experiment, with treatment and control groups, the null hypothesis assumes that there is no effect for any unit in the experiment. The test is valid without any further assumptions [13–15], making it an example of *design-based inference*. This approach allows for evaluating whether it is likely that chance alone caused the observed effect—not in the population, but specifically within the sample (or experimental units).

In contrast to Fisher’s inductive approach to inference, Jerzy Neyman and Egon Pearson introduced what Neyman would later describe as inductive behavior. The goal of hypothesis testing, in their framework, was to establish a behavioral decision rule that, over repeated sampling from the same population, would control the long-run frequency of errors. Their theory introduced two competing hypotheses—the null hypothesis and the alternative hypothesis—and a rule for deciding between them based on a pre-specified rejection level. According to this approach, the null hypothesis is rejected if the p-value (or more broadly if the test statistic falls within the rejection area of an assumed distribution) is less than the predetermined threshold [16]. This pre-set rejection level is now known as the significance level, or the α-level. The significance level represents the risk of rejecting the null hypothesis, when it is actually true, i.e., conducting a Type I error. Thus, the significance level is defined as the maximum long-run proportion of Type I errors across repeated samples and does not apply to any single observed result.

Null hypothesis significance testing (NHST) is a procedure in which results are reported as “statistically significant” if the observed p-value falls below a preset significance level (α). NHST blends two distinct ideas: Fisher’s concept of the p-value as a measure of evidence against the null hypothesis, and Neyman and Pearson’s decision framework, which focuses on controlling long-run error rates. The fusion of approaches has led to widespread misinterpretation of p-values as direct error probabilities, creating the illusion that conclusions can be drawn with precisely known “error rates”, without considering any context beyond the experiment itself [5,17]. For Fisher, the p-value is an evidential measure in a particular case: a property of the observed data reflecting the strength of evidence against the null hypothesis. In contrast, the Neyman–Pearson’s α level is specified in advance and represents a property of the testing procedure under repeated sampling, assuming no systematic errors.

There are many published articles, including those cited above, discussing weaknesses, logical inconsistencies and common misunderstandings of the surrounding theories behind NHST, as well as the complications that arise from combining Fisher’s and Neyman–Pearson’s approaches into a single framework [7,8,12,18,19]. Common misunderstandings include misinterpreting the p-value as the probability that a null hypothesis is false, assuming that its complement represents the probability of successful replication, and the mistaken assumption that if one rejects a null hypothesis, one thereby affirms the theory that led to the test [6,19].

In 2016 the American Statistical Association issued a “Statement on Statistical Significance and P-Values” to clarify six principles for the correct use and interpretation of p-values [20]. The principles addressed misunderstandings, emphasizing that p-values do not represent the probability that the hypothesis is true, nor do they indicate how likely it is that the observed results occurred by chance. Furthermore, the principles clarified that a p-value or statistical significance does not quantify the effect size or the importance of a result, nor does it alone offer reliable evidence against (or for) a hypothesis.

Developed by a group of experts with diverse perspectives, the ASA’s principles aimed to provide a shared foundation for interpreting p-values. The statement was also accompanied by a supplement where many of these experts contributed individual articles discussing p-values and the implications of the statement.

Along with growing criticisms of the outputs of null hypothesis significance testing, many have suggested that results of statistical estimation should be presented as confidence intervals [21,22]. The concept of confidence interval estimation was introduced by Jerzy Neyman in 1934 and further outlined in 1937 [23,24]. Like hypothesis testing, confidence interval estimation is designed for a long-term perspective. In the absence of bias, a valid confidence interval procedure, when applied over repeated samplings, will contain the true parameter value with a frequency consistent with the specified confidence level, typically 95%. Neyman emphasized that using confidence intervals did not imply a phase of reasoning or inference about the truth of a parameter. Rather, it represented a possibility of an intentional choice to adjust our behavior based on the interval’s bounds – a concept he termed inductive behavior, positioning it as an alternative to Fisher’s inductive inference [25].

While introductory courses in statistics and the philosophy of science may cover these topics, several studies have demonstrated that the inferences drawn from research results presented as statistical outputs are often unclear. The aim of this study was to systematically search for and assess research, investigating how p-values, statistically significant findings, and confidence intervals are interpreted and inferred.

## Materials and methods

We conducted a systematic review following PRISMA reporting guidelines. Our search focused on empirical studies examining inferences, interpretation, and understanding of statistical concepts such as p-values, “statistical significance”, and confidence intervals. We included studies that investigated these inferential concepts among researchers, educators (at any academic level), students, and professionals (e.g., healthcare providers, technical experts, statisticians and educational specialists). Studies involving layperson perceptions and inferences were excluded. No restrictions were applied regarding the type of instrument or questions on inference, and all study designs that collected data through survey instruments, including written and web-based questionnaires, were considered. We excluded studies that analyzed trends in published literature regarding how authors described or interpreted statistical outputs, as well as those using qualitative methodologies. A research plan was developed at the outset of the project but was not prospectively registered. The plan was made publicly available on the Open Science Framework (OSF) in May 2025 (https://osf.io/qzxut).

### The literature search

An information specialist, i.e., a professional with expertise in literature searching, developed a search strategy in collaboration with the research group. The search strategy was based on blocks of key medical subject headings terms and truncated forms of free text words of statistical concepts such as “P-value”, “significance test” “statistics”, “confidence interval” and “interpretation”, “inferences” “fallacy” but also blocks terms related to understanding such as “deduction”, “plausibility”, “fallacy”, “misunderstandings”. The literature search was performed in four databases Cinahl, Pubmed, Psychinfo and Web of Science in December 2021, and was updated in April 2023, August 2024 and most recently on August 8, 2025. Potentially interesting references that were not identified in the search, but were either known by any of the authors, suggested by other researchers or identified by web searches, were also added for screening and considered for full text assessment. Furthermore, reference lists of included studies were screened for additional relevant literature. We included original peer-review published research articles but also grey literature such as original investigations of inferences published in books, theses or non-peer reviewed reports. The full search strategy is available in S1 Appendix.

### Screening of abstracts, assessment of relevance and risk of bias and tabulation

All references were independently screened by two authors based on title and abstract in the Covidence platform (www.covidence.com). If at least one in a pair considered a reference relevant it was discussed further. If deemed potentially relevant, i.e., there was reason to believe it involved an empirical investigation of inferences, then it was retrieved to be read in full length. Two authors then independently performed assessments of full-length articles to determine if the report included an empirical study of statistical inference, if so–it was included. Studies that were scrutinized in full length and excluded are listed in S1 Appendix, together with the main reason for exclusion. The main characteristics of the studies were extracted and tabulated by one author and independently quality-checked by another. A flowchart of the search results with number of included and excluded studies is presented in Fig 1.

**Fig 1:**
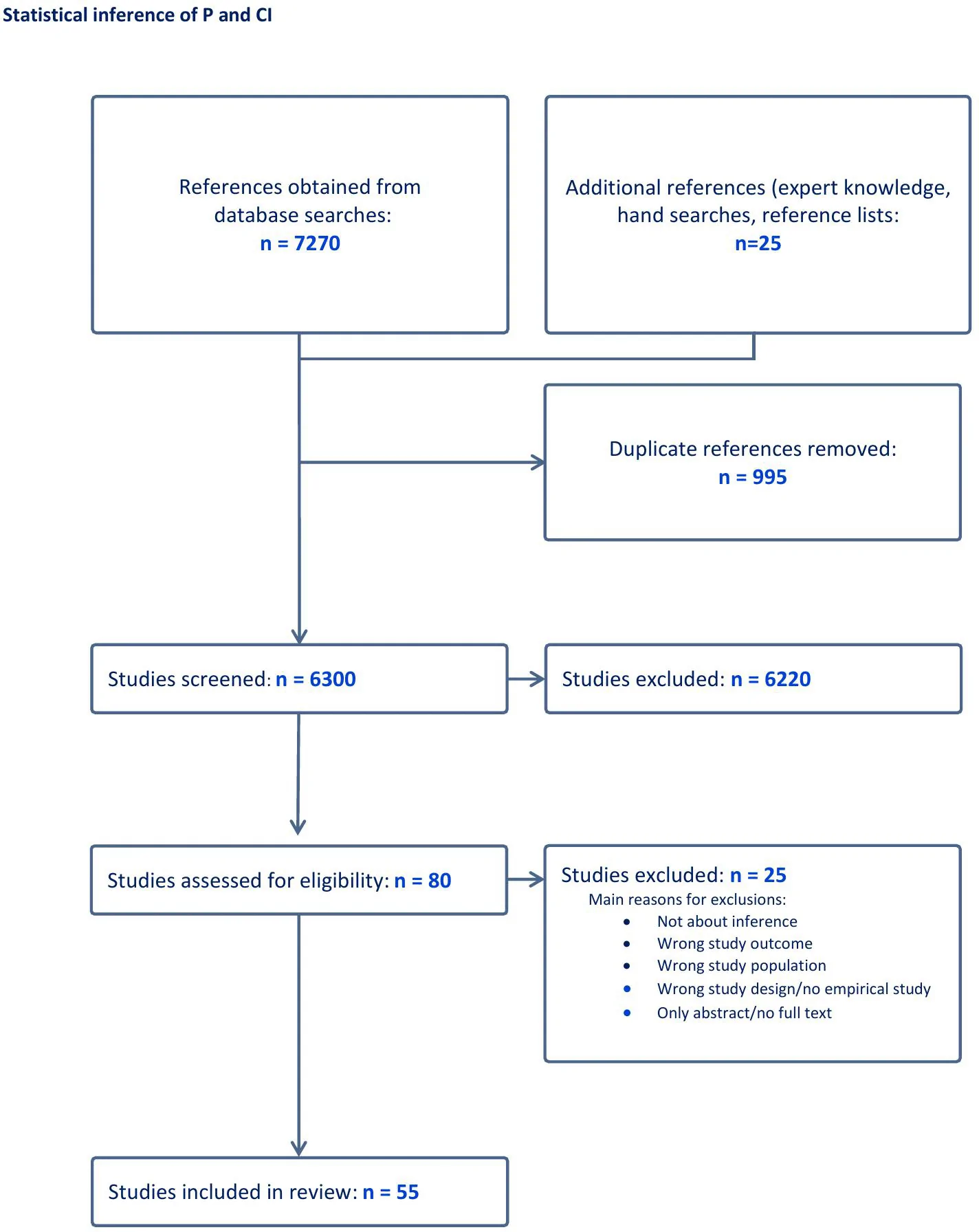
Flow diagram of study identification, screening, and inclusion.

### Assessment of risk of bias

Assessment of risk of bias serves mainly to examine if and how the study design and conduct may have given rise to misleading or distorted results. This is important when judging individual studies, when synthesizing results in meta-analyses, and when determining the certainty of synthesized evidence using frameworks such as Grading of Recommendations, Assessment, Development, and Evaluations (GRADE).

We considered it unlikely that results of our findings could be pooled, due to extensive heterogeneity, mainly in study populations and instruments and questions used. Nevertheless, we assessed all included studies for risk of bias using an adjusted version of the Risk of Bias Instrument for Cross-Sectional Surveys of Attitudes and Practices, developed by the CLARITY Group at McMaster University [26]. The instrument assesses biases across five domains, asking these questions: 1) is the source population representative of the population of interest?, 2) is the response rate adequate?, 3) is there little missing data?, 4) is the survey clinically sensible?, and 5) is there evidence of the reliability and validity of the survey instrument? Responses were assessed as: definitely/probably yes; definitely/probably no or no information/not applicable. The assessments were performed by two researchers, independently of each other, and conflicting assessments in domains were solved by consensus. We did not exclude studies based on the assessments. Detailed risk of bias results per study and domains are available in S1 Appendix.

### Classification of results

Prior to the study we considered it unlikely that we could synthesize or pool results, due to the many different ways inferences are investigated and the heterogeneity of the study populations. Thus, results are presented descriptively and thematically. In the discussion section we summarize the main misconceptions and fallacies that are supported by the results.

## Results

We identified 6,300 unique references of which 55 studies from the year 1963 and forward were included, see Flow chart in Fig 1. Studies were performed in different regions of the world: mostly in European countries, the United States, Australia and China, but there were also studies from South America and the Middle East.

Study populations were typically obtained by one of the following: 1) inviting a specific group (such as course participants, a department, a school/university, attendants to a scientific meeting etcetera); 2) contacting and inviting authors, editors or reviewers of scientific publications or 3) by openly inviting persons from a scientific community, such as members of association or a scientific social media group. The total number of participants in all included studies was more than 15,000. In the individual studies the number of participants ranged from 18 to 1,839 people.

The great majority of included studies were cross-sectional surveys using questionnaires for the assessment of inference. More recently published studies usually used online-questionnaires and applications while older studies used written questionnaires, either distributed at a specific occasion, such as at a course or meeting, or sent by mail.

Several different approaches and questions were employed to assess various aspects of inferences related to p-values, null hypothesis significance testing, and confidence intervals. Some instruments and questions have been used repeatedly, either in their original form or in variations, suggesting that those approaches are seen as relevant to many and that the questions and instruments have a fair and direct face validity. Such instruments often use questions with true or false response options about what logically can be deduced from the presented information. Other questions ask participants about their belief or confidence in a presented statement or research output, which on an aggregated level represents the subjective view of the respondents.

Some studies used questions and instruments that appear complex and difficult to understand, requiring respondents to assess or interpret results without having adequate or even sufficient information. There are examples of authors using questions which are unclear, not providing any correct response alternative and even erroneously claiming that a certain response alternative is correct, despite it being wrong. We present the ambiguities and controversies that we have found under a specific heading last in the result section.

Overall, there was substantial heterogeneity in how inference has been investigated in empirical studies. Where possible and informative, we report quantitative results, such as the proportions of participants providing correct or incorrect responses. However, differences in study populations, questions, and response formats often precluded direct quantitative comparisons across studies. Below we summarize and present the approaches and results according to different themes, arranged in chronological order of their emergence by publication time. Characteristics of included studies are presented in Table 1. A detailed tabulated presentation of each included study, the study population, the way the investigation of inferences was performed, its results and authors main conclusions are available in S2 Appendix, and a detailed text version of results is available in S3 Appendix.

**Table 1:** Characteristics and investigated misunderstandings, misconceptions and fallacies of included studies by year of publication.

Author	Year	Country	N^a^	Study population^a^	Type of inference investigated	Misunderstanding, misconception or fallacy supported by results^b^
Rosenthal et al.	1963	US	19	Psychology students and researchers	Significance test, degree of belief in research findings	
Beauchamp et al.	1964	US	20	Psycology students and researchers	Significance test, degree of belief in research findings	
Weiss et al.	1980	US	141	Physicians and medical staff	NHST	Misunderstandings of p-value and null hypotheses
Nelson et al	1986	US	85	Authors, reviewers and APA-members.	NHST, degree of belief in research findings	Cliffhanger effect
Oakes M.	1986	Great Britain	70	Academic psychologists	NHST, common misunderstandings	Inverse probability fallacy, fallacy of proof, fallacy of replication, error probability fallacy
Wulff et al.	1987	Denmark	148 + 97	Physicians + course participants	NHST, interpreting results of clinical vignettes	Inverse probability fallacy, fallacy of proof
Scheutz et al.	1988	Denmark	125 + 27	Dentists + Dental students	NHST, interpreting results of clinical vignettes	Inverse probability fallacy, fallacy of proof
Freeman, P	1993	UK	397	Doctors, dentists, and medical students	NHST	Inverse probability fallacy, fallacy of proof
Falk et al.	1995	Israel	53	Psychology students	NHST, common misunderstandings	Inverse probability fallacy, fallacy of proof
Ferill et al.	1999	US	707	Pharmacists	NHST and CI	Practical significance fallacy, inverse probability fallacy,
Mittag et al.	2000	US	225	AERA members	NHST, rated agreement with statements	
Vallecillos et al.	2000	Spain	436	University students over different disciplines	NHST	fallacy of proof
Gordon, H.	2001	US	113	AVERA members	NHST, rated agreement with statements	Practical Significance Fallacy, fallacy of replication
Poitevineau et al	2001	France	18	Post graduate psychology researchers	NHST, p-values relation to degree of confidence in of true treatment effect	
Haller et al.	2002	Germany	113	Students, scientific staff and teachers in statistics	NHST, common misunderstandings	Inverse probability fallacy, fallacy of proof, fallacy of replication, error probability fallacy
Lecoutre et al.	2003	France	45	Researchers in psychology + professional statisticians	NHST, interpreting situations when two studies present conflicting and non-conflicting results	
Cumming et al.	2004	International	263	Authors published in psychology, behavioral neuroscience, and medical journals	CI, anticipated replication	Replication Fallacy
Belia et al.	2005	International	473	Authors published in psychology, behavioral neuroscience, or medicine journals.	CI, interpretation of two visual CI:s in relation to statistical significance.	Confidence interval overlap fallacy,
Fidler, F.	2005	Australia	491	Four surveys in students in different fields and at different levels.	Comparing inferences of NHST and CIS	Confidence interval overlap fallacy, descriptive interval fallacy
Windish et al.	2007	US	277	Medical residents, post graduate levels.	NHST and CI, interpreting results of clinical vignettes	Inverse probability fallacy, error probability fallacy; use of CI:s for hypothesis testing
Kalinowski et al.	2008	Australia	120	Undergraduate students	NHST, common misunderstandings	Inverse probability fallacy, fallacy of proof, fallacy of replication
Fidler et al.	2009	Australia	136	Undergraduate students (same studies/study as in Fidler 2005)	NHST	
Castro Sotos et al.	2009	Belgium	144	Undergraduate students taking introductory courses in statistics	NHST	Inverse probability fallacy, fallacy of proof
Coulson et al.	2010	International	330	Authors of articles published in psychology, behavioral neuroscience, and medical journals	NHST and CI, comparing inferences from text and figures of significant and non-significant findings	
Monterde-i-Bort et al.	2010	Spain	36	Research professors at psychology with research and teaching experience	NHST, rated agreement with statements	
Westover et al.	2011	US	246	Physicians at teaching hospitals	NHST, true or false statements of common misunderstandings	Inverse probability fallacy, fallacy of proof
Polychronopoulou et al.	2011	Greece	127	Orthodontic postgraduate students in 10 European countries.	NHST, interpret at statistically non-significant finding	Inverse probability fallacy, fallacy of replication, error probability fallacy
Hoekstra et al	2012	Netherlands	66	Postgraduate students	NHST and CI, comparing inferences in confidence in and likelihood of replication.	
Lai et al.	2012	Australia	360	Authors of articles in high impact journals in psychology, statistics, and medicine.	NHST, replication	Fallacy of replication
Bookstaver et al.	2012	US	166	Participants in pharmacy recent programs	NHST, interpret at statistically non-significant finding	
Bezzina et al.	2014	UK mostly	80	European academics with document interest in research methodology	NHST and CI, two true or false statements	Inverse probability fallacy
Hoekstra et al.	2014	Netherlands	596	University students and researchers in psychology	CI, common misunderstandings	Probability fallacy, certainty fallacy replication fallacy
Msaouel et al.	2014	Greece	153	Residents in medical, surgical, or diagnostic and laboratory specialties.	NHST and CI, two multiple choice questions using clinical vignettes	Inverse probability fallacy, fallacy proof
Susarla et al.	2014	US	22	Plastic surgery residents	NHST and CI, two multiple choice questions using clinical vignettes	Inverse probability fallacy; use of CI:s for hypothesis testing
Badenes-Ribera et al.	2015	Spain	418	Academic psychologists	NHST, set of true or false statements	
Kühberger et al.	2015	Austria	214	Psychology students	NHST, understanding of the notion of significance in their relation to effect size and sample size.	
Garca-Perez et al.	2016	Spain	313	Undergraduate students in psychology	CI, true or false statements, many being misunderstandings	Inverse probability fallacy, fallacy of proof, fallacy of replication, error probability fallacy, magnitude fallacy
Badenes-Ribera et al.	2016	Spain	472	Academic psychologists	NHST, question about if statistically significance implies clinical significance	Practical Significance Fallacy
Badenes-Ribera et al.	2016	Italy	164	Academic psychologists	NHST, set of true or false statements	Inverse probability fallacy, fallacy of proof, fallacy of replication, error probability fallacy, magnitude fallacy
McShane et al.	2016	US	376	Authors published in high impact journals	NHST, dichotomization and likelihood judgments	Dichotomous Thinking Fallacy
Susarla et al.	2016	US	123	Plastic surgery residents	NHST and CI, two multiple choice questions using clinical vignettes	Inverse probability fallacy; use of CI:s for hypothesis testing
Badenes-Ribera et al.	2018	Italy	159	Academic psychologists	NHST, question about if statistically significance implies clinical significance	Practical Significance Fallacy
Kalinowski et al.	2018	Australia	101	University students	CI, assessment of subjective likelihood distributions	
Lyu et al.	2018	China	362	Students and researchers	NHST and CI, sets of true or false statements and comparison of results presented as NHST and CI-estimation	
McShane et al.	2018	US	257	Authors published in high impact journals	NHST, dichotomization and likelihood judgments	Dichotomous Thinking Fallacy
Tam et al.	2018	Australia	247	General practitioners	NHST, interpretation finding with a p = 0.05	Inverse probability fallacy, error probability fallacy
Ferreira et al.	2020	Portugal	153	Clinicians: physicians, nurses and other	NHST, common misunderstandings	Inverse probability fallacy, fallacy of proof, fallacy of replication, error probability fallacy
Lyu et al.	2020	China	1479	Scientists with various backgrounds and education	NHST and CI, sets of true or false statements and comparison of results presented as NHST and CI-estimation	
Alothman et al.	2020	Saudi Arabia	440	Researchers, residents, general practitioners	NHST and CI, two questions	
Hayat et al	2021	US	164	Nursing faculty members	CI	
Lytsy et al.	2022	Sweden	169	PhD-students, epidemiologists, and statisticians	NHST, common misunderstandings	Inverse probability fallacy, fallacy of proof,
Rajcani et al.	2023	Slovakia	65	Students, researchers, and lecturers	NHST and CI, true and false questions	Inverse probability fallacy, error probability fallacy, magnitude fallacy, difference in significance fallacy
Van den Akker et al.	2023	Netherlands	1839	Authors and editors of scientific journals	NHST, replication and belief in true effect	Inverse probability fallacy, error probability fallacy, magnitude fallacy, difference in significance fallacy
Vera-Ponce et al	2023	Peru	1192	University medical students	NHST, statements	

a) Number of responders if available; b) classified by the project group, if possible. Abbreviations: APA = American Psychological Association, AERA = American Educational Research Association, AVERA = American Vocational Education Research Association, CI = confidence interval, NHST = null hypothesis significance testing,

### Assessments of the degree of belief or confidence in research findings

Early inference studies examined the relationship between p-value levels and the “degree of belief” in research findings. In the first study, involving 19 psychology researchers and graduate students, participants rated their degree of belief across 14 different p-values and varying sample sizes. Belief increased with smaller p-values and larger sample sizes [27]. Two subsequent studies, involving 20 and 85 participants, respectively, reported similar findings [28,29]. The latter found evidence of a “cliffhanger effect” around conventional significance thresholds, although a later study with 18 participants suggested that the effect around  .05 may have been overstated [29,30].

### Assessments of level of agreement in statistical issues

Another approach examined participants’ agreement with statements about null hypothesis significance testing and other statistical issues using five-point Likert scales. In the first study, 225 members of the American Educational Research Association responded to a 29-item survey covering nine clusters of statistical perceptions [31]. Some items in this approach assessed factual knowledge, while others evaluated normative attitudes toward statistical concepts. Two subsequent studies used the same approach among researchers in education and psychology [32,33], resulting in a total of 374 participants across the three studies. Across the studies, responses indicated misunderstandings of several statistical concepts, and a comparative analysis of all three studies concluded that there was a concerning lack of understanding of fundamental methodological and statistical concepts [33].

### Interpreting results of NHST

Many of the studies included in this systematic review investigate inferences about p-values and results from null hypothesis significance testing (NHST) using true or false statements. A statement is true if it follows logically from the premises, and conversely it is false when it does not. Sometimes such statements are presented as general principles—for example that a statistically significant finding proves the null hypothesis is false—and sometimes embedded in clinical vignettes to mimic how results are encountered in the literature. These approaches have often been used to investigate common fallacies—such as the belief that NHST results provide probability measures of a hypothesis being true, indicate the likelihood of erroneous conclusions, or reliably predict replication outcomes.

Two surveys have been particularly influential and have been used either in their original form or in adapted versions. The first was published in a book by Michael Oakes in 1986. Seventy academic psychologists were presented with results from an independent *t*-test and asked to judge six statements as true or false [34]. All statements were intentionally false and reflected common misunderstandings about statistical inference. Three of the six statements were incorrectly endorsed as true by 60–86% of participants, and very few participants answered all six questions correctly. Based on these findings, the author concluded that many respondents demonstrated “a less than sound grasp of the conclusions that may properly be drawn from the results of a significance test.”

The second influential survey was published in 1995 by Falk and Greenbaum, who used an adapted version of Oakes’s questionnaire [18]. Respondents were asked to interpret the meaning of a statistically significant result at a predetermined significance level and were given the following response options: (1) “we proved that H_0_ is not true”; (2) “we proved that H_1_ is true”; (3) “we showed that H_0_ is improbable”; (4) “we showed that H_1_ is probable”; and (5) “none of answers 1–4 are correct.” Only 13.2% of participants correctly identified option (5), indicating widespread misunderstanding of statistical significance. These two surveys and the questions used are presented in Table 2.

**Table 2:** Three short questionnaires with true or false statements that have been used in several studies to investigate the prevalence of misconceptions of inference from null hypothesis significance testing (Oakes and Falk & Greenbaum) and results presented as confidence interval estimations (Hoekstra et al.). The correct answers to all response alternatives are “False”.

Oakes 1986	Falk and Greenbaum 1995	Hoekstra et al 2014
**Vignette**	**Vignette**	**Vignette**
Suppose you have a treatment which you suspect may alter performance on a certain task. You compare the means of your control and experimental group (say 20 subjects in each sample). Further, suppose you use a simple independent t test and your result is (t = 2.7, d.f. 18, p = 0.01). Please mark each of the following statements below as “true” or “false”.	The subjects were told that a test of significance was conducted, and the result turned out significant for a predetermined level. They were asked what is the meaning of such a result? Subjects were allowed to mark one or more answer as true.	Professor Bumbledorf conducts an experiment, analyzes the data and reports: “The 95% confidence interval for the mean ranges from 0.1 to 0.4!” Please mark each of the statements below as “true” or “false”. False means that the statement does not follow logically from Bumbledorf’s result. Also note that all, several, or none of the statements may be correct.
**True or false propositions**	**True or false propositions**	**True or false propositions**
You have absolutely disproved the null hypothesis (that there is no difference between the population means).	We proved that H_0_ is not true.	The probability that the true mean is greater than 0 is at least 95%.
You have found the probability of the null hypothesis being true.	We proved that H_1_ is true.	The probability that the true mean equals 0 is smaller than 5%.
You have absolutely proved your experimental hypothesis (that there is a difference between the population means).	We showed that H_0_ is improbable.	The “null hypothesis” that the true mean equals 0 is likely to be incorrect.
You can deduce the probability of the experimental hypothesis being true.	We showed that H_1_ is probable.	There is a 95% probability that the true mean lies between 0.1 and 0.4
You know, if you decided to reject the null hypothesis, the probability that you are making the wrong decision.		We can be 95% confident that the true mean lies between 0.1 and 0.4
You have a reliable experimental finding in the sense that if, hypothetically, the experiment were repeated a great number of times, you would obtain a significant result on 99% of occasions		If we were to repeat the experiment over and over, then 95% of the time the true mean falls between 0.1 and 0.4.

These two surveys have been used in whole or in part in several subsequent studies collectively encompassing over 4,000 respondents, including university students at all levels, researchers and health professionals across different fields, and methodologists, statisticians, and epidemiologists [35–47]. Misunderstandings were common but varied across studies and populations. For example, in one study using Oakes’s six questions, at least one incorrect response was given by 80.0% of methodology instructors, 89.7% of research psychologists, and all psychology students [35]. In two large Chinese samples, 99% and 89% of participants, respectively, gave at least one incorrect response to six questions concerning statistically significant findings [41,47]. In another study, only 10.7% of medical doctoral students and 12.5% of statisticians and epidemiologists correctly answered both questions concerning whether a statistically significant result provides proof of, or a probability statement about, a hypothesis [43].

Across these studies, commonly identified misconceptions included the inverse probability fallacy—the mistaken belief that the p-value represents the probability that the null hypothesis is true or false—and the fallacy of proof, whereby a statistically significant result is interpreted as definitive evidence that the null hypothesis is false or the alternative hypothesis is true. The replication fallacy, reflecting the belief that a significant result implies likely replication, was also frequently identified. Detailed study-level results are provided in the Supplementary Materials.

Apart from investigations using the questions of Oakes and Falk and Greenbaum, several other similar approaches have been employed. These include studies using clinical vignettes typically describing ‘statistically significant’ research findings followed by true/false questions about inference. Such approaches have been applied in samples of university and research students, dentists, pharmacists, and physicians, with a total of over 1,000 respondents [48–54]. The proportion selecting the intended correct interpretation varied substantially across studies. For example, 19% of 397 doctors, dentists, and medical students correctly interpreted a statistically significant finding [50], while studies using the same or adapted questions concerning a non-significant finding reported correct responses ranging from 33% to 59.6% [51–54]. Overall, these studies indicated substantial difficulties in interpreting results from NHST, although differences in questions and populations preclude direct quantitative comparison.

In addition to the studies mentioned above, several other investigations have employed various questions and methodologies to assess different aspects of inferences drawn from NHST. Lecoutre et al. examined how combinations of effect size and statistical significance influenced predictions about future studies, finding a strong inclination to be impressed by statistically significant results regardless of effect size [55]. Van den Akker et al. specifically investigated how significance labeling influenced belief in a theory, demonstrating that belief increased with the number of significant results and that direct replications carried more weight than conceptual ones [56]. Kühberger et al. took a different approach by asking students to estimate effect sizes based on significance labels alone, finding that significant results were intuitively associated with large effects and non-significant results with negligible ones [57]. Lai et al. focused specifically on p-value variability across replications, finding that researchers provided prediction intervals that were far too narrow, suggesting poor intuitive understanding of sampling variability [58].

Tam et al. used a mixed-methods approach among 247 Australian general practitioners, combining a dichotomous choice question with open-ended elaboration, and found that only 29% answered correctly; qualitative responses revealed that p-values were most commonly conceptualized as real-world probability statements about hypotheses or as simple thresholds for declaring superiority—reflecting both the inverse probability fallacy and dichotomous thinking [46]. Finally, McShane et al. conducted two studies in which p-values were experimentally manipulated in study summaries, demonstrating that researchers drew different conclusions about the same observed data depending on whether results crossed the significance threshold — even when explicitly asked to describe the sample rather than draw inferences — a pattern the authors interpreted as evidence of dichotomous thinking [59,60].

### Inferring clinical significance from results from NHST

Several studies have explored whether *clinical significance* can be inferred from *statistically significant findings*. Clinical significance refers to the practical importance or real-world relevance of a treatment effect. Determining clinical significance involves not only statistical results but also professional clinical judgment about whether the effect is meaningful for patient care. Thus, clinical significance cannot be inferred from statistical significance alone.

Some studies employed questionnaires assessing participants’ agreement with statements such as that *p* < 0.05 indicates that results are important [31–33]. These studies identified misunderstandings concerning the relationship between statistical significance and the importance of research findings.

Ferrill et al.—described previously—found that approximately 32.5% of 707 pharmacists and pharmacy students mistakenly believed that statistical significance guarantees clinical significance [61]. Badenes-Ribera et al. examined a similar misconception among 418 academic psychologists in Spain, of whom 35.2% incorrectly endorsed the statement that obtaining a statistically significant result implies that the detected effect is important [38]. In another study of 472 Spanish academic psychologists, the corresponding proportion was 30.3% among those who could name an effect-size statistic and 54.4% among those who could not [62]. These findings indicate that inferring practical or clinical importance from statistical significance occurs across different populations, although the proportion of respondents making this inference varies between studies.

### Questions about inferring confidence intervals

The interpretation of confidence intervals (CIs) has been examined using several distinct approaches. A pivotal contribution was made by Hoekstra et al., who developed a six-item vignette-based instrument that has since been used in several subsequent studies [63]. Participants completed a written survey describing a fictitious scenario in which a professor reports a 95% CI for the mean ranging from 0.1 to 0.4, and were asked to judge six statements about this interval as true or false. All statements were intentionally false, designed to mirror the approach previously used by Oakes for NHST (Table 2).

Administering the instrument to 562 Dutch students and researchers, they found that only about 2% correctly identified all statements as false, with no meaningful association between experience level and performance (r = 0.04). Subsequent studies reported similar difficulties. In a Spanish replication, approximately 50–80% of first-year students endorsed the individual false statements from the original instrument [64]. García-Pérez et al. also critiqued the original instrument on methodological grounds and introduced two additional statements they considered correct—a matter further discussed under Ambiguities and controversies below. Two studies by Lyu et al. found that about 90% of 362 Chinese participants endorsed at least one false statement in the first study; in a larger sample of 1,479 participants, 91–94% endorsed at least one false statement, depending on whether the CI scenario was statistically significant or non-significant [41,47].

A second approach asked participants to make graphical or predictive judgments about CIs. Cumming et al. investigated researchers’ understanding of CIs and error bars across two studies involving a total of approximately 740 participants drawn from psychology, behavioral science, and medicine [65,66]. Findings revealed what the authors termed the “confidence level misconception”—the belief that approximately 95% of future means would fall within the original 95% CI, when simulation indicates the true figure is closer to 83.4%—as well as systematic errors when adjusting CIs to achieve statistical significance. Kalinowski et al. extended this line of inquiry by examining students’ intuitions about the probability distribution underlying a CI, identifying several competing misconceptions about the relative likelihood of parameter values inside and outside the interval [67].

A third approach embedded CI questions within broader biostatistics assessments. Across studies involving medical residents, plastic surgery residents, and nursing faculty in the USA, correct response rates to CI questions ranged from approximately 12% to 46% [51,68–70]. An earlier study among 707 pharmacists and pharmacy students found that only 22.9% correctly answered a multiple-choice question about the characteristics of CIs [61], though the interpretation of the correct answer in that study is disputed and discussed further under Ambiguities and controversies below.

Collectively, approximately 12 studies involving over 5,700 participants across students, researchers, and clinicians in multiple countries have investigated the interpretation of confidence intervals. Despite differences in design and population, the studies consistently identified difficulties in interpreting CIs, although the prevalence and types of misconceptions varied. Common misconceptions included interpreting a 95% CI as implying a 95% probability that the true parameter lies within the observed interval, and misconceptions about what the interval implies for future replications. Notably, greater experience or seniority did not consistently correspond to better performance. Overall, the findings indicate that misinterpretations of confidence intervals occur across disciplines and career stages, with substantial proportions of participants demonstrating difficulties in correctly interpreting CIs.

### Approaches to compare inferences of NHST and CIs

Another approach examines whether misinterpretations are reduced when results are presented as CIs rather than NHST. Fidler, across a thesis and a subsequent published study involving Australian university students, found that 39% of participants interpreted non-significant results as indicating “no effect” when results were presented using NHST, compared with 13% when presented using CIs [71,72]. Coulson et al. extended this to published researchers across psychology, behavioral neuroscience, and medicine. In their first study (n = 330), interpretations were generally poor regardless of format. However, among participants presented with CIs, 94.7% of those who interpreted the results without reference to NHST correctly regarded the two study results as similar, compared with 40.0% of those who invoked NHST in their interpretation. A second study involving 50 academic psychologists showed a similar pattern [73]. Hoekstra et al. varied both presentation format and p-value level among 66 PhD students, finding fewer inferential errors under CI presentation, but also lower confidence in the alternative hypothesis and lower estimates of replicability compared with NHST presentation. Contrary to the authors’ expectations, a cliff effect was observed more frequently in the CI scenarios [74].

Collectively, across studies involving approximately 1,000 participants including students, researchers, and clinicians, these findings suggest that CI presentation may reduce certain misinterpretations relative to NHST but does not eliminate inferential errors. and may introduce different ones.

### Ambiguities and controversies

#### Ambiguities about inferences of results from null hypothesis significance testing.

The literature on empirical investigations of inferential reasoning often aims to highlight common errors and misconceptions in interpreting statistical inferences. However, it can also contribute to ambiguities, uncertainties, and even controversies. One reason for this is that the underlying concepts and terminology are frequently vague and poorly defined. Null Hypothesis Significance Testing (NHST) is a procedure that combines p-values with significance levels (α) used in hypothesis testing. This merging of two distinct approaches—significance testing, which gauges the strength of evidence against a null hypothesis, based on the observed data, and hypothesis testing at a prespecified α-level to produce a binary decision can lead to confusion when interpreting statistical outcomes and their implications. Several of the included studies claim to investigate misunderstandings about p-values, while their actual focus is wider, examining inferences from p-values within the context of results from NHST [38,46,50,62,75]. For example, in the article published by Tam et al. participants are presented with a vignette of a tested treatment where the authors state that a test shows that a new drug was superior to the old drug at lowering blood pressure (p = 0.05) [46]. Respondents were then asked to interpret what p = 0.05 means. While some may understand the vignette as presenting a statistically significant result, others may question this interpretation because no α-level is specified.

There are also examples of questions for which the intended interpretation is ambiguous. For example, Mittag et al. asked respondents to rate on a five-point Likert scale whether they agreed that “Finding that p <  .05 is one indication that the results are important.” [31]. Although statistical significance does not by itself establish practical or clinical importance, the wording “one indication” leaves room for different interpretations. Consequently, disagreement with the statement is not necessarily the only defensible response, making responses difficult to classify unambiguously as correct or incorrect.

A more serious concern is that some studies appear to contain questions or designated answers that are inconsistent with standard statistical definitions. In an article by Alothman et al., respondents were asked “If you reject the null hypothesis at 10% then a) it will be rejected at 5%, b) it could be rejected or accepted at 5% c) it can’t be rejected at 5%” [76]. The question is problematic in the context of conventional hypothesis testing because the significance level is normally specified before examining the test result. The same study also asked participants what the p-value means and provided the following response alternatives a) probability to reject the null hypothesis when it’s true. b) chance of accept null hypothesis when it’s true. c) probability of reject alternative when it’s true. None of these options corresponds to the conventional definition of a p-value, although the authors designated alternative (a) as correct.

In two publications by Badenes-Ribera et al., participants were presented with ten statements, eight of which represented common fallacies about conclusions drawn from Null Hypothesis Significance Testing (NHST) [38,39]. The vignette provided was as follows: “Suppose that a research article indicates a value of p = 0.001 in the results section (α = 0.05). Select the true statements. The authors identified the following statements as correct: “The probability of the result of the statistical test is known, assuming that the null hypothesis is true” and “Given that p = 0.001, the result obtained makes it possible to conclude that the differences are not due to chance.” We consider both designated answers problematic. A p-value is the probability, under the null hypothesis, of obtaining a test statistic at least as extreme as the observed one; it is not simply the probability of the observed test result. Furthermore, a small p-value does not establish that an observed difference “is not due to chance”. Thus, responses to these items cannot straightforwardly be interpreted as evidence of participants’ misconceptions.

In the studies by McShane et al., participants were asked a question regarding differences in means between two groups in a sample [59,60]. As these differences stem from an intervention and an implicit randomized experiment (drug A vs drug B), the question implicitly refers to causal effects. With causal effects, inference may also be relevant within the sample. This then means that the p-values are not redundant, as was the maintained assumption under model-based inference in [59,60]. Thus, researchers responding that the differences were greater with lower p-values may not necessarily have been reasoning incorrectly. The authors also asked participants whether they assumed any specific statistical model when considering the question about differences in the sample. Most respondents indicated that they did not use any specific model, and only a few mentioned a permutation approach based on the randomization assumption.

#### Ambiguities about inferences of confidence intervals.

There are also questions and statements about confidence intervals that may introduce ambiguity or promote questionable interpretations.

In a 1999 study, Ferrill et al. presented the following statement about confidence intervals as a correct interpretation: “Confidence intervals provide an estimate of the range where the true mean value in the affected population is likely to reside” [61]. While this is a vague statement, perhaps supported by intuitive heuristics, it does not correspond to the conventional frequentist interpretation of a confidence interval, because it appears to assign a likelihood to the fixed population parameter on the basis of the observed interval.

In an article by Bezzina et al., authors presented the following statement about confidence intervals as true: “a confidence interval is a statement about the unknown population parameter” [77]. This statement is ambiguous from a frequentist perspective. The defining property of a frequentist confidence interval concerns the long-run coverage of the procedure: over repeated sampling, intervals constructed using a valid 95% confidence procedure will contain the true parameter in 95% of repetitions. This does not imply that a particular observed 95% confidence interval has a 95% probability of containing the fixed population parameter.

In an article by Msaouel et al, the authors identified as the most correct interpretation of a relative risk with a 95% confidence interval from 1.10 to 2.45 is that “there is a 95% likelihood that the true relative risk falls between 1.10 and 2.45” [45]. Although this may have been the best of the four response alternatives provided, it assigns a probability to the fixed parameter and is therefore not a correct interpretation of a confidence interval under the conventional frequentist framework.

Another example is the article published by García-Pérez et al., a study described above [64]. The study used the same questionnaire as Hoekstra et al [63]. The vignette used was a statement from a researcher who, after conducting an experiment analyzing the data, reports that the 95% CI for the mean ranges from 0.1 to 0.4. The original questionnaire includes six false statements (see Table 2) and García-Pérez et al, therefore, added two statements which they claimed are correct interpretations of a confidence interval. These were*: “The claim ’The true mean lies between 0.1 and 0.4‘ is true with probability 0.95”* and *“The data are compatible with the notion that the true mean lies between 0.1 and 0.4”.* The validity of these statements has been disputed [6,78]. In particular, the first assigns a probability of 0.95 to the fixed population parameter after the interval has been observed, which is not supported by the conventional frequentist interpretation of confidence intervals. Consequently, participants’ responses to these items cannot straightforwardly be classified as demonstrating correct or incorrect understanding.

We have not reported results from one study [75]. In this study, the authors surveyed knowledge about p-values in 1,192 Peruvian students using 11 true-or-false questions. Several of the statements were unclear, and the authors did not indicate which responses they regarded as correct, while reporting only the proportions of students classified as answering each question correctly. Consequently, the reported proportions cannot be independently interpreted or evaluated.

## Discussion

We conducted a systematic review to identify studies examining how researchers, educators, students, and professionals understand, infer, and interpret results from null hypothesis significance testing (NHST)—such as p-values and statistically significant findings—as well as from estimation approaches, such as confidence intervals.

Our search identified 55 studies conducted over six decades, involving diverse populations such as undergraduate and postgraduate students, PhD candidates, researchers, clinical professionals, and educators in methodology and statistics. These studies employed various approaches, including examining how NHST results influence confidence in research findings, assessing agreement with inferential statements, and evaluating expectations about the replication of p-values. Several studies used rigorous methods, asking participants to evaluate true or false statements that could be logically deduced from given assumptions. Such approaches effectively assess common inferential misunderstandings.

Inferences drawn from confidence intervals have also been studied, though less extensively than those from NHST. Methods used include assessing whether respondents understand how confidence intervals can be used for hypothesis testing, predicting the replication of confidence intervals, and evaluating when multiple intervals indicate agreement or conflict in terms of statistical significance. Several studies used questionnaires with true or false statements to investigate common misunderstandings. Additionally, some approaches compared inferences drawn from NHST results with those based on confidence interval presentations.

Due to substantial variability in study populations, approaches, and questions posed to address inferences, we did not find it feasible to synthesize the results. However, we consider the following conclusions about misunderstandings and fallacies to be valid regarding inferences of results from NHST.

### Misconceptions and fallacies when inferring NHST and p-values

Misunderstandings about the implications of results from NHST are widespread across studies conducted in various regions and among diverse populations, including individuals with substantial educational backgrounds and experience in statistics. The authors of these studies consistently conclude that there is a general lack of understanding regarding NHST, highlighting that these misconceptions are surprisingly common.

One of the most common misunderstandings is the inverse probability fallacy, the mistaken belief that a result from null-hypothesis significance testing—such as a p-value or a statistically significant finding—directly tells us the probability that the null hypothesis is true given the observed data. In its most extreme form, fueled by dichotomous thinking, this fallacy leads many to believe that a p-value below a fixed threshold proves the alternative hypothesis or conclusively refutes the null hypothesis. More broadly, this misconception reflects the false belief that the risk of reaching an erroneous conclusion can be calculated from the data of a single experiment, without considering external evidence, such as the plausibility of the underlying mechanism [5]. Another common misconception is *the replication fallacy*, which occurs when people mistakenly believe that a low p-value or a statistically significant finding is likely to be replicated in a future study. It has been argued that statistical significance is not a reliable predictor of replicability [66,79]. Furthermore, large scale replications studies in the field of economics [80], preclinical cancer biology [81] and psychology [82,83] have demonstrated high rates of replication failures, highlighting that replication issues may also depend on publication bias and selective reporting. It has been argued that the statistical ritual creates an illusion of certainty of not only an existing effect, but also for the not statistically tested alternative hypothesis, which make successful replication appear to be certain or almost certain [84].

Two common and related misconceptions are the effect size fallacy and the clinical significance fallacy. The effect size fallacy involves the incorrect assumption that a low p-value or statistical significance automatically implies a large or meaningful effect size. Such an interpretation is not warranted as p-values are inherently confounded, since they conflate information about the effect size with the sample size [85]. The clinical significance fallacy occurs when people confuse statistical significance with clinical significance – assuming that a statistically significant result automatically means that the effect or treatment has meaningful benefits for patients or clinical practice. Notably, a statistically significant result can correspond to a very small and trivial effect in studies with large sample sizes.

A related flaw in reasoning is the tendency for confidence in research findings to rise as p-values shrink and sample sizes grow, irrespective of the true strength of the evidence. Moreover, there is ample evidence of a “cliff-hanger” effect, in which our confidence jumps abruptly at arbitrary thresholds (for example, p = 0.049 versus p = 0.051), fostering all-or-nothing interpretations. McShane et al. [59,60] provide a striking demonstration that even those with formal statistical training and extensive research experience fall prey to this dichotomization, leading to errors in the interpretation of evidence. A related fallacy is the difference-in-significance fallacy: the erroneous belief that the difference between a ‘significant’ and a ‘not significant’ finding is itself statistically significant [86].

It should also be noted that the self-assessed confidence in answers, which is analyzed in several studies, is often not well correlated with accuracy; sometimes higher confidence is associated with making more errors.

None of the above misunderstandings are new; they have been identified and discussed in several earlier articles [6–8,18,19]. The main fallacies observed from inferences of p-values, statistical significance and results from null hypothesis significance testing are summarized in a table in Supplement 1. Note that there is no consistent terminology for how fallacies should be named, and that other authors may use different terms.

### Misconceptions and fallacies when inferring confidence intervals

While studies of inference of confidence interval are less common, several studies across different regions and countries, and targeting different populations are consistent in their findings. Misunderstandings about how confidence intervals should be inferred are also prevalent in all investigated populations including persons with long experience and substantial educational backgrounds.

Just as many mistakenly treat p-values and “statistical significance” as direct probability measures for hypotheses, similar errors occur with confidence intervals – namely, treating the single, data-based interval observed as a definitive locator for the unknown true parameter. This serves as a starting point to some fallacies that resemble each other in reasoning but differ in wording and that are subject to heated scholarly debate. *The probability fallacy* (also called The Fundamental Confidence Fallacy by [78]) means that the chosen confidence level (often 95%) provides the range where the true mean value in the population is likely to be located, or that there is a specific probability (e.g., 95%) that the true parameter lies within the observed interval. This reasoning is however flawed, since Neyman-Pearson model-based theory does not support probabilistic statements about the parameter after observing the data. The observed interval either contains the parameter or it does not; there is no probability attached after the data are observed.

For the same reason it is debated if one can use other words, such as whether a chosen confidence level translates to that it is likely, reasonable, plausible or that *one* should have confidence in that the observed interval contains the true value. In our systematic review, we identified several studies where the authors claim that a confidence interval is a statement about the true parameter value [61,64,77] and that a 95% confidence interval has a 95% likelihood or probability of containing the true value. Morey et al have disputed such interpretations [78] and provided insightful explanations in subsequent articles of their investigation of misinterpretation of confidence intervals [87,88].

Neyman himself was explicit that the practical use of confidence intervals does not involve inductive reasoning about the parameter, but rather an act of will — a deliberate decision to adjust our behavior in accordance with the confidence limits (see page 210 in [25]). Thus, according to Neyman, confidence intervals are justified to guide actions (including guideline and treatment recommendations) as they, in the long-run relative frequency will be adjusted correctly to the chosen confidence level.

The challenges of interpreting confidence intervals have been highlighted in several articles [6,78] and it has been proposed to replace the terms “confidence” and “significance” with “compatibility” and “surprise” to better capture what the data actually convey [89,90]. In line with Neyman’s original word of caution, some have even questioned whether confidence intervals can be meaningfully interpreted at all [91]. Yet others have discussed, without agreement, whether confidence intervals are better termed “uncertainty intervals”. It is, however, debated whether calling them “uncertainty intervals” represents real progress, arguing that this label may still mask unaccounted sources of error – such as bias or prior beliefs – despite its more transparent wording [92]. The term compatibility intervals, advocated by several authors as a better alternative to confidence intervals [89,90], reflects a shift toward a more modest and less universal interpretation: rather than attempting to pinpoint an unobservable “true” parameter, an observed interval denotes the range of values that are compatible with the observed data under the assumed model.

There is also a misunderstanding, sometimes referred to as the replication fallacy, which is the erroneous belief that confidence intervals predict the results of future replications. The confidence interval is about the probability of containing the true parameter under repeated sampling, not about the outcome of future studies or the interval in those studies. Additionally, confidence intervals are often misused for hypothesis testing. There is confusion about how confidence intervals relate to hypothesis testing, i.e., how confidence intervals can be employed for hypothesis testing to assess statistical significance. Furthermore, many researchers encounter substantial challenges in determining whether two confidence intervals genuinely conflict. A common misconception is that non-overlapping intervals indicate statistical significance, which is not always the case.

We have tried to summarize the main fallacies of inferences regarding confidence interval in a table available in supplement 1. Note again, that other authors may use different terms or phrasings to describe the same misconceptions.

It has for long been argued that estimation of effect sizes with their accompanying confidence intervals are superior to reporting results with p-values and null hypothesis significance testing [21,22,93], an advice also presented in ASA’s official statement The ASA Statement on p-values: Context, Process, and Purpose which urge researchers to report effect sizes and confidence intervals to capture both magnitude and precision of effect [20]. Results from studies comparing inferences based on confidence intervals with those from null hypothesis significance testing indicate that using estimates and confidence intervals leads to fewer inferential mistakes. When results are presented with confidence intervals, respondents are less likely to make definitive statements about the presence or absence of an effect, and more likely to discuss the range of possible values for the effect size [73,74]. Despite these advantages, misunderstandings about confidence intervals are still common, with a high proportion of respondents overstating the certainty that the interval contains the true parameter.

The ASA’s 2016 statement on p-values [20] helped establish a common foundation for interpreting statistical results by demystifying the p-value and cautioning against its misuse. It emphasized that statistical significance is not synonymous with practical or scientific importance, and that p-values must be interpreted within the broader context of study design and evidence – rather than treated as definitive indicators of truth or falsehood. The shift from NHST to the use of confidence intervals reflects a broader change in statistical reasoning. However, as demonstrated by the results of this systematic review, simplistic heuristics, misconceptions, and interpretive fallacies remain widespread.

Confidence intervals in systematic reviews and meta-analyses are integral to frameworks such as GRADE (Grading of Recommendations Assessment, Development and Evaluation), where they inform judgments about the certainty that the true effect lies within a specified range – or on one side of a critical threshold [94]. These thresholds may represent a null effect, permitting statistical testing, or a minimally important difference (MID) [95,96]. When confidence intervals are used to judge whether an effect exceeds a threshold other than zero, interpretation shifts from statistical testing to a form of “visual testing”.

There is clear value in a broad, structured discussion on how confidence intervals should be interpreted and used in the context of decision-making and guideline development, including the role of statistical testing. We thus encourage the American Statistical Association or another reputable statistical organization to undertake a similar initiative, promoting discussion and ultimately producing a formal statement on the interpretation and proper use of confidence intervals in research and decision-making.

### Limitations

This systematic review has some notable limitations. Although our goal was to comprehensively identify all relevant studies, the diversity of terminology and keywords used by different authors likely means that our literature search overlooked some work. Reviewing the reference lists of included studies may have mitigated this problem by capturing the most frequently cited articles, but gaps may remain. Although a research plan was developed at the outset of the project, it was not prospectively registered, which limits the possibility of independently verifying that the review methods were specified in advance.

We assessed each study’s risk of bias using a simplified framework, focusing on how study populations were selected and invited, response and non-response rates to key items, and whether established instruments were employed. Many surveys exhibited very low, low, or unknown response rates and restricted analyses to complete responders, undermining the generalizability of their findings. Because participants are assumed to have greater interest in—and knowledge about—statistical methods than non-participants, errors and fallacies may be even more prevalent than our results suggest.

Finally, the heterogeneity in populations, methodologies, and instruments precludes meaningful pooling of results and, for the same reason, any formal analysis of whether misconception prevalence has changed over time. To facilitate such comparisons in the future, there is a clear need to develop and validate standardized methods for assessing inferential reasoning. One might expect that the intensified methodological debate following the replication crisis around 2015 would have led to a measurable reduction in misconception prevalence, but nothing in the available evidence suggests any obvious decline.

## Conclusion

To our knowledge, this is the most comprehensive systematic review of how researchers, educators, students, and professionals understand and interpret *p*-values, statistical significance, and confidence intervals. We included 55 studies involving thousands of participants with diverse educational backgrounds and expertise, using a wide range of specifically designed inference tasks.

Despite differences in study design and populations, findings across studies were strikingly consistent: misunderstandings and fallacies related to null hypothesis significance testing and confidence intervals are widespread and persistent—even among those with advanced statistical training. Common errors include the inverse probability fallacy, the belief that statistical significance implies a large or meaningful effect, confusion between statistical and clinical significance, and misinterpretation of confidence intervals as probabilistic statements about true values.

These pervasive misconceptions point to a systemic issue in statistical education and scientific communication. Our findings underscore an urgent need to improve statistical training, promote more nuanced interpretations of statistical results, and encourage reforms in how statistical evidence is presented in scientific research. Clearer guidance, such as offered by the ASA’s 2016 p-value statement, may be equally necessary for confidence intervals to support sound inference and decision-making.

## Supporting information

S1 Appendix Supporting information for systematic review methods and results. Contains literature search strings, list of excluded studies with reasons for exclusion, risk of bias assessments for included studies, and summary tables of identified misconceptions related to p-values, NHST, and confidence intervals.
(DOCX)

S2 Appendix Included studies tabulated by research question. Presents all included studies in alphabetical order by first author, organized by research focus: studies on inferences from null hypothesis significance testing and studies on inferences from confidence intervals.
(DOCX)

S3 Appendix Detailed results. Provides a comprehensive presentation of results from all included studies, supplementing the synthesized findings reported in the main manuscript.
(DOCX)
