# Supplementary material for: Inferences of p-values, statistically significant results, and confidence intervals – A systematic review of empirical studies
Source: PLoS One. 2026 Nov 11;21(11):e0362071. doi: 10.1371/journal.pone.0362071 (PMC13754157; doi:10.1371/journal.pone.0362071)
Supplement: Supplementary file 1 [file pone.0362071.s004.pdf]

The manuscript presents a comprehensive and well-executed systematic review that documents the pervasive nature of statistical misunderstandings.
The authors have undertaken substantial work confirming a persistent problem in statistical literacy. However, the manuscript would better serve readers through a more streamlined presentation with synthesized findings and concrete solutions to address the identified problems.

Mayor
I recommend:
- Moving the extensive, study-by-study descriptions to supplementary materials
- Organizing findings thematically with quantitative summaries (although I understand the difficulties posed by heterogeneity)

Minor
- **Line 147:** Consider using "p-value" consistently throughout (lowercase "p") rather than "P-value" for standardization.
- **Lines 203-207:** Please provide frequencies or percentages for each category mentioned
- **Line 208:** When reporting range (18 to 1,839 participants), please also provide measures of central tendency and variability.
- **Line 209:** "Study designs were typically cross-sectional surveys..." Please quantify "typically" (e.g., "X studies (Y%) used cross-sectional designs...").
- **Lines 210-234 and Results section generally:** Throughout the results narrative, please supplement qualitative descriptions with quantitative summaries where possible to help readers better understand the distribution of study characteristics.
- **Lines 1031-1035:** There appears to be repetition in this passage. Please revise to eliminate redundancy.
