# Supplementary material for: Inferences of p-values, statistically significant results, and confidence intervals – A systematic review of empirical studies
Source: PLoS One. 2026 Nov 11;21(11):e0362071. doi: 10.1371/journal.pone.0362071 (PMC13754157; doi:10.1371/journal.pone.0362071)
Supplement: Supplementary file 2 [file pone.0362071.s001.docx]

Supplement 1

Contents

[Literature searches	2](#_Toc216789101)

[Studies not included after being read in full	6](#_Toc216789102)

[Assessment of Risk of bias in included studies	9](#_Toc216789103)

[Summary of misconceptions and fallacies related to inferences from p-values, statistically significant findings, and other aspects of NHST	12](#_Toc216789104)

[Summary of misconceptions and fallacies related to inferences from confidence intervals and comparisons	14](#_Toc216789105)

# Literature searches

A literature search was performed in the databases Cinahl, Pubmed, Psychinfo and Web of Science in December 2021 and was updated in April 2023, in August 2024 and in August 2025 using the same search strings of index terms and free text. Below is the search strategy of the last updated search.

**Database:** *Cinahl* **Date: 2025-08-08**

| Search nr | Term | Results | Comment |
| --- | --- | --- | --- |
| *Concept 1* | | | |
| #1 | **(MH "P-Value") OR (MH "Significance Test")** | 118,352 |  |
| #2 | CI OR "confidence interval*" OR interpretation* OR NHST OR "null hypothesis significance testing*" OR "p value*" OR "probability value" OR "significance test*" OR data analys* OR inference* OR interpretation* OR signifiance  **[Title/Abstract]** | 878,747 |  |
| #3 | #1 OR #2 | 974,067 |  |
| *Concept 2* | | | |
| #4 | deduction* OR erroneousness OR fallacy OR misapprehension* OR misconception* OR misconjectur* OR misinterpre* OR misrepresentation* OR misunderstanding* OR plausibility  [Title/Abstract] | 15,302 |  |
| *Concept 3* | | | |
| #5 | statistic*  [All fields] | 1,412,563 |  |
| *Final results* | #3 AND #4 AND #5 | 1,342 |  |
|  | Limiters - Published Date:  2024-08-26 - 2025-12-31 | 117 |  |

**Database:** *Pubmed* **Date: 2025-08-08**

| Search nr | Term | Results | Comment |
| --- | --- | --- | --- |
| *Concept 1* | | | |
| #1 | "Confidence Intervals"[Mesh] OR "Data Interpretation, Statistical"[Mesh] | 93,741 |  |
| #2 | CI OR "confidence interval*" OR interpretation* OR NHST OR "null hypothesis significance testing*" OR "p value*" OR "probability value" OR "significance test*" OR data analys* OR inference* OR interpretation* OR signifiance  **[Title/Abstract]** | 1,977,297 |  |
| #3 | #1 OR #2 | 2,039,167 |  |
| *Concept 2* | | | |
| #4 | deduction* OR erroneousness OR fallacy OR misapprehension* OR misconception* OR misconjectur* OR misinterpre* OR misrepresentation* OR misunderstanding* OR plausibility  [Title/Abstract] | 53,866 |  |
| *Concept 3* | | | |
| #5 | statistic*  [All fields] | 3,068,617 |  |
| *Final results* | #3 AND #4 AND #5 | 2,436 |  |
|  | #6 AND 2024/08/26:3000/12/12[pdat] | 241 |  |

**Database:** *PsycInfo* **Date: 2025-08-08**

| Search nr | Term | Results | Comment |
| --- | --- | --- | --- |
| *Concept 1* | | | |
| #1 | CI OR "confidence interval*" OR interpretation* OR NHST OR "null hypothesis significance testing*" OR "p value*" OR "probability value" OR "significance test*" OR data analys* OR inference* OR interpretation* OR signifiance  **[Title/Abstract]** | 667,604 |  |
| *Concept 2* | | | |
| #2 | deduction* OR erroneousness OR fallacy OR misapprehension* OR misconception* OR misconjectur* OR misinterpre* OR misrepresentation* OR misunderstanding* OR plausibility  [Title/Abstract] | 30,888 |  |
| *Concept 3* | | | |
| #3 | statistic*  [All text] | 456,307 |  |
| *Final results* | #1 AND #2 AND #3 | 1,151 |  |
|  | Limiters - Published Date:  2024-08-26 - 2025-12-31 | 36 |  |

**Database:** *Web of Science* **Date: 2025-08-08**

| Search nr | Term | Results | Comment |
| --- | --- | --- | --- |
| *Concept 1* | | | |
| #1 | CI OR "confidence interval*" OR interpretation* OR NHST OR "null hypothesis significance testing*" OR "p value*" OR "probability value" OR "significance test*" OR data analys* OR inference* OR interpretation* OR signifiance  **[Title/Abstract]** | 6,541,627 |  |
| *Concept 2* | | | |
| #2 | deduction* OR erroneousness OR fallacy OR misapprehension* OR misconception* OR misconjectur* OR misinterpre* OR misrepresentation* OR misunderstanding* OR plausibility  [Title/Abstract] | 133,975 |  |
| *Concept 3* | | | |
| #3 | statistic*  [All fields] | 3,197,653 |  |
| *Final results* | #1 AND #2 AND #3 | 4,338 |  |
|  | *Timespan: 2024-08-26 to 2025-12-31 (Publication Date)* | 341 |  |

# Studies not included after being read in full

| **Study reference** | **Main reason for non-inclusion** |
| --- | --- |
| Aczel B, Palfi B, Szollosi A, et al. Quantifying Support for the Null Hypothesis in Psychology: An Empirical Investigation. *Advances in Methods and Practices in Psychological Science*. 2018;1(3):357-366. doi:10.1177/2515245918773742 | Wrong outcome |
| Aczel B, Bago B, Szollosi A, Foldes A and Lukacs B (2015) Is it time for studying real-life debiasing? Evaluation of the effectiveness of an analogical intervention technique. *Front. Psychol.* 6:1120. doi: 10.3389/fpsyg.2015.01120 | Wrong outcome |
| Blanco G. Tracey and Chamberlin, Scott A. (2019) "Pre-service teacher statistical misconceptions during teacher preparation program," *The Mathematics Enthusiast*: Vol. 16 : No. 1 , Article 24. | Wrong outcome |
| Branch, M. (2014). Malignant side effects of null-hypothesis significance testing. *Theory & Psychology*, *24*(2), 256-277. https://doi.org/10.1177/0959354314525282 (Original work published 2014) | Theoretical /not empirical study |
| Brown, Jonathan Student understanding of the hypothetical nature of simulations in introductory statistics; M.  2022;83(), Thesis | Wrong outcome |
| Budé L, van de Wiel MW, Imbos T, Berger MP. The effect of directive tutor guidance on students' conceptual understanding of statistics in problem-based learning. Br J Educ Psychol. 2011 Jun;81(Pt 2):309-24. doi: 10.1348/000709910X513933. Epub 2011 Mar 9. PMID: 21542821. | Wrong outcome |
| Croucher, John; Hon, Stephnie. (2022). Data Science in Medical Research. International Medical Journal (1994). 28. 650-653. | Wrong outcome |
| Delgado-Gómez, D.; González-Landero, F.; Montes-Botella, C.; Sujar, A.; Bayona, S.; Martino, L. Improving the Teaching of Hypothesis Testing Using a Divide-and-Conquer Strategy and Content Exposure Control in a Gamified Environment. *Mathematics* **2020**, *8*, 2244. https://doi.org/10.3390/math8122244 | Wrong outcome |
| El Tecle NE, Urquiaga JF, Griffin ST, Alexopoulos G, El Ahmadieh TY, Aoun SG, Mattei TA. Misinterpretations of Null Hypothesis Significance Testing Results Near the P-Value Threshold in the Neurosurgical Literature. World Neurosurg. 2022 Mar;159:e192-e198. doi: 10.1016/j.wneu.2021.12.030. Epub 2021 Dec 14. PMID: 34915206. | Wrong population |
| Fabby, Carol, Identifying student difficulties in conditional probability within statistical reasoning 2022;83(), Thesis | Wrong outcome |
| Fisk, J. E. (2005). Age and Probabilistic Reasoning: Biases in Conjunctive, Disjunctive and Bayesian Judgements in Early and Late Adulthood. *Journal of Behavioral Decision Making, 18*(1), 55–82. https://doi.org/10.1002/bdm.488 | Wrong outcome |
| Ganasegeran, K.; Chang, A.S.H.; Jamil, M.F.A.; Looi, I. Clinicians’ Perceived Understanding of Biostatistical Results in the Medical Literature: A Cross-Sectional Study. *Medicina* **2019**, *55*, 227. https://doi.org/10.3390/medicina55060227 | Wrong focus /not about inference |
| Gao J. P-values - a chronic conundrum. BMC Med Res Methodol. 2020 Jun 24;20(1):167. doi: 10.1186/s12874-020-01051-6. PMID: 32580765; PMCID: PMC7315482. | Theoretical /not empirical study |
| Gibbison, B. (2017), Fifty percent of anaesthetists are worse than average at understanding statistics and risk. Anaesthesia, 72: 118-121. https://doi.org/10.1111/anae.13690 | Theoretical /not empirical study |
| Glorfeld, L. W.; Finnell, C. A A study of students' understanding of some elementary statistical concepts: Indications of shortcomings; 1997 Annual Meeting of the Decision-Science-Institute Nov 22-25 1997;():982-984  San Diego, Ca 1997 Nov 22-25 | Abstract only |
| Hagtvedt, R., Jones, G.T. and Jones, K. (2008), Teaching Confidence Intervals Using Simulation. Teaching Statistics, 30: 53-56. https://doi.org/10.1111/j.1467-9639.2008.00308.x | Wrong focus/not about inference |
| Hemming K, Javid I, Taljaard M. A review of high impact journals found that misinterpretation of non-statistically significant results from randomized trials was common. J Clin Epidemiol. 2022 May;145:112-120. doi: 10.1016/j.jclinepi.2022.01.014. Epub 2022 Jan 23. PMID: 35081450. | Wrong outcome |
| Kline, R. B. (2013). Cognitive distortions in significance testing. In R. B. Kline, *Beyond significance testing: Statistics reform in the behavioral sciences* (2nd ed., pp. 95–120). American Psychological Association. https://doi.org/10.1037/14136-004 | Theoretical /not empirical study |
| Lane-Getaz, Sharon Jacqueline, Development and validation of a research-based assessment: Reasoning about P-values and Statistical Significance  2007;68():2422-2422, Thesis | Wrong study design |
| Mavros MN, Alexiou VG, Vardakas KZ, Falagas ME. Understanding of statistical terms routinely used in meta-analyses: an international survey among researchers. PLoS One. 2013;8(1):e47229. doi: 10.1371/journal.pone.0047229. Epub 2013 Jan 11. PMID: 23326299; PMCID: PMC3543405. | Wrong focus/not about inference |
| Otte WM, Vinkers CH, Habets PC, van IJzendoorn DGP, Tijdink JK (2022) Analysis of 567,758 randomized controlled trials published over 30 years reveals trends in phrases used to discuss results that do not reach statistical significance. PLoS Biol 20(2): e3001562. https://doi.org/10.1371/journal.pbio.3001562 | Wrong outcome |
| Sawada T, Huang L and Koryakov OY (2022) Some misunderstandings in psychology about confidence intervals. *Front. Psychol.* 13:948423. doi: 10.3389/fpsyg.2022.948423 | Wrong population |
| Stalvey, H., Burns-Childers, A., Chamberlain, D., Kemp, A., Meadows, L., & Vidakovic, D. (2019). Students' Understanding of the Concepts Involved in One-Sample Hypothesis Testing. *Journal of Mathematical Behavior, 53*(). https://doi.org/10.1016/j.jmathb.2018.03.011 | Wrong focus/not about inference |
| Wilkerson, M., & Olson, M. R. (1997). Misconceptions About Sample Size, Statistical Significance, and Treatment Effect. *The Journal of Psychology*, *131*(6), 627–631. https://doi.org/10.1080/00223989709603844 | Wrong outcome |
| Zuckerman, M., Hodgins, H. S., Zuckerman, A., & Rosenthal, R. (1993). Contemporary Issues in the Analysis of Data: A Survey of 551 Psychologists. *Psychological Science*, *4*(1), 49-53. https://doi.org/10.1111/j.1467-9280.1993.tb00556.x (Original work published 1993) | Wrong outcome |

# Assessment of Risk of bias in included studies

Below is a visualization, performed with robvis visualization tool (1) of the assessment of Risk of Bias in the included studies. Assessments were performed using the Risk of Bias Instrument for Cross-Sectional Surveys of Attitudes and Practices, developed by the CLARITY Group at McMaster University (2). The instrument assesses biases across five domains:

**Domain, D1:** is the source population representative of the population of interest?

**Domain, D2:** is the response rate adequate?

**Domain, D3:** is there little missing data?

**Domain, D4:** is the survey clinically sensible?

**Domain, D5:** if there is evidence of the reliability and validity of the survey instrument?

Assessments were assessed with the response alternatives:

- definitely/probably yes	(green +)
- definitely/probably no	(red **X** )
- no (no) information/not applicable	(blue **?**)

**References**

1) McGuinness, LA, Higgins, JPT. Risk-of-bias VISualization (robvis): An R package and Shiny web app for visualizing risk-of-bias assessments. Res Syn Meth. 2020; 1- 7.
2) CLARITY Group at McMaster University. 2021. Risk of Bias Instrument for Cross-Sectional Surveys of Attitudes and Practices. https://www.distillersr.com/wp-content/uploads/2021/03/Risk-of-Bias-Instrument-for-Cross-Sectional-Surveys-of-Attitudes-and-Practices-DistillerSR.pdf

# Summary of misconceptions and fallacies related to inferences from p-values, statistically significant findings, and other aspects of NHST

Note that there is no consistent terminology in the literature; the same mistakes and fallacies may appear under different names.

| **Misconceptions and fallacies regarding inferences of p-values and statistically significant findings or other aspects of NHST** | | |
| --- | --- | --- |
| **Name** | **Description** | **Why it is a fallacy** |
| Inverse probability fallacy (in relation to the null hypothesis) | The mistaken belief that the p-value represents the probability that the null hypothesis is true or false. | The p-value measures how likely the observed data (or more extreme data) are assuming the null hypothesis is true. Therefore, it is not a probability that applies to the truth of any hypothesis itself. |
| Inverse probability fallacy (in relation to the alternative hypothesis) | The mistaken belief that 1 − p (or 1 − α) represents the probability that the alternative hypothesis is true. | The p-value is not defined in relation to the alternative hypothesis. Since p-values are calculated assuming the null hypothesis is true, they cannot indicate the probability that the alternative hypothesis is correct. |
| Fallacy of proof | This is the misconception that a small p-value or statistically significant result (e.g., p < 0.05) proves the null hypothesis is false or the alternative hypothesis is true. | Statistical tests use sample data and can only provide evidence - not proof - about populations. A small p-value suggests the data are unlikely under the null but doesn't confirm the null is false or the alternative is true. |
| P-value misinterpretation as probability of results being due to chance fallacy | The mistaken belief that a p-value or statistically significant finding directly measures the probability that the results are due to chance. | The p-value is calculated assuming the null hypothesis is true - that any difference observed is due to random chance. Because of this assumption, it cannot indicate the probability that the null hypothesis or the assumption itself is true or false. |
| Acceptance fallacy | The mistaken belief that a non-significant result (p > 0.05) proves that the null hypothesis is true. | A non-significant result only indicates that there is insufficient evidence to reject the null hypothesis; it does not prove that the null hypothesis is true. |
| Error probability fallacy | The mistaken belief that a p-value (e.g., p = 0.04) means there is only a 4% probability that the conclusion is wrong and a 96% probability that it is correct. | The p-value is not the probability that a conclusion is correct or incorrect; it only reflects how unusual the observed data are under the assumption that the null hypothesis is true. It is not possible to determine the risk of error from a single test without additional external information. |
| Magnitude fallacy | The mistaken belief that a smaller p-value indicates a larger or more important effect. | The p-value reflects the strength of evidence against the null hypothesis but does not directly indicate the magnitude or practical importance of an effect. Effect size measures are needed to assess magnitude. |
| Practical significance fallacy | The mistaken belief that statistical significance automatically implies that the result is clinically or practically important. | Statistical significance does not guarantee practical importance. A result can be statistically significant but have minimal real-world relevance, particularly in large samples where small, trivial effects can achieve significance. |
| Replication fallacy | The mistaken belief that a p-value or statistically significant finding implies that the results will likely replicate in future studies. | The p-value does not reflect the likelihood of replication. Replication success depends on factors such as effect size, study power, and methodological consistency, not merely statistical significance. |
| Dichotomous thinking fallacy (or cliffhanger fallacy) | The inclination to believe that results can be cleanly categorized as "proved" or "disproved" based solely on whether the p-value crosses an arbitrary threshold (e.g., 0.05). | This fallacy ignores that the p-value represents a continuous measure of evidence. Dividing results into "significant" or "not significant" categories based on a fixed threshold oversimplifies the uncertainty and context inherent in statistical inference. |
| Difference in significance fallacy | The mistaken belief that if a result is statistically significant in one group (e.g., p < 0.05) and not significant in another group (e.g., p > 0.05), then the groups differ significantly from each other. | This is a fallacy because statistical significance is not a direct measure of effect size, and differences in p-values do not imply differences in effects. To determine whether groups differ significantly, a direct statistical comparison (e.g., interaction test) is required |

# Summary of misconceptions and fallacies related to inferences from confidence intervals and comparisons

Note that there is no consistent terminology in the literature; the same mistakes and fallacies may appear under different names.

| **Misconceptions and fallacies regarding inferences of confidence intervals and comparisons** | | |
| --- | --- | --- |
| **Name** | **Description** | **Why it is a fallacy** |
| Probability fallacy (or inverse probability fallacy) | The mistaken belief that a 95% confidence interval means there is a 95% probability that the true parameter lies within the observed interval. | Once calculated, a confidence interval either contains the true parameter or it does not (probability = 0 or 1). The 95% refers to the long-run frequency: if the procedure were repeated many times, 95% of intervals would contain the true parameter. |
| Precision fallacy | The mistaken belief that a confidence interval directly measures the precision or accuracy of a single estimate. | A confidence interval reflects the uncertainty in the estimation procedure, not the precision of a particular estimate. Precision relates to variability, while the interval represents the range of plausible values for the parameter. |
| Certainty fallacy | The mistaken belief that values outside the confidence interval are impossible or have been "ruled out" with certainty. | Values outside the confidence interval are simply less compatible with the observed data, but they are not impossible. The interval represents a range of reasonably plausible values, not absolute boundaries of possibility. |
| Replication fallacy | The mistaken belief that 95% of future confidence intervals will overlap with the current confidence interval. | The confidence level refers to the proportion of intervals that will contain the true parameter, not the proportion that will overlap with any particular interval. Future intervals depend on new data and may or may not overlap with the current interval. |
| Null hypothesis equivalence fallacy | The mistaken belief that if a confidence interval contains the null value (e.g., 0 for a difference), this proves the null hypothesis is true. | A confidence interval containing the null value indicates that the null hypothesis is plausible given the data, but it does not prove the null hypothesis is true. The interval represents a range of plausible values, including but not limited to the null. |
| Confidence level misinterpretation fallacy | The mistaken belief that choosing a higher confidence level (e.g., 99% vs. 95%) always provides "better" or more useful information. | Higher confidence levels produce wider intervals, which may be less informative for practical decision-making. The choice of confidence level should reflect the balance between precision (narrower intervals) and confidence (higher probability of containing the parameter). |
| Confidence interval overlap fallacy | The mistaken belief that two estimates are statistically significantly different only if their confidence intervals do not overlap - and that if they do overlap, the difference is not statistically significant. | Confidence interval overlap does not reliably indicate statistical significance. Only under specific conditions does non-overlap suggest significance, and the correct method is to test the difference directly, not compare separate intervals  The misconception that two estimates are statistically significantly different only if their confidence intervals do not overlap - and that if they do overlap, the difference is not statistically significant. |
| Descriptive interval fallacy | The mistaken belief that a confidence interval describes the distribution of individual data points (e.g., the range of individual scores) or represents only the  or sample, such as the sample mean or range. | Confidence intervals are inferential estimates of a population parameter, not descriptive measures of sample data |
