# Supplementary material for: Inferences of p-values, statistically significant results, and confidence intervals – A systematic review of empirical studies
Source: PLoS One. 2026 Nov 11;21(11):e0362071. doi: 10.1371/journal.pone.0362071 (PMC13754157; doi:10.1371/journal.pone.0362071)
Supplement: Supplementary file 3 [file pone.0362071.s002.docx]

Supplement 2

Tabulated studies, in alphabetical order by first author’s surname

[Studies about inferences of results from null hypothesis significance testing	2](#_Toc209860190)

[Studies about inferences of results from confidence intervals (and results from null hypothesis significance testing)	66](#_Toc209860191)

## Studies about inferences of results from null hypothesis significance testing

| **Author**  **Year**  **Country** | [Badenes-Ribera](https://www.frontiersin.org/people/u/191756) et al.  2015  Spain |
| --- | --- |
| **Study design**  **Purpose**  **Procedure/response rate**  **Population** | Online survey carried out addressing academic psychologists in Spain.  The purpose of the present study was to detect the statistical reasoning errors that academic psychologists make when presented with the results of a statistical inference test.  A total of 4066 potential participants, whose emails were found on university websites during 2013 and 2014, were contacted and invited to participate. Reminder to non-respondents after two weeks. Response rate 10.3% (n=418).  The sample was composed of 418 academic psychologists at different universities and belong to different research areas. The mean number of years of the professors at the University was 14.2 years (SD = 9.4); the study sample consisted of 51.4% women. |
| **Type of questionnaire**  **Questions about inference**  **Type of inference investigated** | Web questionnaire consisting of 10 questions.  Let’s suppose that a research article indicates a value of p = 0.001 in the results section (alpha = 0.05). Mark which of the following statements are true (T) or false (F).  Inverse probability 1-5, replication 6, effect size 7, clinical significance 8, assertiveness of being correct, 9-10 “correct inferences” according to authors. |
| **Results** | Authors claim that propositions 1-8 are fallacies (false) and that 9-10 are true.  Proportion of responses of incorrect inferences (in brackets) of total population:   1. The null hypothesis has been shown to be true (7.4%) 2. The null hypothesis has been shown to be false (58.6%) 3. The probability of the null hypothesis has been determined (p = 0.001), (58.4%) 4. The probability of the experimental hypothesis has been deduced (p = 0.001), (33.7%) 5. The probability that the null hypothesis is true, given the data obtained, is 0.01, (30.6%) 6. A later replication would have a probability of 0.999 (1-0.001) of being significant, 34.7%) 7. The value p = 0.001 directly confirms that the effect size was large. (13.2%) 8. Obtaining a statistically significant result indirectly implies that the effect detected is important. (35.2%) 9. The probability of the result of the statistical test is known, assuming that the null hypothesis is true. (Results presented as figure for subgroups) 10. Given that p = 0.001, the result obtained makes it possible to conclude that the differences are not due to chance (Results presented as figure for subgroups) |
| **Authors description of results/conclusion** | Our findings suggest that many academic psychologists do not know how to correctly interpret p values. The inverse probability fallacy presents the greatest comprehension problems. Methodology instructors also interpret the significance of the p-value erroneously. |
| **Methodological comments**  **Other comments** | Relevant population. Very poor response rate. Good face validity for outcome questions.  Statements 9 and 10 are claimed to be correct by the authors. However, Statement 9 is not actually a definition of a p-value, and Statement 10 is simply a variant of Statement 4—the misunderstanding that the existence of a true effect can be deduced from a small p-value. |

| **Author**  **Year**  **Country** | Badenes-Ribera et al.  2016  Spain |
| --- | --- |
| **Study design**    **Purpose**        **Procedure/response rate**        **Population** | E-mailed survey.    The purpose was to analyse what academic psychologists know about effect sizes, their CI:s and meta-analysis.      A total of 4463 potential participants whose emails were found on university websites of were contacted during 2013 and 2014. Reminder to non-respondents was sent after two weeks. Response rate 10.6% (n=472).      The sample is consisted of 472 academic psychologists. The mean number of years of the at the university was 13.6 years (SD = 9.3). Women 54.2%. |
| **Type of questionnaire**      **Questions about inference**    **Type of inference investigated** | Email survey with two sections where the second section involved questions about statistical terms, opinions about meta-analyses, if participants have “used” a meta-analytic study and questions about researchers’ behavior and understanding of various concepts.    Of all questions, one was about inference of “statistical significance”. The question was “In your opinion, obtaining a statistically significant result implies indirectly that the detected effect is important” with response alternatives: yes or no.    Whether statistical significance implies clinical significance |
| **Results** | Results were presented according to those who could a name of an effect statistic (n=323 could do so) and those who could not (n=149, could not)  In your opinion, obtaining a statistically significant result implies indirectly that the detected effect is important.  **No** (do not know a test statistic) 45.6% (do know a test statistic) 69.7%  **Yes** (do not know a test statistic) 54.4% (do know a test statistic) 30.3% |
| **Authors description of results/conclusion** | From discussion:  “It is noteworthy that academics who named an ES statistic … also make to a lesser extent the clinical or practical size fallacy where the statistical significance of the effect is related to its importance, although in both groups of academics, more than 30% of the subjects believe in that association. However, a statistically significant effect can be found, but will not have any clinical importance, and vice versa. |
| **Methodological comments**    **Other comments** |  |

| **Author**  **Year**  **Country** | [Badenes-Ribera](https://www.frontiersin.org/people/u/191756) et al.  2016  Spain (Chile and Italy) |
| --- | --- |
| **Study design**  **Purpose**  **Procedure/response rate**  **Population** | Online survey carried out addressing academic psychologists from Chile and Italy.  Purpose: analyzes if the most common misconceptions of p-values are made by academic psychologists from different geographic areas than the original research (i.e. Spain (Badenes-Ribera et al., 2015), Germany (Haller and Krauss, 2002) and United States of America (Oakes, 1986).  A convenience sample was recruited by contacting 2,321 academic psychologists in Chile and Italy. The initial sample comprised 194 participants; 30 did not answer the questions on p-value misconceptions and were excluded, leaving 164 participants for analysis (overall response rate 7.07%)  134 Italian (53.7% women, mean age 48.4 (SD=10.6) and 30 Chilean (50.0% women; mean age 44.5 (SD=9.2). The mean number of years spent in academia 13.3 years (SD 10.5) and 15.5 years (SD 8.7) in Italy and Chile, respectively. |
| **Type of questionnaire**  **Questions about inference**  **Type of inference investigated** | Web questionnaire consisting of 10 questions.  “Suppose that a research article indicates a value of p = 0.001 in the results section (α = 0.05). Select true statements.”  Inverse probability (1-5), replication (6), Effect size (7), Clinical significance (8), Assertiveness of being correct (9-10) |
| **Results** | Authors claim that propositions 1-8 are fallacies (false) and that 9-10 are true.  Proportion of responses of incorrect inferences (in brackets) of total population:   1. The null hypothesis has been shown to be true (3.7%) 2. The null hypothesis has been shown to be false (32.9%) 3. The probability of the null hypothesis has been determined (p = 0.001) (23.8%) 4. The probability of the experimental hypothesis has been deduced (p = 0.001) (12.2%) 5. The probability that the null hypothesis is true, given the data obtained, is 0.01 (13.4%) 6. A later replication would have a probability of 0.999 (1-0.001) of being significant (12.2%) 7. The value p = 0.001 directly confirms that the effect size was large. (4.9%) 8. Obtaining a statistically significant result indirectly implies that the effect detected is important. (8.5%) 9. The probability of the result of the statistical test is known, assuming that the null hypothesis is true. (Results presented as figure for subgroups) 10. Given that p = 0.001, the result obtained makes it possible to conclude that the differences are not due to chance. (Results presented as figure for subgroups) |
| **Authors result/conclusion** | Our findings are consistent with previous research and suggest that some participants do not know how to correctly interpret p-values. The inverse probability fallacy presents the greatest comprehension problems, followed by the replication fallacy. |
| **Methodological comments**  **Other comments** | Relevant population. Fair response rate but using convenient sampling. Good face validity for outcome questions, which had been previously used.  Statement 9 and 10 are claimed to be correct by authors, but statement 9 is not a definition of a p-value and statement 10 is a variant of statement 4, the misunderstanding that the existence of a true effect can be deduced from a small p-value. |

| **Author**  **Year**  **Country** | [Badenes-Ribera](https://www.frontiersin.org/people/u/191756) et al.  2018  Spain |
| --- | --- |
| **Study design**  **Purpose**  **Procedure/response rate**  **Population** | Online survey carried out addressing academic psychologists from Italy.  Purpose: assess self-perceived knowledge about different statistical methods and concepts and with a focus on effects size. One question related to inferences of statistically significant results.  The e-mail addresses of Italian academic psychologists were found by consulting the websites of Italian universities, resulting in 1,824 potential participants.  Potential participants were invited to complete a survey using a computer assisted web interview system. A follow-up message was sent 2 weeks later to non-respondents. 159 responded response rate 8.72%  159 Italian psychologists, 54.1% women, mean age 47.6 (SD=10.5). The mean number of years spent in academia 12.9 years (SD 10.2) of whom 84.9% had been reviewers for scientific journals past year. |
| **Type of questionnaire**  **Questions about inference**  **Type of inference investigated** | Web questionnaire consisting of 17 questions, one of relevance to statistical inference.  ”In your opinion, obtaining a statistically significant result indirectly implies that the detected effect is important” yes/no  Statistically significance implies clinical significance. |
| **Results** | Results stratified to responses if respondents could name an effect size statistic or not (I don’t know vs. yes (specify its name).  Those not knowing effect size (n=88): yes 10.2%  Those specifying known effect size statistic: yes 8.5% |
| **Authors result/conclusion** | As in the original research, the results showed that, although the use of effect  size estimates is becoming generalized, an under-reporting of CIs for ES persists. The  most frequent ES statistics mentioned were Cohen’s d and R2/!  2, which can have  outliers or show non-normality or violate statistical assumptions. In addition, academics  showed poor knowledge about meta-analytic displays (e.g., forest plot and funnel plot)  and quality checklists for studies. Finally, academics with higher-level knowledge about  ES statistics seem to have a profile closer to good statistical practices. |
| **Methodological comments**  **Other comments** | Relevant population. Very low response rate. Only one question relevant to inferences. |

| **Author**  **Year**  **Country** | Beauchamp  1964  US |
| --- | --- |
| **Study design**  **Purpose**  **Procedure/response rate**  **Population** | Cross sectional questionnaire.  Replicate the study of Rosenthal and Gatio (1963)  Eleven graduate students and nine PhDs were asked to express their “degree of belief” in research findings of associated p-levels and sample sizes of 100 and 10.  See above. |
| **Type of questionnaire**  **Questions about inference**  **Type of inference investigated** | Written questionnaire.  Subjects completed a questionnaire requesting them to rate their degree of belief or confidence in a variety of p levels: once assuming an n of 10, and then assuming an n of 100. The possible ratings they were to assign to each p-level were as follows:  5: extreme confidence or belief  4: great confidence or belief  3: moderate confidence or belief  2: mild confidence or belief  1: minimal confidence or belief  0: complete absence of confidence or belief |
| **Results/Outcomes** | Effects due to sample size and p-levels were significant in both studies.  No cliff hanger effect was found. |
| **Authors result/conclusion** | The results generally confirmed those of Rosenthal and Gatio. |
| **Methodological comments**  **Other comments** |  |

| **Author**  **Year**  **Country** | Bezzina et al.  2014  Malta and United Kingdom |
| --- | --- |
| **Study design**  **Purpose**  **Procedure/response rate**  **Population** | Online survey.  Purpose: to study if statistical misconceptions pervasive among academics with a special interest in business research methods?  The target population consisted of academics who are members of the Research Methodology Special Interest Groups of either the British Academy of Management or The European Academy of Management or have attended the European Conference on Research Methodology at least once in the past three year, estimated total of 679 potential respondents after accounting for multiple list membership. Emailed invitation. Response rate; 11.8% returned complete responses.  80 respondents competed the questionnaire. Mostly academics with document interest in research methodology: male (51.2%), having doctoral degree (70.0%), from United Kingdom (53.8%). |
| **Type of questionnaire**  **Questions about inference**  **Type of inference investigated** | The main questionnaire consisted of 30 randomly ordered categorical statements representing statistical mis/conceptions. Respondents were requested to tick one from ‘agree’, ‘disagree’ or ‘don’t know’.  Two questions about inferential statistics, one about p-values and one about inferring clinical significance of statistically significant results.  Inverse probability, Clinical significance. |
| **Results** | False agreements to statements:  “The p-value is the probability that the null hypothesis H_0_ is true.” 32% of respondents marked “Agree”  “Statistically significant results signify strong relationships between variables or big differences between comparison groups” 42 % of respondents marked “Agree” |
| **Authors result/conclusion** | General: The findings of this study suggest both a lack of knowledge and a high pervasiveness of statistical misconceptions among academics with a special interest in business research methods. |
| **Methodological comments**  **Other comments** | Relevant population. Low response rate. Only two question relevant to inferences with straightforward face validity |

| **Author**  **Year**  **Country** | Bookstaver et al.  2012  USA |
| --- | --- |
| **Study design**  **Purpose**  **Procedure/response rate**  **Population** | Cross sectional study.  To evaluate the level of understanding and perception of biostatistics concepts among pharmacy residents.  A 27-item survey, including 10 knowledge-based questions, was distributed to American Society of Health-System Pharmacists–accredited residency programs residency programs. About 1400 received the survey and total of 214 residents responded to the online survey assessment, and a subset of respondents (n = 166) answered 1 or more of the biostatistics knowledge questions. Overall response rate about 12%. One question was about interpreting a non-significant finding (p>.05).  Of the 166 responders, 73.5 were women and 86.6 reported having previous training in biostatistics. |
| **Type of questionnaire**  **Questions about inference**  **Type of inference investigated** | Emailed survey.  The question about interpreting a non-significant finding is not presented. It is likely the same as used in Windish et al (see tabulation)  NHST |
| **Results** | Proportion of respondents giving a correct response, according to authors:  59.6% (95% CI 51.97 to 67.29) |
| **Authors result/conclusion** | Overall, pharmacy residents’ perception and understanding of biostatistics were poor in this assessment, which correlates with previous reports. |
| **Methodological comments**  **Other comments** |  |

| **Author**  **Year**  **Country** | Castro Sotos et al.  2009  Spain |
| --- | --- |
| **Study design**  **Purpose**  **Procedure/response rate**  **Population** | Cross sectional questionnaire  To investigate misconceptions about hypothesis tests by (1), and (2) also investigate the confidence that students have in their misconceptions.  An instrument with multiple-choice items was developed to address three misconceptions concerning hypothesis tests. Students were asked to select the correct answer for each item; however, they often picked more than one response. They were, furthermore, asked to address on 0-10 graded scale, their confidence in their answers. The response rate is unclear.  144 university undergraduates following introductory statistics courses were invited. |
| **Type of questionnaire**  **Questions about inference**  **Type of inference investigated** | Written questionnaire.  There was one question about the p-value:  Researchers have registered the values of a quantitative value for two groups of individuals and carried out a test of the hypothesis that there is no difference between the groups concerning that variable. The hypothesis test (sic!) results in a p-value of 0.01. Select the conclusion that you consider most correct. There were seven multiple choice options.  And there was one question about a statistically significant finding with six multiple choice options. |
| **Results/Outcomes** | Percentages of selected responses for the definition of hypothesis test  Option “A hypothesis test is… Percentage  H 1  …a proof of H0 (or) or its falseness” 20%  ….a proof of Ha (or) or its falseness” 1%  H 2  ….a proof of the probability or improbability of H0” 19%  …a proof of the probability or improbability of Ha” 3%  Correct  …an evaluation of the evidence in favor or against H0 in the data” 62%  Confusing H0 and Ha  …an evaluation of the evidence in favor or against Ha in the data” 6%  Completely correct answer: Selecting the correct option only 56%  Mean confidence on a 10-degree scale: 6.21 (SD 2.58)  Percentages of selected responses for the definition of the p-value  Option "Given a p-value of 0.01… Percentage  p3 … the probability of the null hypothesis is 0.01" 12%  p1 … the probability of the null hypothesis assuming the same or more extreme data is 0.01"  9%  Correct … the probability of obtaining the same or more extreme data assuming the null hypothesis is true" 52%  p2 … the probability of obtaining the same or more extreme data is 0.01" 7%  p4 … the probability of making an error when rejecting the null hypothesis is 0.01" 16%  p5 …the difference between the groups is big" 8%  p6 …the difference between the groups is small" 10%  Completely correct answer: Selecting the correct option only 46%  Mean confidence on a 10-degree scale 5.66 (SD 2.96) |
| **Authors result/conclusion** | From abstract:  A considerable number of participants seemed to hold misconceptions and lower levels of concept-specific self-perceived efficacy were found to be related to misconceptions more than to the correct answers. On average, students selected significantly lower levels of confidence for the question addressing the definition of the significance level than for the other two items. |
| **Methodological comments**  **Other comments** | It was common that more than one answer was picked.  The authors seem to confuse hypothesis test with significance test. |

| **Author**  **Year**  **Country** | Coulson et al.  2010  Australia |
| --- | --- |
| **Study design**  **Purpose**  **Procedure/response rate**  **Population** | Emailed survey, two experiments reported one relevant for confidence intervals and one of relevance for interpreting NHST results. Respondents were presented results in one of four formats: as CI figure, CI text, NHST figure or NHST test and 2) ratings of agreement of results.  Comparison of interpretation of results presented using null hypothesis significance testing (NHST), or confidence intervals (CIs).  2 322 emails were sent (190 emails (8.2%) bounced) to researchers who had published in psychology, behavioral neuroscience, and medical journals were asked by email to interpret the results of our two fictitious studies. Response rate 15.5% of 2,132 assumed received.  N=330 of researchers published in scientific journals, see above. |
| **Type of questionnaire**  **Questions about inference**  **Type of inference investigated** | Emailed survey. We asked respondents to interpret two similar studies presenting results in different ways. One gave a statistically significant (p = 0.02), and the other a non-significant (p = 0.22) result in the same direction. Results were shown as CI figure, CI in text, NHST figure or NHST text. The data were the same in every case.  Authors were asked, first, to answer in their own words “what do you feel is the main conclusion suggested by these studies?” and were then asked to rate agreement from 1 (strongly disagree) to 7 (strongly agree) about the results in the two studies 1) being consistent, 2) strong evidence of one being more effective and 3) conflict about effectiveness.  Comparison of inference of CI and NHST in terms of consistency, effect and conflict about effect. |
| **Results** | Several comparisons of assessments of “consistency” and “effectiveness” scores are presented in figures for different subgroups and for vignettes for NHST and CI and combined.  Readers are referred to actual article för results. |
| **Authors result/conclusion** | Text responses to the initial question were very diverse. Many gave evidence of misconception, especially about p values or NHST.  General: Responses from 330 authors varied greatly, but interpretation was generally poor, whether results were presented as CIs or using NHST. Confidence intervals permit, but do not guarantee, better inference than statistical significance testing |
| **Methodological comments**  **Other comments** | Relevant population. Low response rate. Complex assessments. |

| **Author**  **Year**  **Country** | Cumming et al.  2004  Australia |
| --- | --- |
| **Study design**  **Purpose**  **Procedure/response rate**  **Population** | Internet based investigation on researchers’ understanding of Cis, standards error bars, and replication.  To seek to contribute to the currently spares knowledge of statistical cognition of CIs by investigating researchers understating of error bars in relation to replication, where the researcher’s belief in replication was assessed as the probability that a replicated sample mean will fall within the interval of a given original CI.  3848 emails were sent to researchers who had published in psychology, behavioral neuroscience, and medical journals were asked by email to interpret the results of our two fictitious studies. Response rate who completed the task 8.7%.  N=263 of researchers published in scientific journals, see above. |
| **Type of questionnaire**  **Questions about inference**  **Type of inference investigated** | Emailed survey to experimental website were visitors saw figures with mean and CI or SE error bars.  Authors were asked to plot ten lines that would be plausible sets of means for further ten independent samples taken from the same population. There are different websites and questions presenting horizontal lines as Cis and error bars, respectively. Participants were then asked to plot ten lines that could plausibly be a set of means for ten further independent samples of the same size taken from the same populations.  Anticipated replication when viewing means with CI and error bars. |
| **Results** | In the CI condition, 54.5% of respondents included all 10 means within the bars; 78.4% placed 9 or 10 within the bars. For the standards error condition, 16.3% included all 10 within the original bars, and 69.0% included from 6 to 10. The CI average was 9.18 means within bars and the standard error averaged 6.67. In other words, a large proportion of respondents are generally consistent with what we call the confidence level misconception, which is the belief that about C% of replication means will fall within C% of CIs. |
| **Authors result/conclusion** | A 95% CI will on average capture 83.4% of future replications means. A majority of respondents, however, held the confidence level misconception that 95% of a CI will on average capture 95% of replication means. |
| **Methodological comments**  **Other comments** | Relevant population. Very low response rate. Complex assessment. |

| **Author**  **Year**  **Country** | Falk et al.  1995  Israel |
| --- | --- |
| **Study design**  **Purpose**  **Procedure/response rate**  **Population** | Survey, most likely written.  The purpose was to test whether the misinterpretation of statistical significance disappears when students are warned, and the mistake is pointed out to them.  53 students of psychology at the Hebrew University of Jerusalem were asked to answer one question. The student had two courses in statistics and probability plus one course of experimental psychology. Response rate not reported.  53 students of psychology at the Hebrew University of Jerusalem were asked to answer one question. The student had two courses in statistics and probability plus one course of experimental psychology. They had read Bakan’s (1966) paper about inferential mistakes. |
| **Type of questionnaire**  **Questions about inference**  **Type of inference investigated** | Written  Participant were told that at test of significance was conducted and the result turned out significant for the predetermined level. They were asked the meaning of such results. Five statements were presented, and subjects were allowed to mark more than one answer as true. |
| **Results** | All participants marked only one option despite being able to mark more.  Statements endorsed as true (%). The correct answer is number 5.   1. We proved that H_0_ is not true (3.8%) 2. We proved that H_1_ is true. (0%) 3. We showed that H_0_ is improbable (79.2%) 4. We showed that H_1_ is probable (3.8%) 5. None of the answers 1-4 are correct (13.2%) |
| **Authors result/conclusion** | From text:  Answer 5, the correct one, was selected by seven of the subjects. The other options chosen (1,3 and 4) are all variants of the illusion of attaining improbably. The percentage endorsing the misinterpretations (86.8%) is fairly close the results of the inquiry among academic psychologists by Oakes (1986). Altogether, misinterpreting a significant result as conveying the probability of the null hypothesis is prevalent and robust int eh face of variation in problem presentation and in subjects’ level of sophistication. |
| **Methodological comments**  **Other comments** | Relevant population. Poorly described investigation procedure. Previously used outcome questions. |

| **Author**  **Year**  **Country** | Ferreira et al.  2020  Portugal |
| --- | --- |
| **Study design**  **Purpose**  **Procedure/response rate**  **Population** | Online questionnaire addressing common misinterpretations of the p-value.  In this questionnaire, a question was asked to ascertain the respondents’ perception of their knowledge of what a statistically significant p-value meant.  A questionnaire was made available online during January 2019 and April 2019 and was spread through social media. People were asked to answer and share the questionnaire.  Persons were asked if they knew how to interpret information provided by significance (p-value) of randomized trial and only those who answered “yes” could answer the second part.  Response rate could not be calculated but 31.5% (n=153) stated that they think they know who to interpret information provided by the p-value.  The population consisted of 153 responding yes and who answered the inference question (61.7% physicians, 17.6% nurses and 17.5% of others). |
| **Type of questionnaire**  **Questions about inference**  **Type of inference investigated** | A questionnaire was made available online during January 2019 and April 2019 and was spread through social media. People were asked to answer and share the questionnaire.  Persons were asked if they knew how to interpret information provided by significance (p-value) of randomized trial and only those who answered “yes” could answer the second part.  Consider a specific treatment. You compare the control, and experimental groups mean using a t test for independent samples, having obtained a statistically significant result (t = 2.7, df = 38, p = 0.01). Based on this information, mark each of the following statements as being true or false:   A. You have the probability of the null hypothesis to be true. B. You have fully proved your experimental hypothesis (that there is a difference between population means). C. You can deduce the probability that the experimental hypothesis is true. D. You have a reliable experimental result in the sense that, if hypothetically, the experiment was repeated a large number of times, you would obtain a significant effect on 99% of the occasions.   Anticipated replication when viewing means with CI and error bars. |
| **Results** | A. You have the probability of the null hypothesis to be true. “True” physicians (57.9%), nurses (34%) and other (36.4%) B. You have fully proved your experimental hypothesis (that there is a difference between population means). “True” physicians (58.9%), nurses (84.0%), other (75.8%) C. You can deduce the probability that the experimental hypothesis is true. “True” physicians (76.8%), nurses (68.0%) and other (75.8%) D. You have a reliable experimental result in the sense that, if hypothetically, the experiment was repeated a large number of times, you would obtain a significant effect on 99% of the occasions. “True” physicians (75.8 %), nurses (84.0%) and other (84.8%)   *Note, statements A, B, C and D are all false.* |
| **Authors result/conclusion** | The outcomes show that misinterpretations of what the p-value means are still prevalent in health professionals. |
| **Methodological comments**  **Other comments** | Relevant population. High face validity of inference questions.  Replication of previously used outcome question. |

| **Author**  **Year**  **Country** | Ferill et al  1999  USA |
| --- | --- |
| **Study design**  **Purpose**  **Procedure/response rate**  **Population** | Written multiple choice survey, mailed by post.  To determine if pharmacists have the basic statistical knowledge believed to be required to evaluate medical and pharmacy studies.  A pilot study was conducted after which a survey was developed and sent to 2500 randomly selected pharmacists and students was obtained from the reader’s list of U.S. Pharmacist. The survey consisted of multiple-choice questions and was designed to take less than five minutes to complete. A response of “I don’t know” was included as a selection. Answers from 707 responders were usable making the response rate about 28%. Response rates to question A, B and C below were 75.2%, 43.3% and 33.4%.  Of responders 83.3% hade a bachelor’s in pharmacy. More than a third of responders had graduated over 21 years ago. The majority of responders had a community pharmacy practice site. |
| **Type of questionnaire**  **Questions about inference**  **Type of inference investigated** | Written  The questionnaire consisted of 10 questions, two were about interpreting a statistically significant finding and one was about the meaning of a confidence interval. See below.  Questions about statical significance:   A) If statistical significance has been demonstrated, the reader can be assured of the clinical significance of the findings of a study. a) True b) False (correct) c) I don’t know B) In studies where the level of significance is not stated, a P<0.05 reported with the data implies that: a) There is a <5 percent probability of stating that there is a difference when one does not exist. b) A statistically significant difference was found between groups tested. c) A clinically significant difference was found based on the p value. d) A and B (correct according to authors) e) I don’t know   The question about confidence interval was phrased:   C) Which of the following are characteristics of confidence intervals: a) Confidence intervals provide an estimate of the range where the true mean value in the affected population is likely to reside. b) Are abbreviated as CI or C.I. c) The most commonly used confidence interval is 95 percent. d) All of the above (correct according to authors) e) I don’t know |
| **Results** | Proportion answering correct and incorrect   A) Correct 50.8, incorrect 32.5% B) Correct 13.0% incorrect 69.9% C) Correct 22.9%, incorrect 31.4% |
| **Authors result/conclusion** | From discussion: From our data, it appears that pharmacists do not have adequate basic knowledge of statistics to adequately analyze the appropriateness of the use of statistics in the methods and results sections of research articles. |
| **Methodological comments**  **Other comments** | Response alternative a for question C about CI:s is not correct according to frequentist theory as the parameter is assumed to be fixed. |

| **Author**  **Year**  **Country** | Freeman  1993  Likely United Kingdom |
| --- | --- |
| **Study design**  **Purpose**  **Procedure/response rate**  **Population** | Short written test, returned by post.  Test the role of p-values in analyzing trial results.  Procedure is unclear. Author states the response rate was about 50%.  397 participants referred to as doctors, dentists and medical students. It is stated that some respondents had recently had a short course in statistical methods. |
| **Type of questionnaire**  **Questions about inference**  **Type of inference investigated** | Written  Participant was instructed in I vignette that “A controlled trial of a new treatment led to the conclusion that it is significantly better than placebo (p<0.05). Respondents where then asked to choose 1 preferred answer out of 4, se below.  Probability statements. |
| **Results** | Statements with proportions (%) of respondents preferring it.   1) It has been proved that treatment is better than placebo (15%) 2) If the treatment is not effective, there is less than a 5 per cent chance of obtain such results (19%) 3) The observed effect of the treatment is so large that there is less than a 5 per cent chance that the treatment is no better than placebo. (52%) 4) I do not really know what a p-value is and do not want to guess. (15%)   Response 2 is the most correct one. |
| **Authors result/conclusion** | Author discusses the various and wide spread of misinterpretations of p-values.  First conclusion point: “A p-value is at best a very crude measure of the weight of evidence against a hypothesis. If you must use one, quote it to only two significant figures at most (only the first has any real importance)” |
| **Methodological comments**  **Other comments** | Very poorly described investigation procedure. Unclear recruitment procedure, poor description of study sample. Reasonable outcome question. |

| **Author**  **Year**  **Country** | Haller et al.  2002  Germany |
| --- | --- |
| **Study design**  **Purpose**  **Procedure/response rate**  **Population** | Written questionnaire.  Purpose: investigate inferences of null hypothesis significance testing.  Participants at psychology departments were recruited at 6 German universities. Response rate not reported.    The group consisted of methodology instructors (teachers of methods and statistics, n=30), scientific psychologist (professors and other staff not involved in teaching of statistics, n=39, and psychology students (n=44). |
| **Type of questionnaire**  **Questions about inference**  **Type of inference investigated** | Web questionnaire consisting of 10 questions.  Respondents were presented with the results of an experiment and were asked to mark the six statements (under results) as true or false. The experiment was described as: “Suppose you have a treatment that you suspect may alter performance on a certain task. You compare the means of your control and experimental groups (say 20 subjects in each sample). Further, suppose you use a simple independent means t-test and your result is (t = 2.7, d.f. = 18, p = 0.01).   1. You have absolutely disproved the null hypothesis (that is, there is no difference between the population means). 2. You have found the probability of the null hypothesis being true. 3. You have absolutely proved your experimental hypothesis (that there is a difference between the population means). 4. You can deduce the probability of the experimental hypothesis being true. 5. You know, if you decide to reject the null hypothesis, the probability that you are making the wrong decision. 6. You have a reliable experimental finding in the sense that if, hypothetically, the experiment were repeated a great number of times, you would obtain a significant result on 99% of occasions.   Absolute proof, Inverse probability, replication, assertiveness of being correct |
| **Results** | Proportion of responses with incorrect inferences  Methodology instructors scientific phycologists Psychology students  1 10% 15% 34%  2 17% 26% 32%  3 10% 13% 20%  4 33% 33% 59%  5 73% 67% 68%  6 37% 49% 41% |
| **Authors result/conclusion** | We presented six common misinterpretations to psychologists who work in German universities and found out that they are still surprisingly widespread – even among instructors who teach statistics to psychology students. |
| **Methodological comments**  **Other comments** | Relevant population. Fair response rate, but using convenient sampling. Good face validity for outcome questions, which had been previously used. |

| **Author**  **Year**  **Country** | [G](https://www.frontiersin.org/people/u/191756)ordon, Howard.  2001  US |
| --- | --- |
| **Study design**  **Purpose**  **Procedure/response rate**  **Population** | Mail and internet survey  The purpose of this study was to identify American Vocational Education Research Association Members’ (AVERA) perceptions of statistical significance tests  A random sample of the current AVERA members was selected and the psychometric Group Instrument (by Mittag, 1999) was used to determine participants perceptions using 1-5 response rate Likert scales. (1 disagree, 2 somewhat disagree, 3 neutral, 4 somewhat agree and 5 agree)  113 participants, 67% males, 95% had earned doctoral degree, 60% hade over 15 years of experience of educational research and 82.5% worked at a university. |
| **Type of questionnaire**  **Questions about inference**  **Type of inference investigated** | Mailed and online questionnaire containing a total of 29 Likert type items with a 1-5 response scale (1=(1= disagree, 2 = somewhat disagree, 3 = neutral, 4 = somewhat agree, and5 = agree).  Five of the 29 items relate to inferences of statistical test results and were reported in terms av means (SD) of the Likert scale results.  Agreement with statements. |
| **Results** | Mean (SD) to questions related to inference.   - All that significance means is that the researcher rejected the null hypothesis 3.02 (1.44) - Finding that p<.05 is one indication that the results are important 2.80 (1.41) - Studies with non-significant results can still be very important. 1.45 (1.19) - Smaller and smaller values for the calculated p indicate that the results are more likely to be replicated in future research. 2.82 (1.33) - Significance tests evaluate the probability that the results for the sample are the same in the population. 2.22 (1.09) |
| **Authors description of results/conclusion** | 1) From authors discussion: It appears that AVERA members who were most comfortable with and interested in statistical issues (quantitative methods) may have been most likely to respond to the survey. AVERA members' general views regarding statistical testing appeared to be consistent with previous research |
| **Methodological comments**  **Other comments** | 2) The questions assess perceptions for items that does not have a clear answer and there is no reference population. Thus, the results are difficult to interpret. Results are summed up as means based on ordinal data. 3) |

| **Author**  **Year**  **Country** | Kalinowski et al.  2008  Australia |
| --- | --- |
| **Study design**  **Purpose**  **Procedure/response rate**  **Population** | Cross sectional survey  To test the effect the effectiveness of two teaching interventions aimed at reducing the inverse probability fallacy. A pre-intervention test was performed to assess this misunderstanding in the study population as well as a post intervention test.  Survey and intervention were given students enrolled in the Bachelor of Behavioural Science degree at La Trobe University, Australia.  A total of 120 final year undergraduate students, which in the intervention were exposed to either teachings about Bayes theorem to contrast Bayesian posterior probabilities to p-values (B-group, n=56), or to comparison group (MT, n=64) |
| **Type of questionnaire**  **Questions about inference**  **Type of inference investigated** | Survey.  Variations of six statements of typical misinterpretations of p values were presented in the survey and participants were asked to mark their level of agreement with each statement on a 7-point Likert scale, which in some analyses were collapsed dichotomous version of this scale.  Example of statement: “Sara explains that the probability of the null hypothesis being true is 5% or less” denoting the idea that the results from an NHST denotes the probability that H0 is found.  Inferences of NHST results as absolute definite proof, odd s against chance fallacy, replication fallacy, inverse probability fallacy, inverse probability fallacy. |
| **Results** | Proportion of endorsements (pre intervention) of false statements  B-group MT-group   1. H0 is absolutely disproved 70% 53% 2. Probability of H0 is found 68% 61% 3. p = probability that H1 can be deduced 70% 72% 4. p = probability of replication 63% 52% 5. p = probability of the results being due to chance 68% 69% 6. p = probability of H0 given data. 77% 88% 7. All misconceptions correctly identified 7% 0%   Results from post-intervention not shown. |
| **Authors result/conclusion** | From discussion: At pre-intervention, the rates of misconceptions in this student sample (97% had at least one misconception) were strikingly similar to previous studies. At pre-intervention, the average number of misconceptions held by students was 4.0 (out of a maximum of 6.0); at post-intervention this had halved (to an average of 2.0 misconceptions per student) and remained reduced at follow-up (an average of 2.7 misconceptions per student). Both interventions were remarkably effective in reducing the misconceptions of NHST p values and were similarly effective. |
| **Methodological comments**  **Other comments** | Relevant population. Good face validity for outcome questions as they repeat and reproduce results from previous studies. |

| **Author**  **Year**  **Country** | Kühberger et al. 2015  Austria |
| --- | --- |
| **Study design**  **Purpose**  **Procedure/response rate**  **Population** | Cross sectional questionnaire study.  Investigating the intuitive understanding of the notion of significance in their relation to effect size and sample size.  214 students of psychology enrolled in a statistics course at University of Salzburg were sampled. Participants were faced one of two case examples taken from real published articles where one was presented as statistically significant and the other as non-significant. Sequence of cases were altered between groups. Participants were then asked to estimate statistical outcome measures (i.e., sample sizes, means and standard deviations of both groups, Cohen’s d) as they would expect them to be reported in the results section of the paper. After excluding participants with impossible or inconsistent answers and participants with key missing values there were 127 and 133 participants, respectively, included in the analysis for each case, resulting in an analyzed response rate of about 59-62 %.  214 students; 156 females, mean age 23.5 (SD 6.8). |
| **Type of questionnaire**  **Questions about inference**  **Type of inference investigated** | Written questionnaire.  The vignette is available as supplement of the articles. There is no clear description of the outcome question but according to results participants were asked to assess n, Mgroup1, Mgroup2, Mdiff, SDgroup1, SDgroup2 1 and Cohen’s d. Effect sizes were assessed as being “very small (0.00 < d < 0.30)”, “small (0.30 < d < 0.50)”, “medium (0.50 < d < 0.80)” or “large d>0.80)  According to the authors: “the magnitude fallacy” and “the nullification fallacy” |
| **Results** | Results show that participants rated sample sizes in the significant condition and not significant condition only slightly larger, and the effect sizes were rated higher in the significant cases than the non-significant ones.  A minority specified a null effect of a non-statistical finding, but a large proportion assessed the effect to be very small. |
| **Authors result/conclusion** | Conclusions from abstract:  After considerable training in statistics, students largely equate statistical significance with medium to large effect sizes, rather than with large sample sizes. The data show that students assume that statistical significance is due to real effects, rather than to ‘statistical tricks’ (e.g., increasing sample size). |
| **Methodological comments**  **Other comments** | Mere information about whether results are significant or non-significant obscures information about effect size and sample size why it seems unfair to ask anyone to disentangle it. |

| **Author**  **Year**  **Country** | Lecoutre et al.  2003  France |
| --- | --- |
| **Study design**  **Purpose**  **Procedure/response rate**  **Population** | Cross sectional questionnaire study.  To investigate the way experienced users interpret Null Hypothesis Significance Testing (NHST) outcomes indifferent situations.  The responses were gathered individually and were completed by semi directive interviews aiming to compile further comments and justifications. The duration ranged in length from 15 to 25 minutes.  25 professional statisticians from pharmaceutical companies and 20 psychological researchers from various laboratories, all from France. |
| **Type of questionnaire**  **Questions about inference**  **Type of inference investigated** | Written information, verbal responses.  The subjects were presented with the results of a study designed to test the efficacy of a drug by comparing two groups (treatment vs. placebo) of 15 patients each. The following evaluation criterion for the efficiency of the drug was given to the subjects: the drug was to be considered clinically interesting by experts in the field, if the unstandardized difference between the treatment mean and the placebo mean was more than +3.  Four situations were constructed by crossing the outcome of the t test (significant vs. nonsignificant) and the unstandardized observed mean difference D (large vs. small). Two of these situations appeared as conflicting.  1) Nonconflicting t=3.674 p=.001 D=6.07 (large)  2) Nonconflicting t=0.683 p=.50 D=1.52 (small)  3) Conflicting t=3.674 p=.001 D=1.52 (small)  4) Conflicting t=0.683 p=.50 D=6.07 (large)  Questions:  (1) For each of the four situations, what conclusion would you draw for the efficacy of the drug?  (2) Initially, the experiment was planned with 30 subjects in each group and the results presented here are in fact intermediate results. What would be your prediction of the final results for D then t, then for the conclusion about the efficacy of the drug?  (3) From an economical viewpoint, it would of course be interesting to stop the experiment with only the first 15 subjects in each group. For which of the four situations would you make the decision to stop the experiment, and conclude?  Respondents were asked to justify their answers. |
| **Results/Outcomes** | Please see table 3 in the article. In summary:  *Results situation 1 (significant test, large D, nonconflicting situation)*  All subjects but one concluded that the drug was efficacious.  *Results situation 2 (nonsignificant test, small D, nonconflicting situation)*  This situation also gave rise to a considerable consensus for the conclusion: 84% of the statisticians and 85% of the psychologists concluded inefficacy.  *Results situation 3 (significant test, small D, conflicting situation)*  This situation revealed differences between the two groups. The statisticians were relatively homogeneous with80% of them concluding inefficacy, correctly taking into account the smallness of D. On the other hand, the psychologists were divided. Almost half of  them (45%) concluded efficacy of the drug, relying exclusively on the significant test and confusing “statistical significance” with “substantive significance.”  *Results situation 4 (nonsignificant test, large D, conflicting situation)*  This situation was considered as conflicting by a majority (65% of the psychologists and 52% of the statisticians), who did not give a conclusion.  For results about predictions about D, t and efficacy, please see article. |
| **Authors result/conclusion** | From abstract:  On the whole, statisticians and psychologists reacted in a similar way and were very impressed by significant results. It must be outlined that professional applied statisticians were not immune to misinterpretations, especially in the case of nonsignificance. |
| **Methodological comments**  **Other comments** | Small study populations. |

| **Author**  **Year**  **Country** | Lytsy et al.  2022  Sweden |
| --- | --- |
| **Study design**  **Purpose**  **Procedure/response rate**  **Population** | Online survey to the members of the Swedish Society for Medical Statistics and the Swedish Society for Epidemiology and PhD students at the medical and pharmaceutical faculty at Uppsala University. The survey was conducted using a web questionnaire and consisted of 14 questions about inferences of statistically significant findings.  To investigate inferences of statistically significant test results among persons with more or less statistical education and research experience.  All members of the Swedish Society for Medical Statistics and the Swedish Society for Epidemiology (around 300 members in total) and the PhD students (approximately 600) where invited by email to participate.  75 statisticians/epidemiologist and 94 doctoral students agreed to participate.  Two study populations of “statisticians/epidemiologists” (n=75) and “doctoral students” (n=94) were formed by those completing the questionnaire. All but one reported having university degree. The group of statisticians/epidemiologists had a mean age of 48.5 (SD 12.4) years, 62.5% were men, 78.1% had university degree in statistics and 26.6% had a PhD in statistics; 78.1 % of them had authored or co-authored more than 5 scientific publications and 90.6% reported working with statistics professionally. The doctoral students had a mean age of 35.1 (SD 9.0) years, 33.3% were men, no one had a PhD in statistics, 17.3% reported having authored or co-authored more than 5 scientific publications |
| **Type of questionnaire**  **Questions about inference**  **Type of inference investigated** | Web questionnaire.  A researcher wanted to test a hypothesis (H1) and set up a statistical test with a null hypothesis (HO). The significance test resulted in a p-value of 0.024 which was lower than the present level of significance (p<0.05), thus, the result was statistically significant.  1.  What does the test say about HO?  a. It proves that HO is not true  b. It shows that HO is improbable  c. None of the answers above are correct  2. What does the test say about H1?  a. It proves that H1 is true  b. It shows that H1 is probable  c. None of the answers above are correct |
| **Results** | Proportion of responses.  **What does the test say about H0?**  Statisticians/epidemiologists Doctoral students  It proves that H0 is not true 9.4% 24.0%  It shows that H0 is improbable 73.4% 53.3%  None of answers correct 17.2% 22.7%  **What does the test say about H1?**  Statisticians/epidemiologists Doctoral students  It proves that H1 is true 6.2% 12.0%  It shows that H1 is probable 62.5% 62.7%  None of answers correct 31.2 % 25.3%  Correct answer to both questions 12.5% 10.7% |
| **Authors result/conclusion** | This study shows that inferential mistakes of p-values and statistically significant test results persist, also among persons who have substantial statistical education and who work professionally with statistics. The results further provide preliminary evidence that statistical inferences can be biased based on the context. These findings need to be confirmed in future studies. |
| **Methodological comments**  **Other comments** |  |

| **Author**  **Year**  **Country** | McShane et al  2016  United States |
| --- | --- |
| **Study design**  **Purpose**  **Procedure/response rate**  **Population** | Two online surveys.  Study 1: The goal of study 1 was to examine the hypothesis that a focus on statistical significance leads researchers to misinterpret data.  Study 2: The goal of study 2, was to examine whether the observed pattern of results extends from descriptive statements to the evaluation of evidence via likelihood judgments.  Study 1: A link to the survey was sent out to 322 authors in the 2013 volume of the New England Journal of Medicine; about 20 email addresses were incorrect. Seventy-five authors completed the survey, yielding a completion rate of 25%.  Study 2: A link to the survey was sent out to 1111 authors in the 2013 volume of the American Journal of Epidemiology; about 110 email addresses were incorrect. Two hundred ninety-nine authors completed a survey, yielding a completion rate of 30%, 261 completed the survey correctly and were included in analyses.  Authors av articles in 2013 volume of New England Journal of Medicine and American Journal of Epidemiology, respectively. |
| **Type of questionnaire**  **Questions about inference**  **Type of inference investigated** | Web questionnaire,  Study 1  The principal question asked subjects to choose the most accurate description of the results from a study summary that showed a difference in an outcome variable associated with an intervention. Whether this difference attained (p = 0.01) or failed to attain (p = 0.27) statistical significance was manipulated within subjects.  Participants were asked whether a difference between two groups was greater, less, no different or cannot be determined when speaking only about the subjects in the particular study.  Study 2  Subjects completed a likelihood judgment question followed by a choice question. Subjects were randomly assigned to one of four conditions that varied whether the p-value was set to 0.025, 0.075, 0.125, or 0.175. Subjects saw the same p-value in the choice question as they saw in the preceding likelihood judgment question.  Participants were presented with test results where 52% of those who took drug A recovered from the disease and 44% of those who took drug B. There was no difference in terms of probability of recovery between A and B given a significance test resulting in a p-value of 0.025. Participants were then asked whether a randomly drawn person from the same population would be more likely, less likely, equally likely or it cannot be determined, to recover from the disease if given drug A or given drug B. Participants were also asked to a give their own preferred choice, if they were a patient in the population: would they prefer drug A, drug B or being indifferent between drug A and drug B. |
| **Results** | Study 1  For the principal question shown above, the correct answer is option A (i.e greater) regardless of the p-value and the response wording: all four response options are descriptive statements and indeed the average number of post-diagnosis months lived by the subjects who were in Group A was greater than that lived by the subjects who were in Group B (i.e., 8.2 > 7.5). For instance, among participants who saw the response wording above, 95% correctly answered when the p-value was set to 0.01; on the other hand, only 10% correctly answered when the p-value was set to 0.27, with 55% choosing option C and 35% choosing option D.  Study 2  The correct answer is option A because Drug A is more likely to have higher efficacy than Drug B regardless of the p-value. The share of participants who correctly answered the judgment question when the p-value was set to 0.025 was 70% and 81% in the small and large treatment difference conditions, respectively. However, the share of participants who correctly answered the judgment question was substantially lower when the p-value was set to 0.075, 0.125, and 0.175, with no substantial variation in the share answering correctly across these three conditions (16%, 25%, and 16%, respectively, when the treatment difference was small and 21%, 24%, and 22%, respectively, when the treatment difference was large). |
| **Authors result/conclusion** | From discussion:  We have shown that researchers across a variety of fields are likely to make erroneous statements and judgments when presented with evidence that fails to attain statistical significance. These errors pertain to descriptions of data, evaluations of evidence via likelihood judgments, and choices; they do not appear to be moderated by either the magnitude of the treatment difference or the precise size of the p-value associated with the effect. |
| **Methodological comments**  **Other comments** | Relevant population. Poor response rate. Good face validity for outcome questions.  Question 1 in study one is a trick question as the p-value is irrelevant. This, however, was the authors’ point, that inference of evidence tends to be dichotomized. |

| **Author**  **Year**  **Country** | McShane et al  2017  United States |
| --- | --- |
| **Study design**  **Purpose**  **Procedure/response rate**  **Population** | Two online surveys.  Study 1: The purpose was to examine whether the various forms of dichotomization intrinsic to the NHST paradigm would lead even expert statisticians to engage in dichotomous thinking and thus misinterpret data.  Study 2: The purpose was to examine whether the pattern of results observed in Study 1 extends from the interpretation of data to likelihood judgments (i.e., predictions) and decisions (i.e., choices) made based on data.  Study 1: A link to our survey was sent via email to authors publishing Journal of the American Statistical Association in 2010–2011. Of 531 emailed invitations 117 authors responded to the survey, yielding a response rate of 24%.  Study 2: A link to our survey was sent via email to authors publishing Journal of the American Statistical Association in 2012-2013. Of 565 mailed invitations 140 responded yielding a response rate of 27%.  Subjects in study 1 and 2 were authors of articles, described above. |
| **Type of questionnaire**  **Questions about inference**  **Type of inference investigated** | Web questionnaire  Study 1  The principal question asked subjects to choose the most accurate description of the results from a study summary that showed a difference in an outcome variable associated with an intervention. Whether this difference attained (p = 0.01) or failed to attain (p = 0.27) statistical significance was manipulated within subjects.  Participants were asked whether a difference between two groups was greater, less, no different or cannot be determined when speaking only about the subjects in the particular study. Respondents were randomly allocated to have a (irrelevant) p-value of 0.01 or 0.27 included in the information. After answering, the participants were asked the same answer again, with a switched p-value. Participants were further asked about their model assumptions when answering the previous questions.  Study 2  Subjects completed a likelihood judgment question followed by a choice question. Subjects were randomly assigned to one of four conditions that varied whether the p-value was set to 0.025, 0.075, 0.125, or 0.175. Subjects saw the same p-value in the choice question as they saw in the preceding likelihood judgment question.  Participants were presented with test results where 52% of those who took drug A recovered from the disease and 44% of those who took drug B. There was no difference in terms of probability of recovery between A and B given a significance test resulting in a p-value of 0.025. Participants were then asked whether a randomly drawn person from the same population would be more likely, less likely, equally likely or it cannot be determined, to recover from the disease if given drug A or given drug B. Participants were also asked to a give their own preferred choice, if they were a patient in the population: would they prefer drug A, drug B or be indifferent between drug A and drug B. |
| **Results** | Study 1  For the principal question shown above, the correct answer is option A (i.e greater) regardless of the p-value and the response wording: all four response options are descriptive statements and indeed the average number of post-diagnosis months lived by the subjects who were in Group A was greater than that lived by the subjects who were in Group B (i.e., 8.2 > 7.5). However, subjects were much more likely to answer the question correctly when the p-value in the question was set to 0.01 than to 0.27 (84% versus 49%).  Study 2  The issue at variance in both the likelihood judgment question and choice question is fundamentally a predictive one.  The p-value is one measure of the strength of the evidence regarding the likelihood that it is. However, the level of the p-value does not alter the correct response option for either question: the correct answer is option A as Drug A is more likely to be more effective than Drug B in each of the four respective p-value settings.  The proportion of subjects who chose option A for the judgment question dropped sharply once the p-value rose above 0.05 but it was relatively stable thereafter (63% versus 22%, 21%, and 6%, respectively). This provides further evidence that the dichotomization of evidence intrinsic to the NHST paradigm leads even expert statisticians to think dichotomously. |
| **Authors result/conclusion** | From abstract:  Researchers who are primarily statisticians are also prone to misuse and misinterpret p-values thus resulting in similar errors. In particular, we show that statisticians tend to interpret evidence dichotomously based on whether or not a p-value crosses the conventional 0.05 threshold for statistical significance. |
| **Methodological comments**  **Other comments** | Relevant population. Poor response rate. Good face validity for outcome questions.  Question 1 in study one is a trick question as the p-value is irrelevant. This, however, was the authors’ point, that inference of evidence tends to be dichotomized. |

| **Author**  **Year**  **Country** | Mittag et al.  2000  US |
| --- | --- |
| **Study design**  **Purpose**  **Procedure/response rate**  **Population** | Cross sectional questionnaire  To explore current perceptions of AERA members regarding statistical significance tests and perceptions regarding other statistical issues, such as score reliability.  A two-sided one-sheet survey was distributed to a stratified random sample of 1127 (about 4%) AERA members (American Educational Research Association). Of 246 returned surveys 225 (21.7%) were used in analyses.  AERA members across 50 states in the US, about 49.3% male and 83.6% with a doctoral degree. |
| **Type of questionnaire**  **Questions about inference**  **Type of inference investigated** | Written two-page questionnaire.  Twenty-nine items evaluated nine clusters of perception. Several questions regarded of statistical practices and views on matter such as power, replication, regression as well as views on controversies.  Several of the reported questions were considered to be about inference of statistically significant results. Responses to questions were given in a 1-5 Likert scale were (1= disagree and 5=agree)   1) All that significance means is that the researcher rejected the null hypothesis. 2) A Type II error is impossible if the results are statistically significant. 3) Significance tests are partly a test of whether the researcher had a large sample. 4) Every null hypothesis will eventually be rejected at some sample size. 5) Statistically significant results are more noteworthy when sample sizes are small. 6) If a dozen different researchers investigated the same phenomenon using the same null hypothesis, and none of the studies yielded statistically significant results, this means that the effects being investigated were not noteworthy or important. 7) Smaller p values provide direct evidence that study effects were larger. 8) The p-values reported in different studies cannot be readily compared, because these values are confounded with the different samples sizes across studies.   Perceptions about statistically significant findings. |
| **Results/Outcomes** | Results to questions were presented as 95% CI:s (of the 1-5 scale) in figures. Intervals below are approximate, please see figures in original publication.   1) All that significance means is that the researcher rejected the null hypothesis. (2.7-3.4) 2) A Type II error is impossible if the results are statistically significant. (1.9 – 2.5) 3) Significance tests are partly a test of whether the researcher had a large sample. (2.9-3.5) 4) Every null hypothesis will eventually be rejected at some sample size. (2.6-3.3) 5) Statistically significant results are more noteworthy when sample sizes are small. (2.8-3.4) 6) If a dozen different researchers investigated the same phenomenon using the same null hypothesis, and none of the studies yielded statistically significant results, this means that the effects being investigated were not noteworthy or important. (3.7-4.3) 7) Smaller p values provide direct evidence that study effects were larger. (3.5-4.1) 8) The p-values reported in different studies cannot be readily compared, because these values are confounded with the different samples sizes across studies. (2.9-3.5) |
| **Authors result/conclusion** | From Conclusions:  The present results provide one snapshot of the contemporary thinking of members of one national research association. |
| **Methodological comments**  **Other comments** | Questions seem to have poor face validity.  Low response rate. |

| **Author**  **Year**  **Country** | Monterde-i-Bort et al.  2010  Spain |
| --- | --- |
| **Study design**  **Purpose**  **Procedure/response rate**  **Population** | Written questionnaire sent by post.  Estimate the prevalence of false beliefs and inappropriate interpretations about statistical methodology.  Sample of 120 research professors at psychology schools from Spanish universities with research and teaching experience. The questionnaire was sent and returned by ordinary post; valid questionnaires represented a response ratio of about 30%.  Likely about 36 participants (about 30% of the sample of 120), being research professors in methodology (40%) or other areas (55%), and 5% who did not provide this information. |
| **Type of questionnaire**  **Questions about inference**  **Type of inference investigated** | Written questionnaire.  The authors used a “psychometrics group instrument” used in previous studies, see Mittag et al. It was developed to determine the participants’ perceptions about the statistical significance tests and other statistical questions. It consists of 29 items, which are responded to on a five-point Likert type scale (1=“disagree”, 2=“disagree somewhat”, 3=“neutral”, 4=“agree somewhat”, and 5=“agree”).  Results presented as means with 95% confidence intervals.  Most questions were not relevant in terms of assessments of statistical inferences, the ones that were are listed below.  Perceptions of statistical inference. |
| **Results/Outcomes** | Statements with mean agreement on 5-point Likert scale (95% CI):   - All that significance means is that the researcher rejected the null hypothesis 3.71 (3.43 to 3.99) - Finding that p<.05 is one indication that the results are important 3.89 (3.67 to 4.11) - Studies with non-significant results can still be very important. 1.99 (1.81 to 2.17) - Smaller and smaller values for the calculated p indicate that the results are more likely to be replicated in future research. 3.53 (3.28 to 3.78) - Significance tests evaluate the probability that the results for the sample are the same in the population. (2.65 2.38 to 2.92)   Statistically significant results are more noteworthy when sample sizes are small. 2.90 (2.63 to 3.17)  Smaller ‘p’ values provide direct evidence that study effects were larger. 3.54 (3.29 to 3.79)  The ‘p’ values reported in different studies cannot be readily compared, because these values are confounded with the different sample sizes across studies. 3.35 (3.09 to 3.61)  Smaller and smaller values for the calculated ‘p’ indicate that the results are more and more likely to be replicated in future research. 3.53 (3.28 to 3.78)  The ‘p’ values that are calculated in a given study test the probability of the results occurring in the sample, and not the probability of results occurring in the population. 2.34 (2.09 to 2.59)  Significance tests evaluate the probability that the results for the sample are the same in the population. 2.65 (2.38 to 2.92) |
| **Authors result/conclusion** | From authors’ conclusions: The characteristics of the sample and the scope of the results are described, and a critical reflection is carried out on typical misunderstandings in interpretation that show a worrisome lack of understanding of some basic methodological and statistical concepts. |
| **Methodological comments**  **Other comments** | Relevant population. Use of set of inference questions that has been used previously. Yet, ratings of perceptions of statements seem difficult to comprehend.    Overall results broadly consistent with previous studies using same set of outcome questions. |

| **Author**  **Year**  **Country** | Msaouel et al  2014  Greece |
| --- | --- |
| **Study design**  **Purpose**  **Procedure/response rate**  **Population**  **Inclusion criteria** | Multi-institutional cross-sectional paper and pen survey.  To evaluate the biostatistical competency and cognitive biases of medical residents.  The survey was conducted from January to March 2010, and the participants were chosen from eight major Greek hospitals in the city of Athens. Out of 1,272 eligible residents in all hospitals, 200 were randomly selected by a computerized method to participate in the sample. The residents were asked to return the completed questionnaires to a sealed box provided in each hospital. The survey response rate was 76.5% (153/200)  Study sample (n153) of Greek medical residents working in medical specialist (46.4%), surgical specialty (43.8%) or diagnostic and laboratory specialist (9.8%). Median age was 32 (range 24-44) and 26.8% were female. The majority of residents (81.7%) reported prior attendance of biostatistics courses, which mainly occurred during medical school. |
| **Type of questionnaire**  **Questions about inference**  **Type of inference investigated** | Paper and pen survey at residents’ hospital.  There were seven questions about biostatistics knowledge. One of these were about p-value and another about CI. Each vignette was followed by four multiple choice statements and respondents were asked to select one, the most correct.  The p-value vignette was:  “You read in a clinical trial report that the treatment under investigation showed statistically significant increase in overall survival compared to the placebo control group (pB0.05). Which of the following statements is most correct?”  The CI vignette was:  “You read in a medical paper that the RR for a disease in individuals who are exposed to a risk factor is 1.64 (95% CI1.102.45) compared to otherwise similar individuals who are not exposed to this risk factor. Which of the following statements is most correct?” |
| **Results** | P-value question, statements and proportions of responders to each statement.  bold type indicates correct statement according to authors.   - The treatment is definitely better compared to placebo (37.9%) - The observed increase in overall survival is so large that there is less than a 5% chance that placebo is equal to the treatment (19.6%) - **If the treatment does not actually increase survival, then the chance of obtaining the observed (or even greater) increase in survival is less than 5%. (26.8%)** - I do not know and do not wish to guess, (15.7%).   CI question statements and proportions of responders to each statement.  bold type indicates correct statement according to authors.   - 95% of those exposed to the risk factor had RR between 1.10 and 2.45, (28.8%) - **There is a 95% likelihood that the true RR falls between 1.10 and 2.45, (33.3%)** - Approximately 50% of individuals exposed to the risk factor had a RR of 1.64, (5.9%) - I do not know and do not wish to guess. (32%) |
| **Authors result/conclusion** | Authors conclusion based on results from seven questions about biostatistics knowledge questions and two questions about cognitive biases:  “The measured biostatistics performance in our survey indicates that an alarmingly large number of medical residents are unable to correctly interpret crucial statistical concepts that are commonly found in the medical literature.” |
| **Methodological comments**  **Other comments** | Relevant population of clinicians. Fairly good response rate.  Arguably authors statement about inferences of CI:s: “There is a 95% likelihood that the true RR falls between 1.10 and 2.45” is not correct. Whether the true RR is included in the presented interval is either true or false and admits no probability or likelihood statement. |

| **Author**  **Year**  **Country** | Nelson et al  1986  US |
| --- | --- |
| **Study design**  **Purpose**  **Procedure/response rate**  **Population** | Cross sectional mailed questionnaire.  Replicate and expand the work of Rosenthal & Gatio (1963) and Beauchamp and May (1964).  Names from authors, editors and APA-members were gathered and names were randomly chosen resulting in a sample of 294 persons who were mailed a questionnaire. Half of them were randomly assigned to a high importance condition and half to an average important condition. Data collection was performed 1984. 85 responded, response rate 29%.  Authors, reviewers and APA-members. |

| **Type of questionnaire**  **Questions about inference**  **Type of inference investigated** | Written mailed.  In both treatment conditions the psychologists were instructed to rate the degree of confidence or belief they were likely to have in the working hypothesis within the context of each of the following p levels: .001, .005, .01, .02, .03, .04, .05, .06, .07, .08, .09, .10, .15, .20, .25, .30, .40, .50, .70, and .90.  The possible ratings were extreme confidence or belief (5), great confidence or belief (4), moderate confidence or belief (3), mild confidence or belief (2), minimal confidence or belief (1), and complete absence of confidence or belief (0). |
| --- | --- |
| **Results/Outcomes** | The largest effect was due to significance level. Of course, psychologists expressed greater confidence in lower levels of significance, but that finding was expected.  There were also results of a cliff-hanger effect for p<0.05 and p<0.10. |
| **Authors result/conclusion** | Consistent with earlier findings noted previously, we found that psychologists' degree of confidence in research results dropped off most abruptly when the level of significance was just above .05, and a secondary "cliff effect" occurred at the 0.10 level. |
| **Methodological comments**  **Other comments** |  |

| **Author**  **Year**  **Country** | Oakes M  1986  Great Britain |
| --- | --- |
| **Study design**  **Purpose**  **Procedure/response rate**  **Population** | Likely a written questionnaire.  To test inferences of null hypothesis significance testing.  Participants were represented with a research result and were asked to mark each of six following statements as true or false. The subjects were informed to not anticipate any particular true false- ratio of the statements and that if any statement did not follow rigorously from the premises in the first paragraph, then that statement was false.  70 academic psychologists, who all university lecturers, research fellows or post doctorate studies with at least two years´ research experience. |
| **Type of questionnaire**  **Questions about inference**  **Type of inference investigated** | Written  Suppose you have a treatment which you suspect may alter performance on a certain task. You compare the means of your control and experimental groups (say 20 subjects in each sample). Further, suppose you use a simple independent means t test and your result is (t=2.7, d.f. 18, p = 0.01). Please mark each of the statements below as ‘true’ or ‘false’.   a) You have absolutely disproved the null hypothesis (that there is no difference between the population means). b) You have found the probability of the null hypothesis being true. c) You have absolutely proved your experimental hypothesis (that there is a difference between the population means). d) You can deduce the probability of the experimental hypothesis being true. e) You know, if you decided to reject the null hypothesis, the probability that you are making the wrong decision. f) You have a reliable experimental finding in the sense that if, hypothetically, the experiment were repeated a great number of times, you would obtain a significant result on 99% of occasions   Test of logic of NHST |
| **Results/Outcomes** | All statements a-f are false. The frequency of ‘true’ responses (i.e frequency of misunderstandings).  Number (f) percent  a) The null hypothesis is absolutely disproved 1 1.4  b) The probability of the null hypothesis has been found 25 35.7  c) The experimental hypothesis is absolutely proved 4 5.7  d) The probability of the experimental hypothesis can be deduced 46 65.7  e) The probability that the decision taken is wrong is known 60 85.7  f) Replications have a 0.99 probability of being significant 42 60.0 |
| **Authors result/conclusion** | From text:  “…the sheer frequency with which false or meaningless statements are, on sober reflection described as true is remarkable. Only three subjects, 4%, correctly described all six statements as false. Second, the notion that associated probability is a direct index of experimental replicability is surprisingly widespread. Sixty per cent of subject endorsed this statement (f). Third, the responses to statements (b, d, e) reveal fundamental inconsistences win psychologists’ apprehensions of significance testing. |
| **Methodological comments**  **Other comments** |  |

| **Author**  **Year**  **Country** | Poitevineau  2001  France |
| --- | --- |
| **Study design**  **Purpose**  **Procedure/response rate**  **Population** | Written survey.  Our aim was to identify distinct categories of subjects, possibly corresponding to different conceptions of statistical inference, referring in particular to Neyman–Pearson, Fisher, and Bayes. With this purpose in mind, the instructions were made more explicit than those in the original experiment. It was specified that confidence was related to the effect of the experimental treatment, and the subjects were asked to state their degree of confidence in the hypothesis that the “experimental treatment really has an effect.”  The subjects carried out the task individually. In order to clarify the meaning of the sample size, it was specified that the test was Student’s t test for paired groups. Twelve p values (.001, .01, .03,.05, .07, .10, .15, .20, .30, .50, .70, .90) crossed with two sample sizes (n = 10 and n = 100, as in the original experiment) were presented at random on separate pages of a notebook.  18 psychological post graduate researchers from various universities in France. |
| **Type of questionnaire**  **Questions about inference**  **Type of inference investigated** | Written questionnaire.  The subjects were asked to mark a point on a nongraduated 1-dm (i.e., 10-cm) line corresponding to their degree of confidence, from zero confidence (0, left extremity of the line) to complete confidence (1, right extremity). |
| **Results/Outcomes** | The average curves, plotted in Figure 1A, were rather similar to those in Rosenthal and Gaito’s (1963) article, with a degree of confidence that was always greater for n = 100 than for n = 10. A .05 cliff effect was apparent for the two sample sizes. Nevertheless, these average curves were fairly well fitted by an exponential function |
| **Authors result/conclusion** | From abstract:  Detailed analysis of individual curves reveals that the attitude of researchers.  toward p values is far from being as homogeneous as might be expected. However, most  psychological researchers in our study rated graduated confidence judgments, as either exponential or linear. Only a minority of all-or-none respondents exhibited an abrupt drop in confidence |
| **Methodological comments**  **Other comments** | Very small sample. Questionable validity. The study is a replication of Robert Rosenthal & John Gaito (1963) The Interpretation of Levels of Significance by Psychological Researchers, The Journal of Psychology, 55:1, 33-38,  Arguably, p-values are not valid measures or indices of once confidence in the posterior probability of the alternative hypothesis. |

| **Author**  **Year**  **Country** | Polychronopoulou et al.  2011  Greece |
| --- | --- |
| **Study design**  **Purpose**  **Procedure/response rate**  **Population** | Written questionnaire  To explore the level of postgraduate students’ knowledge on biostatistics.  The questionnaire and a covering letter with directions on the correct set and mode of responding, was given in person to all orthodontic programme directors on a meeting in Geneva, Switzerland, in 2008. The intention was that these persons would distribute the questionnaire to the students in their orthodontic postgraduate programmes. After completion questionnaires were returned by the programme directors. Completed questionnaires were returned from 127 participants from 21 postgraduate programmes in 10 European countries. Response rate is unknown.  127 responders from 10 European countries. 21% of respondents had doctoral degree, 28.3% had master’s degree. 34.7% reported having previous training in epidemiology/biostatistics. |
| **Type of questionnaire**  **Questions about inference**  **Type of inference investigated** | Written questionnaire.  The questionnaire had 20 questions of which 13 were knowledge based. One question was about interpreting a non-statistically significant finding. The question was phrased:  16. In a randomized clinical trial of the use of aspirin and placebo to prevent mild post banding pain, 47% of the patient’s receiving aspirin and 48% of those receiving placebo reported pain. In reporting this overall finding the authors stated that p>.05. This means:  a. The chance is 95% that the study is correct  b. The chances are greater than 1 in 20 that a difference would be found again if the study were repeated  c. The probability is less that 1 in 20 that a difference this large could occur by chance alone  d. The probability is greater than 1 in 20 that a difference large could occur by chance alone  NHST |
| **Results/Outcomes** | Correct interpretation of the question about a statistically non- significant finding 33.0% (95% CI: 24.7 to 41.3 |
| **Authors result/conclusion** | From results:  Of the 106 participants who felt confident about P-value interpretation, only 37.7 per cent gave the correct answer to the corresponding question listed in the knowledge section.  From discussion  The results of this survey showed that orthodontic postgraduate students’ knowledge on biostatistics in Europe is only influenced by previous relevant education. |
| **Methodological comments**  **Other comments** | Authors did not report information on which response alternatives that are correct. |

| **Author**  **Year**  **Country** | Rajcani 2023 et al.  2023  Slovakia |
| --- | --- |
| **Study design**  **Purpose**  **Procedure/response rate**  **Population** | Survey  To identify the most common errors in understanding and interpretation of statistical concepts and es­timate their prevalence among Slovak psy­chology researchers a decade into the repro­ducibility crisis  In the present survey, we contacted psychology researchers, lecturers, and doctoral students from all universities in Slovakia and the Slovak Academy of Sciences via email. Together we re­ceived answers from 65 participants. Responses of 40 (61.5%) were used in analyses of statistical inference. Some of the questions were based on previous surveys (Haller & Kraus, 2002), on misconceptions discussed in the published literature (e.g., Gelman & Stern, 2006; Greenland et al., 2016), and on the materials from the course “Improving your statistical inferences” by Lakens (2016).  65 researchers, 63.5% women. Average 8.6 years in academia and 43.4 % teaching methodology or statistics courses. |
| **Type of questionnaire**  **Questions about inference**  **Type of inference investigated** | Online survey.  There were 14 questions in the survey, 6 about NHST and 3 about confidence intervals.  Questions about NHST:   1. P-value represents the probability of the null hypothesis being true (Yes/No/NA). 2. If p = 0.01 in one study and p = 0.09 in a second study, does it mean that a larger effect was observed in the first study compared to the second study? Yes/No/NA 3. P-value represents the probability that our result is the result of chance (Yes/No/NA). 4. If we obtain a statistically non-significant result (p > 0.05), we accept the null hypothesis (i.e., we conclude that the examined effect does not exist) Yes/No/NA. 5. If you test the same hypothesis in two populations (e.g., male and female), with p < 0.05 in one sample and p > 0.05 in the other sample, does it mean that there is a significant difference between the groups? (Yes/No/NA) 6. If there is no examined effect in the population (i.e. the null hypothesis is true):   there is a greater chance that we will get a high p-value than a low one there is a greater chance that we will get a low p-value than a high one /all p-values are equally likely/I don’t know/I don’t want to answer. |
| **Results/Outcomes** | *Prevalence of misconceptions about p-values (N = 47).*  Correct answers in **bold type face.**  1. P-value represents the probability of the null hypothesis being true.  Yes/agree 35%, **No/disagree 52%** Does not know/does not want to answer 13%  2. If p = 0.01 in one study and p = 0.09 in a second study, does it mean that a larger effect was observed in the first study compared to the second study?  Yes/agree 22%, **No/disagree 70%** Does not know/does not want to answer 8%  3. P-value represents the probability that our result is the result of chance.  Yes/agree 41%, **No/disagree 43%** Does not know/does not want to answer 15%  4. If we obtain a statistically non-significant result (p > 0.05), we accept the null hypothesis (i.e., we conclude that the examined effect does not exist).  Yes/agree 53%, **No/disagree 33%** Does not know/does not want to answer 13%   5. If you test the same hypothesis in two populations (e.g., male and female), with p < 0.05 in one sample and p > 0.05 in the other sample, does it mean that there is a significant difference between the groups?   Yes/agree 40%, **No/disagree 44%** Does not know/does not want to answer 16%   6. If there is no examined effect in the population (i.e. the null hypothesis is true):  - there is a greater chance that we will get a high p-value than a low one 49% - there is a greater chance that we will get a low p-value than a high one 9% - **all p-values are equally likely 20%** - I don’t know/I don’t want to answer 22%   **Confidence intervals.**  Participants were asked 3 questions about confidence intervals:  1) If they compute a 95% confidence interval around the mean, it means that they can be 95% sure that the population average will be in this interval?   - 48%, 95%CI [32%,63%] answered that they agree **(correct according to authors)** - 25%, 95%CI [13%,40%] disagreed - 27%, 95%CI [15%,43%] stated that they don’t know/do not want to answer.  2) Does the size of the confidence interval depend on the sample size?  - 45%, 95%CI [30%,61%] answered yes, with increasing sample size, the interval is narrower **(correct according to authors)** - 9%, 95%CI [3%,22%] of the participants answered yes, with increasing sample size, the interval is wider - 16%, 95%CI [7%,30%] stated no, the range of the interval does not depend on the sample size - 30%, 95%CI [17%,45%] stated that they don’t know/do not want to answer.  3) If you were to replicate the study, is there the 95% probability that your result (e.g., mean) will fall within the 95% confidence interval of the original study?  - 30%, 95%CI [17%,45%] of the participants answered yes - 36%, 95%CI [22%,52%] stated no **(correct according to authors)** - 34%, 95%CI [20%,50%] stated that they don’t know/do not want to answer |
| **Authors result/conclusion** | Misunderstandings about NHST and p-value, which have been mentioned in the literature for years, are still common among Slovak researchers. |
| **Methodological comments**  **Other comments** | The correct answers of the CI questions was controlled by contact with first author |

| **Author**  **Year**  **Country** | Rosenthal et al  1963  US |
| --- | --- |
| **Study design**  **Purpose**  **Procedure/response rate**  **Population** | Cross sectional questionnaire  To investigate how psychological researchers interpret various levels of significance.  Nine faculty members with a PhD and 10 graduate students at a Psychology participated in a written survey.  Se above. |
| **Type of questionnaire**  **Questions about inference**  **Type of inference investigated** | Written questionnaire.  Subjects completed a questionnaire requesting them to rate their degree of belief or confidence in a variety of p levels: once assuming an n of 10, and then assuming an n of 100. The possible ratings they were to assign to each p level were as follows:  5: extreme confidence or belief  4: great confidence or belief  3: moderate confidence or belief  2: mild confidence or belief  1: minimal confidence or belief  0: complete absence of confidence or belief |
| **Results/Outcomes** | From results:  Figure 1 shows clearly not only that greater confidence was placed in lower p levels but also that greater confidence (1/3 of a unit of confidence, on the average) was placed in p levels based on the larger n. In addition, graduate students tended to show greater confidence (1,2 of a unit of confidence, on the average) in any given p level than did faculty members. |
| **Authors result/conclusion** | From discussion:  The relationship between degree of confidence and magnitude of p level appeared to be exponential regardless of sample size assumed and type of subjects employed. Subjects had greater confidence in a given p level when it was associated with a larger sample size suggesting that investigators use the probability of both Type I and Type II errors as criteria of "belief." |
| **Methodological comments**  **Other comments** |  |

| **Author**  **Year**  **Country** | Scheutz et al  1988  Denmark |
| --- | --- |
| **Study design**  **Purpose**  **Procedure/response rate**  **Population** | Cross-sectional multiple-choice questionnaire  To assess the knowledge of elementary statistical expressions in Danish dentists.  250 dentists were randomly selected from a register of a register of all Danish dentists. Answers were given anonymously. Of the dentists, 129 (52%) replied; 125 had completed the test, two had retired and two questionnaires were unsatisfactorily completed.  The test was also given without prior warning to 38 dental students in conjunction with their last lecture in statistics 2 months ahead of their compulsory examination; 27 (71%) wished to participate and completed the multiple-choice test.  The distribution of the 125 dentists: 64% general practitioners, 32% pedodontists, 2% hospital employed, 2% from a dental faculty. |
| **Type of questionnaire**  **Questions about inference**  **Type of inference investigated** | Written questionnaire, according to authors the same that was used in Wulff et al.  There were 11 questions of which 3 were about inferences.   A) A controlled trial of a new treatment led to the conclusion that it is significantly better than placebo: P<0.05. Which of the following statements do you prefer?    a. It has been proved that the treatment is better than placebo.    b. If the treatment is not effective, there is less than a 5% chance of obtaining such results. (correct answer)    c. The observed effect of the treatment is so large that there is less than a 5% chance that the treatment is no better than placebo.    d. do not really know what a P-value is and do not want to guess. B) A research team wishes to examine whether or not the ingestion of licorice decreases the plasma concentration of magnesium. Twenty-three volunteers ingest a considerable amount of licorice and no significant change in the serum magnesium is found (P>0.05). Which of the following statements do you prefer?    a. There is more than a 5% chance of obtaining such results if licorice does not decrease the serum magnesium. (correct answer)    b. There is only a small probability of obtaining such results if licorice does decrease the serum magnesium.    c. The research workers ought to have studied more volunteers, then the difference would have become significant.    d. I do not know what P-values are and do not want to guess. C) A new drug was tested independently in two randomized controlled trials. The trials appeared comparable and comprised the same number of patients. One trial led to the conclusion that the drug was effective (P<0.05). The actual p-values were 0.041 and 0.097. Which of the following interpretations do you prefer?    a. The first trial gave a false-positive result.    b. The second trial gave a false-negative result.    c. Obviously, the trials were not comparable after all.    d. One must not attach too much importance to small differences between p-values. (correct answer)    e. I do not understand the problem and do not want to guess. |
| **Results/Outcomes** | Authors report percentage distribution for dentists and dental students separately (dentists – dental students)  A)   a) It has been proved that the treatment is better than placebo. (18 -4) b) If the treatment is not effective, there is less than a 5% chance of obtaining such results. (correct answer) (8 – 33) c) The observed effect of the treatment is so large that there is less than a 5% chance that the treatment is no better than placebo. (50-56) d) I do not really know what a P-value is and do not want to guess. (25-7)   B)   a) There is more than a 5% chance of obtaining such results if licorice does not decrease the serum magnesium. (correct answer) (32-30) b) There is only a small probability of obtaining such results if licorice does decrease the serum magnesium. (24 – 48) c) The research workers ought to have studied more volunteers, then the difference would have become significant. (16 – 19) d) I do not know what P-values are and do not want to guess. (28 – 4)   C)   a) The first trial gave a false-positive result. (6 – 4) b) The second trial gave a false-negative result. (1 – 4) c) Obviously, the trials were not comparable after all. (34 – 52) d) One must not attach too much importance to small differences between p-values. (correct answer) (26 – 22) e) I do not understand the problem and do not want to guess. (34 – 19) |
| **Authors result/conclusion** | From abstract:  It is concluded that the statistical knowledge of most dentists, and to a lesser degree also dental students, is so limited that they cannot be expected to be critically against or to draw the right conclusions from those statistical analyses with which they are confront. |
| **Methodological comments**  **Other comments** |  |

| **Author**  **Year**  **Country** | Tam et al  2018  Australia |
| --- | --- |
| **Study design**  **Purpose**  **Procedure/response rate**  **Population** | Web-based questionnaire.  The study had three purposes: 1) qualitatively elaborate on the participant’s conceptualisation of ‘P = 0.05’ within a scenario 2) test P value interpretation ability (whether they identified a common erroneous interpretation) with a dichotomous choice question, and 3) measure their confidence in their answer with a Likert scale.  A link to the web questionnaire was posted in Facebook group for” GPs Down Under’ (GPDU). Responses were collected until three consecutive days with no replies. Of 272 participants hat started the questionnaire, 247 respondents completed it (91% completion rate)  Respondents were GP:s mainly from Australia. 33.6 were male. Mean age was 39.6 years (SD 8.9). 82.2 % reported having research experience. |
| **Type of questionnaire**  **Questions about inference**  **Type of inference investigated** | Web questionnaire.  Participants were given this scenario: A study comparing a new antihypertensive to an older agent, with blood pressure as the primary outcome, is published in a medical journal. In the article’s conclusion, the authors claim that, the new drug was superior to the old drug at lowering blood pressure (P = 0.05). First question was qualitative – results not reported here.  Quantitative Question 1:  This means that there is a 5% probability that this result is due to chance alone, or, there is a 95% probability that the conclusion is true. Please select the option that BEST MATCHES your understanding of P values: •  The reader’s interpretation is mostly FALSE [note: correct response]  The reader’s interpretation is mostly TRUE  Quantitative question 2  Question: Please indicate how CONFIDENT you are of your answer: Not at all – Slightly – Somewhat – Very – Entirely [Labelled 5-point Likert scale] |
| **Results/Outcomes** | ‘Mostly FALSE’ was the correct response to the question; ‘this means that there is a 5% probability that this result is due to chance alone, or, there is a 95% probability that the conclusion is true’.2,10 Only 29% (95% confidence interval: 24, 35) or 72/ 247 respondents answered this question of P value interpretation ability correctly.  Notably, there was a confidence–ability gap – self-reported confidence was not associated with better performance in the ‘dichotomous choice’ question (MannWhitney U, P = 0.132). |
| **Authors result/conclusion** | P value misunderstanding is pervasive and might be influenced by a few central misconceptions |
| **Methodological comments**  **Other comments** | The quantitative question offers a statement that includes to interpretations: “This means that there is a 5% probability that this result is due to chance alone, or, there is a 95% probability that the conclusion is true”. |

| **Author**  **Year**  **Country** | Vallecillos  2000  Spain |
| --- | --- |
| **Study design**  **Purpose**  **Procedure/response rate**  **Population** | Cross sectional survey followed by an interview in a small sample.  To test the respondents understanding of the overall logic of the test of hypothesis and the type of validation of the hypothesis that is obtained using this procedure.  An experimental sample was made up by a total of 436 University students who responded to one question about inference and were then asked to support their answer in text.  436 University students studying seven different Degree subjects: Civil Engineering,  Computer Science, Pedagogy, Psychology, Medicine, Mathematic and Business Studies. |
| **Type of questionnaire**  **Questions about inference**  **Type of inference investigated** | Written questionnaire.  One item proposed that:  “A statistical hypotheses test, when properly performed, establishes the truth of one of the two null or alternative hypotheses.”  Respondents were asked true indicate whether this is True / False and asked to support their answer in text. |
| **Results/Outcomes** | Results are given for all specialities separately in the article. The overall result was.  True 186 (42.7%), False 210 (48.2%) Blank 40 (9.2%)  The written arguments were classified by the authors as being correct, partly correct and various mistakes and arguments that are confusing. |

| **Authors result/conclusion** | From authors discussion.  The errors shown on the subject point to a misunderstanding by a noticeable percentage of students, of basic aspects referring to the overall logic of the procedure of the hypotheses test. |
| --- | --- |
| **Methodological comments**  **Other comments** |  |

| **Author**  **Year**  **Country** | van den Akker et al  2023  The Netherlands |
| --- | --- |
| **Study design**  **Purpose**  **Procedure/response rate**  **Population** | Cross sectional survey  To test academics’ statistical intuitions when assessing the results of multiple (four) experiments.  Two studies were carried out, one in 2014 and one in 2022.  Study 1: A total of 2592 valid email addresses were obtained to researchers published in journals about social psychology/experimental psychology or editors/reviewers at such journals. Responses were obtained from 228 (17.6%) authors and 277 (21.4%) reviewers.  The study involved eight different scenarios, each presenting the results of four experiments which were described as “significant” or “non-significant”.  Respondents were then asked to indicate their belief in the theory on the basis of the presented evidence by means of a slider bar, with points going from low probability (0%) to high probability (100%) of the theory being correct.  Study 2:  A total of 19694 valid email addresses were obtained to researchers published in journals about social psychology/experimental psychology or editors/reviewers at such journals. 1334 responded resulting in a response rate of 6.8%.  Respondents were then asked to indicate their belief in the theory on the basis of the presented evidence by means of a slider bar, with points going from low probability (0%) to high probability (100%) of the theory being correct.  Authors and editors/reviewers in journals in social and experimental psychology with a 5-year impact of at least 2 |
| **Type of questionnaire**  **Questions about inference**  **Type of inference investigated** | Online questionnaire  Participants were presented different scenarios of significant or non-significant results of four studies and were then asked to estimate, using a slidebar, “the probability that the theory is correct” (0-100). There were also questions about the strategy of inference. |
| **Results/Outcomes** | Several results displayed. In general, respondents’ belief in the theory (posterior probability) increased with increasing number of cumulative significant results.  Study 1:  Sig. Findings posterior probability (SD)  k = 0 25.33 (16.83)  k = 1 33.04 (17.14)  k = 2 49.34 (15.27)  k = 3 65.77 (16.18)  k = 4 73.02 (17.65)  Study 2:  Sig. Findings posterior probability (SD)  k = 0 23.98 (18.50)  k = 1 33.51 (19.04)  k = 2 46.67 (17.52)  k = 3 61.15 (18.77)  k = 4 71.08 (18.47)  As expected, average belief increased with the number of statistically significant results |
| **Authors result/conclusion** | Across two vignette studies we found that, on average, the number of significant results was positively related to researchers’ belief in the underlying theory. |
| **Methodological comments**  **Other comments** | Poor response rate. |

| **Author**  **Year**  **Country** | Vera-Ponce et al  2022  Peru |
| --- | --- |
| **Study design**  **Purpose**  **Procedure/response rate**  **Population** | Cross sectional survey, online form  To determine the factors associated with knowledge about p-value in a sample medicine students.  Participants were university students who were recruited through social media: Facebook and WhatsApp. A total of 1192 students were surveyed; the response rate is not reported.  Of the responders, 57.6 % were female and 54.9% were between the 10th and 14th semester of their medical studies. Only 28.9% belonged to a public university and 36.0% took an external course in biostatistics, epidemiology, or research. Students younger than 18 years, in semester 1-3 and those not completing the questionnaire were excluded. |
| **Type of questionnaire**  **Questions about inference**  **Type of inference investigated** | Online questionnaire  There were 11 questions about the p-value. The questions are formulated as statements about the p-value and responses were given as True/False/Don't know)  NHST |
| **Results/Outcomes** | Results were presented as the proportion of correct answers (with 95% C). However, it is not stated what the authors consider being the correct answer.  Statement with estimated proportion of being correct (95% CI)  1 The p-value is a probability 72,73 (70,13 - 75,19)  2 A non-significant p-value (p> 0.05) indicates that the null hypothesis is true 61,99 (70,13 - 75,19)  3 A non-significant p-value (p> 0.05) indicates that the treatment effect under analysis is not clinically important. 56,69 (50,85 - 56,51)  4 A non-significant p-value (p> 0.05) indicates that both treatments are similar. 39,43 (36,69 - 42,24)  5 p-value indicates the probability that the null hypothesis is true given the results of our study 64,18 (61,41 - 66,85)  6 A non-significant p-value (p> 0.05) indicates that we should accept the null hypothesis. 54,53 (51,69 - 57,34)  7 If we obtain a significant p-value (p<0.05), we should reject the null hypothesis 48,32 (45,49 - 51,16)  8 The p-value obtained (p = 0.02) indicates the probability of obtaining similar results if the same study is repeated with a similar sample. 21,39 (19,15 - 23,82)  9 A statistically significant result (p <0.05) indicates that the treatment effect under analysis is clinically significant 47,73 (44,91 - 50,58)  10 The p value observed in our study was significant (p = 0.02). This confirms that the treatment effect was greater than that observed in a similar study with a p value = 0.04 13,92 (12,07 - 16,01)  11 Which of the following statements is the definition of a p-value? 55,96 (53,12 - 58,76)  Those having 6 or more correct answers were categorized as having “sufficient knowledge" The authors also performed a multiple regression analysis investigating factors prediction being correct. |
| **Authors result/conclusion** | Authors conclusion: It was evidenced that a little more than half understood the biostatistical results reported in the medical literature |
| **Methodological comments**  **Other comments** | The correct answers to the statements are not given, making it difficult to understand if the authors themselves have correct understanding of which statements are true or false. The corresponded author was contacted, but did not reply.  The survey raises several questions: it is framed as being an about knowledge of the p-value, but several of the questions goes beyond significance testing and asks about inferences of NHST. Furthermore, since the authors do not state which answer they consider correct for each statement, it is not possible for a reader to know whether the results are trustworthy. Arguably, only statement 1 is correct.  We have emailed the corresponding author of the article to clarify, without getting any response. |

| **Author**  **Year**  **Country** | Weiss  1980  USA |
| --- | --- |
| **Study design**  **Purpose**  **Procedure/response rate**  **Population** | Cross sectional study.  To address physicians’ knowledge epidemiology and biostatistics.  A questionnaire was developed and tested on a small group of faculty graduate students. The survey population consisted of 229 individuals being internal medicine staff at an affiliated teaching hospital in the USA. Response rate was 63%.  Participants were identified as fulltime academic (n=62), practicing (n=117) or house staff (n=50.) Respondents =141. |
| **Type of questionnaire**  **Questions about inference**  **Type of inference investigated** | Mailed written questionnaire.  The questions and the response alternative are not reported. Authors write that there was one question about p-values and one about hypothesis testing.  NHST |
| **Results/Outcomes** | Results were only reported in this way:  “Question 7, concerning the interpretation of a p value, was correctly answered by only 40 percent of respondents; and question 9, concerning the null hypothesis, was correctly answered by only 50 percent.” |
| **Authors result/conclusion** | From discussion: “Comparison of the questionnaire responses with the results of the journal review suggests that physicians may not be prepared adequately to evaluate medical literature.” |
| **Methodological comments**  **Other comments** | It is difficult assess and value the results as authors do not describe the questions or response alternatives. |

| **Author**  **Year**  **Country** | Westover  2011  US |
| --- | --- |
| **Study design**  **Purpose**  **Procedure/response rate**  **Population** | Cross sectional study, Quiz  Test probabilistic logic.  A Quiz was administered to a group of 246 physicians at three major US teaching hospitals. Participants were presented with a vignette of study results. |
| **Type of questionnaire**  **Questions about inference**  **Type of inference investigated** | Quiz  Consider a typical medical research study, for example designed to test the efficacy of a drug, in which a null hypothesis H0 (’no effect’) is tested against an alternative hypothesis H1 (’some effect’). Suppose that the study results pass a test of statistical significance (that is P-value <0.05) in favor of H1. What has been shown?  1. H0 is false.  2. H1 is true.  3. H0 is probably false.  4. H1 is probably true.  5. Both (1) and (2).  6. Both (3) and (4).  7. None of the above. |
| **Results/Outcomes** | Answer (1) (2) (3) (4) (5) (6) **(7)** (Correct response is 7, in bold)  Number 8 0 58 37 6 69 **12**  Percent 4.2 0 30.5 19.5 3.2 36.3 **6.3** |

| **Authors result/conclusion** | From abstract:  Unfortunately, most physicians misunderstand the underlying probabilistic logic of significance tests and consequently often misinterpret their results. This near-universal misunderstanding is highlighted by means of a simple quiz which we administered to 246 physicians at two major academic hospitals, on which the proportion of incorrect responses exceeded 90%. |
| --- | --- |
| **Methodological comments**  **Other comments** | Article is an essay of the logic and mistakes of NHST. |

| **Author**  **Year**  **Country** | Wulff et al  1987  Denmark |
| --- | --- |
| **Study design**  **Purpose**  **Procedure/response rate**  **Population** | Cross sectional survey, multiple choice test  To assess knowledge of elementary statistical expressions.  A random selection of 250 Danish doctors (in March 1983) was contacted and asked to anonymously respond to a multiple-choice questionnaire. 140 (response rate 59%) responded and 97 participants of clinical research courses.  Respondents were 1) general practitioners (24%) other specialists (16%), 50% were junior hospital doctors and 8% were others). This corresponded well to all Danish doctors according to authors, and 2) 97 participants of two introductory courses in clinical research methods and biostatistics at the start of these courses. |
| **Type of questionnaire**  **Questions about inference**  **Type of inference investigated** | Written questionnaire  There were 9 questions about statistical expressions, 3 regarded interpretations of p-values and statistically significant findings.  A  A controlled trial of a new treatment led to the conclusion that it is significantly better than  placebo: p < 0.05. Which of the following statements do you prefer?  (1) It has been proved that the treatment is better than placebo.  (2) If the treatment is not effective, there is less than a 5 per cent chance of obtaining such results.  (3) The observed effect of the treatment is so large that there is less than a 5 per cent chance that the treatment is no better than placebo.  (4) I do not really know what a p-value is and do not want to guess.  B  A research team wishes to examine whether or not the ingestion of licorice decreases the plasma concentration of magnesium. Twenty-three volunteers ingest a considerable amount of licorice, and no significant change in the serum magnesium is found (p > 0.05). Which of the following  statements do you prefer?  (1) There is more than a 5 per cent chance of obtaining such results if licorice does not decrease the serum magnesium.  (2) There is only a small probability of obtaining such results if licorice does decrease the serum magnesium.  (3) The research workers ought to have studied more volunteers, then the difference would have become significant.  (4) I do not know what p-values are and do not want to guess.  C  A new drug was tested independently in two randomized controlled trials. The trials appeared comparable and comprised the same number of patients. One trial led to the conclusion that the  drug was effective (p < 0.05), whereas the other trial led to the conclusion that the drug was  ineffective (p > 0.05). The actual p-values were 0-041 and 0.097. Which of the following  interpretations do you prefer?  (1) The first trial gave a false-positive result.  (2) The second trial gave a false-negative result.  (3) Obviously, the trials were not comparable after all.  (4) One must not attach too much importance to small differences between p-values.  (5) I do not understand the problem and do not wish to guess.  NHST |
| **Results/Outcomes** | Correct answers are A (2), B (1) and C (4).  Answers given by doctors and participants of research courses, proportions. Bold indicates correct response.  A  1) 20% - 6%  **2) 13% - 39% (correct)**  3) 51% - 54%  4) 16% - 1%  B  1**) 39% - 67% (correct)**  2) 29% - 26%  3) 13% - 5%  4) 18% - 2%  C   1) 2% - 6% 2) 3% - 3% 3) 41% - 34% 4) **34% - 43% (correct)** 5) 20% - 14% |
| **Conclusion** | Authors conclusion: It is concluded that the statistical knowledge of most doctors is so limited that they cannot be expected to draw the right conclusions from those statistical analyses which are found in papers in medical journals. |
| **Methodological comments**  **Other comments** |  |

## Studies about inferences of results from confidence intervals (and results from null hypothesis significance testing)

| **Author**  **Year**  **Country** | [Alothman](https://www.scirp.org/journal/articles?searchcode=Hella+O.++Alothman&searchfield=authors&page=1) et al  2020  Saudi Arabia |
| --- | --- |
| **Study design**  **Purpose**  **Procedure/response rate**  **Population** | Cross sectional design, online survey  To study some aspects of the understanding of graphically presented CIs and SE bars by authors of articles published in international journals. We investigated the interpretation of error bars in relation to statistical significance.  In 2001 and 2002 emails were sent to 3944 authors of research articles published in leading journals in the three disciplines. Response rate 15.2%.  The population consisted of a convenient sample of 440 participants (program directors, trainers, and residents, general practitioner) working in one out of six governmental hospitals in Riyadh, Saudi Arabia. 51.1% of responders were women. More than half of responders resported having conducted research as a co-author and 38% as a principal investigator. 5.9% reported having a master or PhD degree. |
| **Type of questionnaire**  **Questions about inference**  **Type of inference investigated** | Online questionnaire.  Out of 24 questions one was about the confidence level of confidence intervals and two questions were about NHST and the other about the definition of the p-value  Question about confidence interval:  Which one of Confidence interval is the widest:  a) 90%  b) 95%  c) 98%  d) 99%  Question about hypothesis testing using p-value:  If you reject the null hypothesis at 10% then:  a) It will be rejected at 5%  b) It could be rejected or accepted at 5%  c) It can’t be rejected at 5% |
| **Results/Outcomes** | Results are extracted from figure 2, which displays the proportion of correct answers in brackets (according to authors).  Statements (correct answer, according to authors, in bracket) with proportions correct answers:   - Which one of Confidence intervals is the widest (99%)   41% correct answers   - If you reject the null hypothesis at 10% then (it could be rejected or accepted at 5%) 66% correct answers - The p-value means (Probability to reject the null hypothesis when its true)   43% correct answers (according to authors) |
|  | Authors conclude: Physicians were found to have sufficient information about the basic principles of statistics which are commonly used in medical research articles. |
| **Methodological comments**  **Other comments** | Authors do not seem to understand the principles of NHST. The question about hypothesis testing using p-value does not make sense as the significance level should be set in advance of the test. Also, the definition of the p-values is a definition of the significance level. |

| **Author**  **Year**  **Country** | [Belia](https://www.frontiersin.org/people/u/191756) et al.  2005  Australia |
| --- | --- |
| **Study design**  **Purpose**  **Procedure/response rate**  **Population** | Online survey, experimental website.  To study some aspects of the understanding of graphically presented CIs and SE bars by authors of articles published in international journals. We investigated the interpretation of error bars in relation to statistical significance.  In 2001 and 2002 emails were sent to 3944 authors of research articles published in leading journals in the three disciplines. Response rate 15.2%.  473 authors who had published empirical articles in 1999-2001 in psychology, behavioral neuroscience or medicine. |
| **Type of questionnaire**  **Questions about inference**  **Type of inference investigated** | Experimental website where respondents were asked to move a group mean with an error bar (CI or SE) until groups different significantly.  Respondents saw either 1) (CI group) a display with a group 1 mean with 95% CI and were asked to click to move the group 2 mean with attached 95% CI up or down, until the two means were judged to be just statistically significantly different (p<.05, two-tailed), or 2) (SE group) same display except mean and SE bars for group 1 and 2, or 3) (RM, repeated measure group) a display with SE bars of means labeled pretest and posttest, that is as in repeated measures of same group.  Inferences from comparison of intervals on the two separate means with CI, SE bars or SE bars for repeated measures. |
| **Results/Outcomes** | Wide spread of responses across CI, SE and RM groups presented in graph. A sizable anchoring effect across groups. Respondents to our CI task were generally too strict: They tended to set the means too far apart, not realizing that the .05 statistical significance borderline requires overlap |
| **Authors result/conclusion** | We conclude that very many researchers whose articles have appeared in leading journals in psychology, behavioral neuroscience, and medicine have fundamental and severe misconceptions about how CIs and SE bars can justifiably be used to support inferences from data.  We identified four different problems.   - First, responses were very widely spread and inaccurate. - Second, respondents overall (78%) did not adequately distinguish CIs and SE bars. - Third, 31.5 % of the respondents used the incorrect rule that error bars, whether a 95% CI or SE bars, just touch when means are just statistically significantly different (p .05). - Forth, for RM task, a large majority in every discipline apparently overlooked the clear statements that the means they saw were from a repeated measures or paired design. |
| **Methodological comments**  **Other comments** | Relevant population. Poor response rate. Complex question. |

| **Author**  **Year**  **Country** | Best  2013  USA |
| --- | --- |
| **Study design**  **Purpose**  **Procedure/response rate**  **Population** | Cross sectional questionnaire  To test dental residents’ knowledge of biostatistics. There were also other aims.  All 106 program directors of resident’s programs for Oral and Maxillofacial Surgeons were contacted and asked to forward an email to residents and to ask them to respond. 112 of the estimated total of 900 responded, making the response rate about 12.4%  82% were male. |
| **Type of questionnaire**  **Questions about inference**  **Type of inference investigated** | Online survey  The questionnaire was the same as used by Windish. Participants were asked to rate their attitude towards statistics and their confidence in statistics and were also asked knowledge question. One of the knowledge questions was phrased using a vignette:  “In a placebo-controlled trial of the use of a chlorhexidine rinse to prevent alveolar osteitis (dry socket) after third molar extraction, 8% of patients receiving the treatment had dry socket and 9% of patients receiving the placebo had dry socket. In reporting this finding, the authors stated that P > .05. This means: “   1) The chances are greater than 1 in 20 that a difference would be found again if the study were repeated. 2) The probability is less than 1 in 20 that a difference this large could occur by chance alone. 3) The probability is greater than 1 in 20 that a difference this large could occur by chance alone. 4) The chance is 95% that the study is correct. |
| **Results/Outcomes** | Alternative 3 is correct.  The results of the p-value question is poorly described, but authors report that:  “Only 46% correctly identified that a P .05 (not statistically significant) indicates at least a 1-in-20 chance occurrence.”  And  “Of those who expressed “complete confidence” in interpreting a P value, 8 performed the knowledge task correctly and 7 performed it incorrectly” |
| **Conclusion** | Authors overall conclusion was that:  “OMS residents lack knowledge in biostatistics and the interpretation of research and are thus unprepared to interpret the results of published clinical research” |
| **Methodological comments**  **Other comments** |  |

| **Author**  **Year**  **Country** | Bezzina et al.  2014  Malta and United Kingdom |
| --- | --- |
| **Study design**  **Purpose**  **Procedure/response rate**  **Population** | Online survey using a survey tool.  Purpose: to study if statistical misconceptions pervasive among academics with a special interest in business research methods?  The target population consisted of academics who are members of the Research Methodology Special Interest Groups of either the British Academy of Management or  The European Academy of Management or have attended the European Conference on Research Methodology at least once in the past three year, estimated total of 679 potential respondents after accounting for multiple list membership. Emailed invitation. Response rate; 11.8% returned complete responses.  Mostly academics with document interest in research methodology: male (51.2%), having doctoral degree (70.0%), from United Kingdom (53.8%). |
| **Type of questionnaire**  **Questions about inference**  **Type of inference investigated** | The main questionnaire consisted of 30 randomly ordered categorical statements representing statistical mis/conceptions. Respondents were requested to tick one from ‘agree’, ‘disagree’ or ‘don’t know’.  There was one statement about the p-value:  “The p-value is the probability that the null hypothesis H0 is true”  And there was one statement about confidence intervals: “A confidence interval is a statement about the unknown population parameter”. |
| **Results** | 32% wrongly agreed that “The p-value is the probability that the null hypothesis H0 is true”  8% wrongly (according to authors, see comment below) disagreed that “A confidence interval is a statement about the unknown population parameter” |
| **Authors result/conclusion** | General: The findings of this study suggest both a lack of knowledge and a high pervasiveness of statistical misconceptions among academics with a special interest in business research methods. |
| **Methodological comments**  **Other comments** | Relevant population. Low response rate.  According to confidence interval theory, confidence intervals derived over unlimited repetitions of valid tests /analyses (the frequentist perspective) will contain the true parameter with a frequency equal the confidence level. Thus, the confidential interval procedure provides statements about the long-term coverage of the unknown true population parameter. An observed confidence interval is then not a statement about the unknown population parameter. |

| **Author**  **Year**  **Country** | Coulson et al.  2010  Australia |
| --- | --- |
| **Study design**  **Purpose**  **Procedure/response rate**  **Population** | Emailed survey to two separate populations investigating two experiments, one comparing inferences of CIs in figures and text vs results from NHSTs as figures and text. The other experiment, post hoc designed, assessed ratings of agreements of inferences as in the first experiment.  Comparison of interpretation of results presented using null hypothesis significance testing (NHST), or confidence intervals (CIs) and to assess agreements.  2 322 emails were sent (190 emails (8.2%) bounced) to researchers who had published in psychology, behavioral neuroscience, and medical journals were asked by email to interpret the results of our two fictitious studies. Response rate 15.5% of 2,132 assumed received.  In the other experiment 640 emails were sent and 50 (8.8%) elicited usable results.  Total n=330.  Not described further. |
| **Type of questionnaire**  **Questions about inference**  **Type of inference investigated** | Emailed survey. We asked respondents to interpret two similar studies presenting results in different ways. One gave a statistically significant (p = 0.02), and the other a non-significant (p = 0.22) result in the same direction. Results were shown as CI figure, CI in text, NHST figure or NHST text. The data were the same in every case.  Authors were asked, first, to answer in their own words “what do you feel is the main conclusion suggested by these studies and were then asked to rate agreement from 1 (strongly disagree) to 7 (strongly agree) about the results in the two studies 1) being consistent, 2) strong evidence of one being more effective and 3) conflict about effectiveness.  Comparison of inference of CI and NHST in terms of consistency, effect and conflict about effect. |
| **Results** | Several comparisons of assessments of consistency and effectiveness. |
| **Authors result/conclusion** | Text responses to the initial question were very diverse. Many gave evidence of misconception, especially about p values or NHST.  General: Responses from 330 authors varied greatly, but interpretation was generally poor, whether results were presented as CIs or using NHST. Confidence intervals permit, but do not guarantee, better inference than statistical significance testing.  Regarding CI:s When interpreting CIs, respondents did much better – gave much more justifiable ratings – if they avoided any reference to NHST and thought in terms of the intervals and the relation between the two intervals presented in the CI figure format |
| **Methodological comments**  **Other comments** | Relevant population. Low response rate. Complex assessment. |

| **Author**  **Year**  **Country** | Fidler (thesis)  2005  Australia |
| --- | --- |
| **Study design**  **Purpose**  **Procedure/response rate**  **Population** | Surveys part of thesis about statistical inference.  Elucidate various misconceptions of inference.  Surveys directed to university students.  First survey: 79 final year Batcher or Master students in environmental science classes.  Second survey: 55 second year ecology students.  Third survey: 177 first year psychology students (at end of long introductory statistic course)  Fourth survey: 180 undergraduate students who had completed between one and four semesters of statistics. |
| **Type of questionnaire**  **Questions about inference**  **Type of inference investigated** | Four surveys  First survey: scenarios of where students were asked to rate whether there was strong, moderate, equivocal evidence for or against the support of an effect given different ways (CI or NHST) to present results.  Second survey: replication of survey one with within subject’s design.  Third survey: examined students’ interpretation of results presented as either p values or Cis.  Fourth survey: investigated definitional understanding of CIs, that is, understanding the definition of a CI, that it is inferential and what it estimates. Second, relational understanding of CIs, that is, how the various determinants of a CI affect each other (e.g., that a CI gets wider as the confidence level increases).  Various aspects. |
| **Results/Outcomes** | First survey, (from authors text): 61% of students did not demonstrate the misconception that statistical non-significance means ‘no effect’ when given results presented in the NHST format.  …Of the students who demonstrated the misconception in the NHST scenarios, an overwhelming majority (87%) gave correct answers to the CI scenarios.  Second survey, (from authors text): This within-groups replication of the survey in part one provided even stronger evidence that CIs have a cognitive advantage over p values.  Third survey, (from authors text): Many students failed to identify the CI as an inferential statistic, instead interpreting it as descriptive statistic—either bizarrely interpreting it as an estimate of the sample mean, or as something equivalent to the range (or truncated) range of individual scores.  Fourth survey. Many students presented misunderstandings of Cis as descriptive intervals as well as Cis relational properties, for example to confidence level and sample size. |
| **Authors result/conclusion** | In part one, 18% of students (who provided answers directly addressing the CI) spontaneously generated the misconception that a CI estimates the sample mean and 38% the misconception that it estimates the range, or truncated range. Students displayed a limited understanding of how sample size and confidence level relate to CI width. |
| **Methodological comments**  **Other comments** | Relevant populations. Complex assessments.   Difficult to generalize because respondents seem to be students at same faculty, thus, likely to be taught about inferential mistakes. |

| **Author**  **Year**  **Country** | Fidler et al  2009  Australia /USA |
| --- | --- |
| **Study design**  **Purpose**  **Procedure/response rate**  **Population** | Survey in classroom  Let students face different statistical scenarios requiring them to make conclusions based on certain statistical outcomes.  The article reports results of two empirical investigations of the cognitive benefits of Cis. The first experiment investigated whether CI:s reduce the temptation to interpret a non-statistical result as “no effect”. The second experiment was a partly replication of the first as a fully within subject design.    First experiment: 79 Bachelor or master’s students from three separate environmental science classes. Second experiment: 55 second year ecology students. All participants were students at University of Melbourne, Australia. |
| **Type of questionnaire**  **Questions about inference**  **Type of inference investigated** | Written questionnaire.  Students viewed scenarios that required them to make conclusions based on certain statistical outcomes. All were randomly assigned to two of four possible scenarios with fictional data and asked to answer some multiple-choice questions. Students saw one scenario with NHST results, and a second scenario with CI results. The different scenarios involve information about the setting, the null hypothesis, the size of a biologically important effect and were shown low power results as NHTST or CI formats. The were then asked to choose which of five conclusions a researcher would reach:  – There is strong evidence in support of an important effect  – There is moderate evidence in support of an important  effect.  – The evidence is equivocal.  – There is moderate evidence of no effect.  – There is strong evidence of no effect.  Cognitive understandings and beliefs about CI:s |
| **Results** | From results in article:  Experiment 1:  Sixty-one percent (48 of 79, 95% CI: 50 to 71%) of students did not demonstrate the misconception that statistical non-significance means “no effect” when given results presented in the NHST format.  Of those 31 students who demonstrated the misconception in the NHST scenarios, an overwhelming majority (87%; 95% CI: 71%, 95%) answered correctly when they were instead shown CI scenarios.  Experiment 2:  Given the NHST format, 44% (24 of 55; 95% CI: 31%,  57%) of students inappropriately claimed the results as evidence for the null hypothesis. Less than half as many (18%, 10 of 55; 95% CI: 10%, 30%) made this mistake in the CI condition. |
| **Authors result/conclusion** | The results show promise for visually presented CIs and the within-groups replication provided even stronger evidence that CIs have a cognitive advantage over NHST. |
| **Methodological comments**  **Other comments** | Examples based on very specific scenarios about environmental risks |

| **Author**  **Year**  **Country** | Garcia-Perez et al.  2014  Spain |
| --- | --- |
| **Study design**  **Purpose**  **Procedure/response rate**  **Population** | Questionnaire.  To replicate a CI inference study as authors claim the outcome may be biased and to include and test two more interpretations of CI, which the authors claim to be true.  313 individuals enrolled in Bachler program in psychology and 158 individuals enrolled in master program in psychology responded to questionnaire. No response rate reported.  As above. The Bachler program participants were at the time taking an introductory course in statistics. The Master program participants had previously taken a course in inferential statistics. |
| **Type of questionnaire**  **Questions about inference**  **Type of inference investigated** | Written questionnaire, likely.  The questionnaire contained six interpretations of CIs which had been used in a previous publication by Hoekstra et al. A reported result was described as: “the 95% CI for the mean ranges from 0.1 to 0.4” and the statements (all false) were:   1. The probability that the true mean is greater than 0 is at least 95 %. 2. The probability that the true mean equals 0 is smaller than 5 %. 3. The “null hypothesis” that the true mean equals 0 is likely to be incorrect. 4. There is a 95% probability that the true mean lies between 0.1 and 0.4. 5. We can be 95 % confident that the true mean lies between 0.1 and 0.4. 6. If we were to repeat the experiment over and over, then 95 % of the time the true mean falls between 0.1 and 0.4.   In addition, the authors added two more statements, claimed to be correct:   7. “The true mean lies between 0.1 and 0.4” is true with probability 0.95 8. The data are compatible with the notion that the true mean lies between 0.1 and 0.4. |
| **Results/Outcomes** | Number of endorsements of for question 1-6 presented in graphs were 3, 4 and 5 endorsements were most frequent among both first-years students and master students.  For question 7 and 8, both were most endorsed by both groups. |
| **Authors result/conclusion** | From authors text: Our results for a sample of students from a single institution show that misinterpretation of CIs is not as prevalent and widespread as [Hoekstra et al. (2014)](https://www.frontiersin.org/articles/10.3389/fpsyg.2016.01042/full#B6) purported it to be, but this surely varies across institutions according to how statistics is taught in them. |
| **Methodological comments**  **Other comments** | Relevant population. Unknown response rate.  Authors’ two added statements about CIs are claimed to be correct, but they are two ways to attach probability statements to CIs, which is incorrect.  The results in Garca-Perez et al. are broadly consistent with the results in Hoekstra et al. as most respondents endorse false statements about the interpretation of CI. |

| **Author**  **Year**  **Country** | Hayat et al.  2021  United states |
| --- | --- |
| **Study design**  **Purpose**  **Procedure/response rate**  **Population** | Descriptive cross-sectional study, invitations by email.  To assess nursing faculty knowledge of fundamental statistical concepts and their perception of the importance of statistical knowledge in teaching and research. Out of 10 questions about statistical knowledge, one is about inferences.  Invitations sent to 2 151 faculty members from 26 accredited nursing schools; 164 responded (response rate: 7.7%).  Nursing faculty from U.S. nursing schools with accredited research doctorate programs. Participants were female (99.2%), were research doctorate (70.1%), had taken 3 statistical courses or more (70.7%) and were teachers in statistics/biostatistics (16.5%). |
| **Type of questionnaire**  **Questions about inference**  **Type of inference investigated** | Online survey.  Out of 10 questions about statistical knowledge 1 was about the interpretation of a 95% confidence interval. The question was phrased “How would you interpret a 95% confidence interval for the true mean of a numeric health outcome?” |
| **Results/Outcomes** | There were four response alternatives, listed below   a) You can be 5% confident that the interval will not include the true mean b) You can be 95% confident that the interval will include the true mean. c) If you draw repeated random samples and calculate a confidence interval for each, you can expect 95% of the intervals to contain the true mean. d) I do not understand the expression and do not want to guess.   The correct response, c, was selected by 42.7% (77) of responders. |
| **Authors result/conclusion** | From abstract: “Problematic concepts included randomization (43.3% correct), and interpreting a confidence interval (42.7%) and odds ratio (33.5%). |
| **Methodological comments**  **Other comments** | Low response rate |

| **Author**  **Year**  **Country** | Hoekstra et al.  2012  Netherlands |
| --- | --- |
| **Study design**  **Purpose**  **Procedure/response rate**  **Population** | Cross sectional questionnaire study.  To test how CIs and results based on NHST are interpreted regarding confidence in the alternative hypothesis as well as compare differences in interpretational mistakes.  PhD students working at eight different psychology departments in the Netherlands were invited by email to participate and were sent eight scenarios to assess and were asked to perform the tasks within two weeks. Response rate 44%.  66 participants, PhD students with a completed master’s degree (mean age 27 years, 67% women). |
| **Type of questionnaire**  **Questions about inference**  **Type of inference investigated** | Written scenarios, task to be performed and returned within two weeks.  Respondents were sent eight scenarios: four using CIs as presentation modes and four using conventional NHST with a mean difference, t-value and p-value. The results presented in the CI scenarios were results identical to those in the NHST scenarios, except for a multiplication factor. The p values were chosen to be close to the usually chosen significance level of .05 (.04 and .06), or clearly above or below this level (.02 or .13)  Participants were asked two types of questions per scenario: confidence in and likelihood of replication. For confidence in Ha estimates, the question was:  ‘”What would you say the probability is that there is an effect in the expected direction in the population based on these results?”  For replicability, the question was:  ‘‘What would you say the probability is that you would find a significant effect if you were to do the same study again?’’  The authors hypothesised there was a stronger “cliff effect”, i.e. tendency to dichotomize results, when presented NHST compared to CI scenarios.  The question about inference was:  ‘‘What do these results tell you about the situation in the population? Explain how you came to your conclusion.’’  Confidence, replication, inferences |
| **Results/Outcomes** | Differences in estimates regarding “confidence” and replications was compared across scenarios illustrated in figures. |
| **Authors result/conclusion** | From abstract:  Fewer misinterpretations of statistics—such as accepting the null hypothesis—and more references to effect size were found when results were presented as CIs. Furthermore, participants tended to be more certain about the existence of a population effect in the expected direction and about the replicability of the results when the results were presented following the conventions of NHST than when presented using CIs. Contrary to expectations, no evidence of a more precipitous drop in the belief of the existence of a population effect and replicability estimates when p values exceeded the significance level of .05 was found when data were presented using NHST instead of by CIs. |
| Methodological comments  Other comments | Relevant population. Fair response rate. Quite complex intervention.  Compares inferences of CIs and NHSTs, but in a frequentist view there are no correct answer to the questions asked. |

| **Author**  **Year**  **Country** | Hoekstra et al.  2014  Netherlands |
| --- | --- |
| **Study design**  **Purpose**  **Procedure/response rate**  **Population** | Paper survey.  To investigate the extent to which CIs are misinterpreted by researchers and  students, and to investigate to what extent any misinterpretations are reduced by experience in research.  University students at different levels and researchers at psychology departments were asked to take paper survey about inferences of CIs. Almost all agreed to participate and fulfilled the task.  A sample of bachelor students (n=442) master students (n=34) from the University of Amsterdam and researchers (n=120) from three Dutch universities. |
| **Type of questionnaire**  **Questions about inference**  **Type of inference investigated** | Questionnaire  Participants were presented a fictitious scenario of a professor who conducts an  experiment and reports a 95 % CI for the mean that ranges from 0.1 to 0.4. Respondents were then asked to look at six inferential statements (all of which were false) and check boxes whether each one was either true or false. The six statements were:   1. The probability that the true mean is greater than 0 is at least 95 %. 2. The probability that the true mean equals 0 is smaller than5 %. 3. The “null hypothesis” that the true mean equals 0 is likely to be incorrect. 4. There is a 95% probability that the true mean lies between 0.1 and 0.4. 5. We can be 95 % confident that the true mean lies between 0.1 and 0.4. 6. If we were to repeat the experiment over and over, then 95 % of the time the true mean falls between 0.1 and 0.4.   Inference, replication, confidence |
| **Results/Outcomes** | The mean numbers of items endorsed for first-year students, master students, and researchers were 3.51 (99 % CI = [3.35, 3.68]), 3.24 (99 % CI = [2.40, 4.07]), and 3.45 (99 % CI = [3.08, 3.82]), respectively.  Authors investigated correlation between participants’ self-indicated level of experience (on a scale 0-10) and number of items (falsely) endorsed. The correlation between endorsed items and experience was slightly positive (0.04; 99 % CI = [−0.20; 0.27]), contrary to what  one would expect if experience decreased the number of misinterpretations. |
| **Authors result/conclusion** | From abstract:  Although all six statements were false, both researchers and students endorsed, on average, more than three statements, indicating a gross misunderstanding of CIs.  Self-declared experience with statistics was not related to researchers’ performance, and, even more surprisingly, researchers hardly outperformed the students, even though the students had not received any education on statistical inference whatsoever. Our findings suggest that many researchers do not know the correct interpretation of a CI. |
| **Methodological comments**  **Other comments** | Relevant population with different educational levels in statistics. Good response rate. High face validity of outcome questions. |

| **Author**  **Year**  **Country** | Kalinowski et al.  2018  Australia |
| --- | --- |
| **Study design**  **Purpose**  **Procedure/response rate**  **Population** | Online survey.  To explore graduate student intuitions about CIs and suggest a tool to improve those  intuitions.  Final year honours undergraduates and graduate students were recruited through official course online noticeboards, email, social networking websites, and word of mouth, 101 students agreed to participate.  101 students. Participants identified their main disciplines as: psychology (66%), social science (13%), neuroscience (6%), and medicine (5%). The remainder (10%) did not identify their discipline |
| **Type of questionnaire**  **Questions about inference**  **Type of inference investigated** | Questionnaire  Respondents were asked to:   - Assess the relative likelihood of points across a CI landing on the population parameter. - Choose the shape that best represented their intuitions about the distribution of a CI - look at 50% CI and were asked to adjust them to correspond to 80% CI, and a 95% CI and conversely repeated to adjust a 95% CI to an 80% and a 50% CI.   Subjective likelihood distributions |
| **Results/Outcomes** | Results are presented of proportion of participants having different kinds of subjective likelihood distributions, including “correct” ones.  Three quarters (75%, [67, 84]) of students adjusted the intervals in the correct direction.  A quarter of students (25%, 25 of 101, 95% CI [16, 33]), incorrectly believed that CI width would decrease as the CI% level increased, that is, they created 95% CIs that were shorter than 80% CIs, and 80% CIs that were shorter than 50% CIs. |
| **Authors result/conclusion** | Shortened form abstract:  An online survey of 101 students found that students’ beliefs about the probability curve within a CI take a variety of shapes, and that in fixed choice tasks, 39% CI [30, 48] of students’ responses deviated from true distributions.  We interpret this as evidence that, for many students, intuitions about CIs distributions are ill-formed, and their responses are highly susceptible to question format. |
| **Methodological comments**  **Other comments** | Relevant population. Unknown validity of outcome questions.  Complex assessment. |

| **Author**  **Year**  **Country** | Lai et al.  2012  Australia |
| --- | --- |
| **Study design**  **Purpose**  **Procedure/response rate**  **Population** | Cross sectional email survey.  Three different surveys to researchers asking them to estimate the variability of replication of p-values.  Email survey to authors of articles in high impact journals in psychology, statistics and medicine published in 2005 to 2007 with 3 different questions.  Response rates included in analysis were as follows:  Survey 1: 7.5 % (136/ 1802) of assumed received email  Survey 2: 5.6 % (84/ 1500) of assumed received email  Survey 3: 8.2 % (140/1715) of assumed received email  Authors of articles in high impact journals. |
| **Type of questionnaire**  **Questions about inference**  **Type of inference investigated** | Email survey  Survey 1  Suppose you conduct a study to compare a Treatment and a Control group, each with size N = 40. A large sample test, based on the normal distribution, of the difference between the two means for your independent groups gives z = 2.33, p = .02 (two-tailed).  Suppose you carry out the experiment a further 10 times, with everything identical but each time with new samples of the same size (each N = 40). Consider what p values you might obtain. Please enter 10 p values that, in your opinion, could plausibly be obtained in this series of replications. (Please, no calculations, and no debate about what model might be appropriate! We are interested in your guesstimate, your intuitions!)  Survey 2 In Survey 2 we asked respondents directly about their estimated prediction interval for replication p, by asking them to estimate first the lower limit, then the upper limit, of the interval they felt has an 80% chance of including replication p.  Survey 3  The procedure was the same as in Survey 1, except for the main questions:  The replication study might reasonably find a p value as low as: p = [ ] Type a value less than .02  The replication study might reasonably find a p value as high as: p = [ ] Type a value more than .02  The chance the p value from the replication study will fall in the interval between my low and high p value estimates above is [ ] %. |
| **Results/Outcomes** | Survey 1  Almost all (133/136; 97.8%) of the subjective p intervals were shorter than the correct interval, meaning that almost all respondents underestimated the variance of replication p.  Survey 2  Most (313/332; 94.3%) of the subjective p intervals were shorter than the correct interval, meaning that most respondents underestimated the variance of replication p.  Survey 3  In most cases (229/279; 82.1%) respondents underestimated the variance of replication p. |
| **Authors result/conclusion** | From abstract:  Results were similar for all three disciplines. Respondents generally found the task unfamiliar and difﬁcult. There was great variability over respondents, but almost all of them gave intervals that were too short. This widespread, and often severe, underestimation of the variability of p may help to explain why researchers place too much interpretive weight on single p values. |
| **Methodological comments**  **Other comments** | Difficult assignment. Very low response rates. |

| **Author**  **Year**  **Country** | Lyu et al.  2018  China |
| --- | --- |
| **Study design**  **Purpose**  **Procedure/response rate**  **Population** | Survey (online and on paper)  To investigate and present raw data available for anyone who is interested in examining how students and researchers misinterpret of P-value and CIs, as well as how NHST and CIs influence the interpretation of research results.  Participants were recruited to an online survey though invitations in social media and data were collected at three different time periods between August 2015 and January 2017. Participants were asked to indicate their field of study. If it was not psychology (or related fields) the survey ended/they were excluded.  Data from a paper and pen survey was collected from participants invited at the 18th National Academic Congress of Psychology, Tianjin, China, in October 2015.    Participants (N = 362, 208 females, 153 males, 1 unanswered, age: 25.65 ± 6.65 years) took part in the survey. Population consisted of undergraduate students, master or junior PhD students, senior PhD students and persons with a PhD. Authors do not state the categorisation frequency. |
| **Type of questionnaire**  **Questions about inference**  **Type of inference investigated** | Online survey + paper and pen survey (for different subpopulations).  The authors present participants to three previously used questions/set of questions to assess of 1) the interpretation of p-value, 2) the interpretation of CIs and 3) how NHST and CIs influence the inference based on results of two Studies.   1) The set questions about p-value were based on Haller and Krauss 2002 as well as Gigerenzer 2004, where participants were presented with the following scenario: Suppose you have a treatment that you suspect may alter performance on a certain task. You compare the means of your control and experimental groups (say 20 subjects in each sample). Further, suppose you use a simple independent means t-test and your result is (t = 2.7, d.f. = 18, p = 0.01). Please mark each of the statements below as “true” or “false”. “False” means that the statement does not follow logically from the above premises. Also note that several or none of the statements may be correct. 2) The set questions regarding inferences of Cis are the same as those used by Hoekstra et al 2014, where participants were presented with the following scenario: Participants were informed that “Professor Bumbledorf conducts an experiment, analyzes the data, and reports: The 95% confidence interval for the mean ranges from 0.1 to 0.4” Please mark each of the statement below as "true" or "false". False means that the statement does not follow logically from Bumbledorf's results. Also note that all, several, or none of the statements may be correct. 3) The questions about how NHST and CI inferences compare was translated from Coulson et al. (2010) and investigated the effect of NHST or CIs on the interpretation of two experiments. In this fictitious scenario, two experiments were conducted to compare a new treatment for insomnia with current treatment. One experiment showed that the mean difference was 3.61, p = 0.02, 95% CI [0.61 6.61], while the other experiment found a mean difference of 2.23, p = 0.22, 95% CI [−1.41 5.87]. Results were shown in four different formats: CI figure, CI text, NHST figure, or NHST text. Participants were randomly assigned to one of four conditions, and were asked to rate their attitude on a scale from 1 (strongly disagree) to 7 (strongly agree) to following statements: “the results of the two studies are broadly consistent” (mentioned as “broadly consistent” below); “there is reasonable evidence the new treatment is more effective” (mentioned as “more effective” below); “there is conflicting evidence about the effectiveness of the new treatment” (mentioned as “conflict” below).   P value (assumption of definite proof, inverse probability, replicability); CI (inference, replication, confidence), NHTS and CI (consistency of results described in different ways) |
| **Results/Outcomes** | Authors do not present results for specific questions (but provide raw data for downloading).  Results are presented in graphs according to educational level categorisations.  Overall, the data indicates that 99% subjects have at least 1 wrong answer of P-value understanding, and 93% subjects have at least 1 wrong answer of CI understanding.  For the statement that the two studies are “broadly consistent,” more respondents agreed when the result presented in CI (CI figure, 34%, CI text, 32%) than in NHST (NHST figure 17%, NHST text 20%). For the statement that the new treatment is “more effective,” more respondents agreed when results were presented in CI (CI figure, 43%, CI with text, 48%) than in NHST (NHST figure 42%, NHST text 29%). For the statement that the two studies are in “conflict,” more respondents agreed when results were presented in NHST (NHST figure 56%, NHST text 49%) than in CI (CI figure, 35%, CI text, 48%). |
| **Authors result/conclusion** | Authors do not themselves comment on the results but provides their data set for educational purposes. |
| **Methodological comments**  **Other comments** | Relevant population. Use of set of inference questions that has been used previously. The authors translations are not reported in the article but could be found in a codebook in a supplement available at the journal website.    Overall results broadly consistent with previous studies using same set of outcome questions. |

| **Author**  **Year**  **Country** | Lyu et al.  2020  China |
| --- | --- |
| **Study design**  **Purpose**  **Procedure/response rate**  **Population** | Online survey  Investigate misunderstandings of p-values and confidence intervals using questions form previously reported studies.    A total of 4,206 respondents from different backgrounds (respondents’ academic background was based on the degree awarded in China) voluntarily participated in the survey. However, 2,727 of them withdrew before completing the survey, leaving a sample size of 1,479 (responders 35.0%). Data were collected from September 2017 to November 2018.  1479 persons in various scientific fields and with different scientific degrees. |
| **Type of questionnaire**  **Questions about inference**  **Type of inference investigated** | Online survey + paper and pen survey (for different subpopulations). The survey included scenarios on p values and CIs.  There were questions about p-values and CIs adopted from previously used research.  P-values  Questions about p-values was adopted from previously used publications (by Haller and Krauss 2002, Gigerenzer 2004, Lyu 2018). To investigate the understandings of non-significant results, two versions of the survey were created: one used a significant scenario (p < .05 and CIs did not include zero) and the other used a non-significant scenario (i.e., p > .05 and CIs included zero). Participants were randomly assigned to the significant and non-significant version. Participants were also asked to indicate their confidence in their judgments (from 1-5, increasingly confident).  Participants were presented with the following scenario:  Suppose you have a treatment that you suspect may alter performance on a certain task. You compare the means of your control and experimental groups (50 subjects in each sample). Your hypotheses are as follows. H0: No significant difference exists between the experimental and the control groups. H1: Significant difference exists between the experimental and the control groups. Further, suppose you use a simple independent means t test, and your result is t = 2.7, df = 98, p = .008 (in the significant version) or t = 1.26, df = 98, p = .21 (in the non-significant version)  Please mark each of the statements below as “true” or “false”. “False” means that the statement does not follow logically from the above premises. Also note that several or none of the statements may be correct.  CIs  The set questions regarding inferences of CIs were similar to those used by Hoekstra et al 2014 and Lyu2018, where participants were presented with the following scenario: “The 95% confidence interval of the mean difference between the experimental group and the control group ranges from .1 to .4 (or from –.1 to .4 in the non-significant version).”  P value (assumption of definite proof, inverse probability, replicability); CI (Inference, replication, confidence), NHTS and CI (consistency of results described in different ways) |
| **Results/Outcomes** | The results showed that 89% of respondents had at least one error when interpreting a p value, and 93% of respondents committed at least one error when interpreting CIs. The percentage of misinterpretation failed to show differences across educational attainment or academic background. For the difference between the significant and the nonsignificant versions, the error rate for p values was lower in the latter (86%) than in the former (92%). This study failed to find strong evidence for the difference between significant CIs (94%) and non-significant CIs (91%).  Results -p-value  Proportion endorsing the statement (significant; non-significant version). Note that statements were formulated somewhat differently in significant and non-significant versions, below significant version.   - You have absolutely disproved the null hypothesis. (53%; 53%) - You have found the probability of the null hypothesis being true. (51%; 45%) - You know, if you decide to reject the null hypothesis, the probability that you are making the wrong decision. (65%; 60%) - You have a reliable experimental finding in the sense that if, hypothetically, the experiment was repeated a great number of times, you would obtain a significant result on 99% of occasions (55%; 42%).   Results about CIs  Proportion endorsing the statement (significant; non-significant version). Note that statements were formulated somewhat differently in significant and non-significant versions, below significant version)   - There is a 95% probability that the true mean lies between .1 and .4. (56%; 58%) - If we were to repeat the experiment over and over, then 95% of the time the true mean falls between .1 to .4 (55%; 53%) - If the null hypothesis is that there is no difference between the mean of experimental group and control group, the experiment has disproved the null hypothesis. (48%; 48%) - The null hypothesis is that there is no difference between the mean of experimental group and control group. If you decide to reject the null hypothesis, the probability that you are making the wrong decision is 5% (60%; 54%) |
| **Authors result/conclusion** | The current survey showed that researchers from various fields of science may not be able to correctly interpret p values and CIs. They are unaware of their own misinterpretations. |
| **Methodological comments**  **Other comments** | Relevant population. Use of set of inference questions that have been used previously. Extends previous work by randomly including both statistically significant and non-significant results as vignettes. |

| **Author**  **Year**  **Country** | Susarla et al.  2014  US |
| --- | --- |
| **Study design**  **Purpose**  **Procedure/response rate**  **Population** | Cross sectional survey  The purpose was to assess plastic surgery resident attitudes and knowledge of biostatistics using a pilot survey.  This was a pilot study performed at a local resident institution were 22 respondents (91.7% response rate) completed the questionnaire.  Pilot study: 22 respondents junior and senior residents where 54.5% had taken a formal course in statistics. |
| **Type of questionnaire**  **Questions about inference**  **Type of inference investigated** | Likely written  The survey contained questions about participant´s characteristics and confidence in various statistical concepts. It also contained five vignette knowledge questions, one asking respondents to interpret a p-value and the other to interpret the meaning of a 95% confidence interval in regard of statistical significance.  The question about interpretation of p-values was phrased:  In a randomized, double blind, controlled clinical trial evaluating the use of alloderm for single-stage breast reconstruction, the infection rate among patients who received alloderm reconstruction was 7%, compared to 5% in the control group. The authors report a p-value of 0.06. In interpreting the finding, which of the following is true?   a) The chances are greater than 1 in 20 that a difference would be found again if the study were repeated. b) The probability is less than 1 in 20 that the observed difference in infection rates is due to chance. c) The probability is greater than 1 in 20 that the observed difference in infection is due to chance. d) The study is powered at 94% to detect the observed difference.   The question about the confidence interval was phrased:  In a study looking at the mean mandibular advancement between patients undergoing mandibular distraction versus those undergoing bilateral sagittal split osteotomies, the authors reported a mean mandibular advancement of 11.5 mm in the distraction group and 10 mm in the sagittal group. They calculate a mean difference in advancement of 1.5 mm with a 95% confidence interval of 0.9-2.1. The 95% confidence interval for the true difference in mean advancement between the two populations suggests:   a) There is no statistically significant difference between the two populations. b) There is a statistically significant higher mean advancement in the distraction group. c) There is a statistically significant higher mean advancement in the sagittal split group. d) There is a statistically significant higher mean advancement in the population of patients undergoing distraction. |
| **Results/Outcomes** | Authors do not report answers on p-value question.  Authors report that 45% gave correct answers regarding interpreting confidence interval, but they do not state which answer the consider correct. |
| **Authors result/conclusion** | Plastic surgery residents place a high degree of importance on knowledge of biostatistics in the practice of plastic surgery but have only a fair understanding of core statistical concepts. |
| **Methodological comments**  **Other comments** | Question about confidence intervals is about using it making claims about statistical significance. |

| **Author**  **Year**  **Country** | Susarla et al.  2016  US |
| --- | --- |
| **Study design**  **Purpose**  **Procedure/response rate**  **Population** | Cross sectional survey  The purpose was to assess plastic surgery resident attitudes and knowledge of biostatistics using a national survey.  An invitation to participate in the online survey was emailed to residency program directors in the US, who subsequently distributed the survey to their plastic surgery residents. The authors report the response rate to be 12.3%.  The study population of 123 responders were junior (62.6%) or senior (37.4) residents and 58.5% reported having taken formal coursework’s in statistics. Age and sex of participants was not reported. |
| **Type of questionnaire**  **Questions about inference**  **Type of inference investigated** | Online survey  The survey contained questions about participant´s characteristics and confidence in various statistical concepts. It also contained five vignette knowledge questions, one asking respondents to interpret the meaning of a 95% confidence interval in regard of statistical significance.  The question about interpreting a confidence interval was phrased:  In a study looking at the mean mandibular advancement between patients undergoing mandibular distraction versus those undergoing bilateral sagittal split osteotomies, the authors reported a mean mandibular advancement of 11.5 mm in the distraction group and 10 mm in the sagittal group. They calculate a mean difference in advancement of 1.5 mm with a 95% confidence interval of 0.9-2.1. The 95% confidence interval for the true difference in mean advancement between the two populations suggests:   e) There is no statistically significant difference between the two populations. f) There is a statistically significant higher mean advancement in the distraction group. g) There is a statistically significant higher mean advancement in the sagittal split group. h) There is a statistically significant higher mean advancement in the population of patients undergoing distraction. |
| **Results/Outcomes** | Proportion of respondents answering correct in question about confidence intervals: 26.0%). Authors do, however, not state themselves which answer they consider is correct. |
| **Authors result/conclusion** | Plastic surgery residents place a high degree of importance on knowledge of biostatistics in the practice of plastic surgery but have only a fair understanding of core statistical concepts. |
| **Methodological comments**  **Other comments** | Question about confidence intervals is about using it making claims about statistical significance. |

| **Author**  **Year**  **Country** | Windish et al.  2007  US |
| --- | --- |
| **Study design**  **Purpose**  **Procedure/response rate**  **Population** | Cross sectional, written survey  To assess assessment of residents' biostatistics knowledge and interpretation of study results using a new instrument developed for this purpose.  Residencies at all postgraduate levels at both universities affiliated and community-based programmes in Connecticut were invited to participate. Of available residents, at the time, 277 (75.5%) completed the survey. Response rates for individual programmes ranged from 28.1% to 80%.  The study population (n=277) consisted of 48% women. Most had no advanced degree; 4.0% had a PhD and 10.2 had a master’s degree. |
| **Type of questionnaire**  **Questions about inference**  **Type of inference investigated** | Written questionnaire which was administered during the first 25 minutes of an inpatient conference lecture for current residents. The questionnaire had for sets of question in which one was about biostatistics knowledge. There were two questions about inference, one on the inference of a non-significant p-value and one about a 95% confidence interval and statistical significance. The question about interpreting a p-value>0.05: In a placebo-controlled trial of the use of aspirin and dipyridamole to prevent arterial restenosis after coronary angioplasty, 38%, of patients receiving the treatment had restenosis, and 39% of patients receiving placebo had restenosis. In reporting this finding, the authors stated that P >.05. This means: a. The chances are greater than 1 in 20 that a difference would be found again if the study were repeated. b. The probability is less than 1 in 20 that a difference this large could occur by chance alone. c. The probability is greater than 1 in 20 that a difference this large could occur by chance alone. d. The chance is 95% that the study is correct. The correct answer is c. The question about interpreting a 95% confidence interval and statistical significance. Researchers measure cholesterol levels in a sample of patients in New Zealand and Asia and find the following results: Region \| Sample Size \| Mean Cholesterol Level (mmoVL) \| Standard Deviation (mmol/L); New Zealand \| 100 \| 5.4 \| 1.2; Asia \| 150 \| 4.9 \| 1.3 They calculate the mean and 95% Cl for the true difference in mean cholesterol levels between the 2 populations and find: Mean Difference 0.5 mmol/L and 95% Cl (0.18-0.82). a) The 95% confidence interval for the true difference in mean cholesterol levels between 2 populations suggests that: there is no statistically significant difference in mean cholesterol levels between the 2 populations. b) There isa statistically significant higher mean cholesterol level in the Asian population as compared to the New Zealand population. c) There is a statistically significant higher mean cholesterol level in the New Zealand sample as compared to the Asian sample. d) There is a statistically significant higher mean cholesterol level in the New Zealand population as compared to the Asian population. The correct answer is d. |
| **Results/Outcomes** | Correct responses about the interpreting the meaning of p>0.05 was given by 58.8%; 95% CI (53.0 to 64.6)  Correct responses about the inference of a 95% confidence interval and statistical significance were given by 11.9%, 95% (8.0 to 15.7) |
| **Authors result/conclusion** | Most residents in this study lacked the knowledge in biostatistics needed to interpret many of the results in published clinical research. |
| **Methodological comments**  **Other comments** | Question about confidence intervals is about using it for hypothesis testing. |
