# Supplementary material for: Inferences of p-values, statistically significant results, and confidence intervals – A systematic review of empirical studies
Source: PLoS One. 2026 Nov 11;21(11):e0362071. doi: 10.1371/journal.pone.0362071 (PMC13754157; doi:10.1371/journal.pone.0362071)
Supplement: Supplementary file 4 [file pone.0362071.s003.docx]

Supplement 3

Detailed text section of results

**Results**

We identified 6,300 unique references of which 55 studies from the year 1963 and forward were included, see Flow chart in figure 1. Studies were performed in different regions of the world: mostly in European countries, the United States, Australia and China, but there were also studies from South America and the Middle East.

Study populations were typically obtained by one of the following: 1) inviting a specific group (such as course participants, a department, a school/university, attendants to a scientific meeting etcetera); 2) contacting and inviting authors, editors or reviewers of scientific publications or 3) by openly inviting persons from a scientific community, such as members of association or a scientific social media group. The total number of participants in all included studies was more than 15,000. In the individual studies the number of participants ranged from 18 to 1,839 people.

The great majority of included studies were cross-sectional surveys using questionnaires for the assessment of inference. More recently published studies usually used online-questionnaires and applications while older studies used written questionnaires, either distributed at a specific occasion, such as at a course or meeting, or sent by mail.

Several different approaches and questions were employed to assess various aspects of inferences related to p-values, null hypothesis significance testing, and confidence intervals. Some instruments and questions have been used repeatedly, either in their original form or in variations, suggesting that those approaches are seen as relevant to many and at that the questions and instruments have a fair and direct face validity. Such instruments often use questions with true or false response options about what logically can be deduced from the presented information. Other questions ask participants about their belief or confidence in a presented statement or research output, which on an aggregated level represents the subjective view of the respondents.

Some studies used questions and instruments that appear complex and difficult to understand, requiring respondents to assess or interpret results without having adequate or even sufficient information. There are examples of authors using questions which are unclear, not providing any correct response alternative and even erroneously claiming that a certain response alternative is correct, despite it being wrong. We present the ambiguities and controversies that we have found under a specific heading last in the result section.

Overall, there was substantial heterogeneity in how inference have been investigated in empirical studies. Below we summarize and present the approaches and results according to different themes, arranged in chronological order of their emergence by publication time. Characteristics of included studies are presented in Table 1. A detailed presentation of each included study, the study population, the way the investigation of inferences was performed, its results and authors main conclusions are available in S2 Appendix.

**Assessments the degree of belief or confidence in research findings**

The first study included was published in the early 1960s by Rosenthal et al. [1]. It explored inference among psychology researchers (nine faculty members with a PhD and 10 graduate students) by examining the relationship between different p-value levels and the “degree of belief in research findings as a function of associated p levels”. Based on a written survey the respondents rated their belief on a six graded Likert scale for various sized study populations over 14 different p-values. The authors concluded that the degree of belief in findings increased with smaller p-values and larger sample sizes. This study was subsequently replicated by Beauchamp et al. [2] and by Nelson et al. [3]. Beauchamp et al. used a sample of eleven graduate students and nine researchers with PhDs at the psychology faculty, while the sample in Nelson et al. consisted of 85 respondents, with a response rate 29% from a population of authors, editors and APA-members. This latter study also reported seeing a “cliffhanger effect”, where confidence in research findings weakened when the p-value was slightly above conventional thresholds (e.g., .05 and .10). Poitevineau et al. later conducted a similar study on a smaller sample in 2001 and suggested that the .05 cliffhanger effect may be overstated [4].

**Assessments of level of agreement in statistical issues**

Mittag et al. explored the perceptions of American Educational Research Association (AERA) members regarding statistical issues [5]. Data on a 29-item survey, in which they investigated nine clusters of perceptions, was collected using a two-sided one-sheet survey on 225 (21.7% response rate) respondents. The participants rated their agreement on various perceptions regarding null hypothesis significance testing (NHST) and other statistical issues, using a 5-point Likert scale (1 = disagree, 5 = agree). While some items involved true or false statements, others aimed to normatively assess participants' mindsets and viewpoints on statistical concepts. Results were graphically presented as means with 95% confidence intervals. About one third of the questions are about or touches upon inference. The authors reported that the responses were often unsatisfactory, with many participants providing erroneous or illogical answers. Two subsequent studies on researchers in education and psychology using the same approach were conducted by Gordon and Monterde-i-Bort et al. [6, 7]. Monterde-i-Bort et al. compared findings across all three studies and concluded that the results demonstrated a concerning lack of understanding of several fundamental methodological and statistical concepts.

**Interpreting results of NHST**

Many of the studies included in this systematic review investigate inferences about p-values and results from null hypothesis significance testing (NHST) using true or false statements. A statement is true if it follows logically from the premises, and conversely it is false when it does not. Sometimes such statements are presented as straight general principles, for example: *The meaning of a statistically significant finding is that we have proved that the null hypothesis is not true.* In other instances, similar questions and answers are embedded within clinical vignettes to mimic the way results are typically encountered in scientific literature. These approaches have often been used to investigate fallacies—for example, the belief that outcomes from null hypothesis significance testing provide probability measures of a hypothesis being true; that such results can serve as indicators of the likelihood of drawing erroneous conclusions; or that the reliably predict the outcomes of future replications. Below are studies that have investigated inferences using such approaches.

Weiss et al. reported, in a publication from 1980, findings on 141 physicians' knowledge of epidemiology and biostatistics [8]. Although the 10-item questionnaire was not published itself, the authors noted that only 40% of respondents correctly answered a question about p-values, and 50% correctly answered a question on hypothesis testing.

Michael Oakes published results of a short survey—on 70 academic psychologists—in his book *Statistical Inference: A Commentary for the Social Sciences* [9]. Respondents were asked to interpret the results of an independent t-test conducted on a sample of 20 individuals, yielding t=2.7 and p=0.01. They were then asked to assess six statements as true or false, which are summarized in Table 2. All statements were intentionally false, each representing common inferential misunderstanding. Three of the misunderstandings were endorsed as being true by the majority responders (60-86%) and very few answered all six questions correct. The author wrote that the results indicated that 67 of the 70 respondents demonstrated “a less than sound grasp of the conclusions that may properly be drawn from the results of a significance test”. The questions used in this study, or variations of them, have been used in multiple subsequent studies.

**Table 2**

*Three short questionnaires with true or false statements that have been used in several studies to investigate the prevalence of misconceptions of inference from null hypothesis significance testing (Oakes and Falk & Greenbaum) and results presented as confidence interval estimations (Hoekstra et al.) The correct answers to all response alternatives are “False”.*

| **Oakes 1986** | **Falk and Greenbaum 1996** | **Hoekstra et al 2014** |
| --- | --- | --- |
| **Vignette** | **Vignette** | **Vignette** |
| Suppose you have a treatment which you suspect may alter performance on a certain task. You compare the means of your control and experimental group (say 20 subjects in each sample). Further, suppose you use a simple independent t test and your result is (t=2.7, d.f. 18, p=0.01). Please mark each of the following statements below as “true” or “false”. | The subjects were told that a test of significance was conducted, and the result turned out significant for a predetermined level. They were asked what is the meaning of such a result? Subjects were allowed to mark one or more answer as true. | Professor Bumbledorf conducts an experiment, analyzes the data and reports: “The 95% confidence interval for the mean ranges from 01 to 0.4!”  Please mark each of the statements below as “true” or “false”. False means that the statement does not follow logically from Bumbledorf’s result. Also note that all, several, or none of the statements may be correct. |
| **True or false propositions** | **True or false propositions** | **True or false propositions** |
| You have absolutely disproved the null hypothesis (that there is no difference between the population means). | We proved that H_0_ is not true. | The probability that the true mean is greater than 0 is at least 95%. |
| You have found the probability of the null hypothesis being true. | We proved that H_1_ is true. | The probability that the true mean equals 0 is smaller than 5%. |
| You have absolutely proved your experimental hypothesis (that there is a difference between the population means). | We showed that H_0_ is improbable. | The “null hypothesis” that the true mean equals 0 is likely to be incorrect. |
| You can deduce the probability of the experimental hypothesis being true. | We showed that H_1_ is probable. | There is a 95% probability that the true mean lies between 0.1 and 0.4 |
| You know, if you decided to reject the null hypothesis, the probability that you are making the wrong decision. |  | We can be 95% confident that the true mean lies between 0.1 and 0.4 |
| You have a reliable experimental finding in the sense that if, hypothetically, the experiment were repeated a great number of times, you would obtain a significant result on 99% of occasions |  | If we were to repeat the experiment over and over, then 95% of the time the true mean falls between 0.1 and 0.4. |

Some years later Wulff et al. assessed Danish physicians' understanding of statistics, and a year after Scheutz et al., used a similar approach in a sample of Danish dentists and participants in research courses [10, 11]. The questionnaires used included eleven questions, three of which used clinical vignettes to assess respondents' ability to make sound inferences from NHST. Each question offered four or five response options, of which one was correct. There was also a possibility to opt out because not understanding the question and not wanting to guess. The authors of both studies concluded that the statistical knowledge of most physicians and dentists was insufficient to enable them to correctly interpret statistical analyses presented in medical and dental journal articles.

In a minimally described experiment published in 1993, Freeman surveyed 397 doctors, dentists, and medical students in the United Kingdom, asking them to select the most accurate interpretation from four true or false options after reading a vignette describing a statistically significant finding [12]. Only 19% of respondents selected the correct answer, while 15% chose the option to decline answering, citing insufficient understanding of p-values and a reluctance to guess.

Falk et al. used an adapted form of Oakes’s questionnaire and asked 53 psychology students to interpret the meaning of a statistically significant result at a predetermined significance level [13]. The response options were: (1) “we proved that H_0_ is not true”; (2) “we proved that H_1_ is true”; (3) “we showed that H_0_ is improbable”; (4) “we showed that H_1_ is probable”; and (5) “none of answers 1-4 are correct” (see Table 2). Only 13.2% of the study participants correctly identified option (5) as the accurate response.

Ferrill et al. surveyed 707 pharmacists and pharmacy students in the United States about the meaning of a result reported as p<0.05 [14]. Only 13% correctly identified the two accurate interpretations from five options: (1) “There is less than a 5% probability of concluding that there is a difference when none exists,” and (2) “A statistically significant difference was found between the groups tested.”. The study also involved questions about inferring clinical significance and confidence intervals, described below.

Haller et al. administered the same six-question survey as used by Oakes, containing statements representing common statistical misunderstandings (all statements were false), to a German sample of 30 methodology instructors, 39 research psychologists, and 44 psychology students [15]. The proportion of respondents who made at least one error was 80.0% among instructors, 89.7% among psychologists, and 100% among psychology students. The authors concluded that the misconceptions identified by Oakes remained surprisingly prevalent, even among those teaching statistics to psychology students.

In 2007 Windish et al. published an article investigating biostatistical knowledge among 277 medical residents in the United States [16]. The authors had developed and piloted a survey instrument consisting of 20 questions presented alongside clinical vignettes, focusing on various epidemiological and biostatistical concepts that readers may encounter in scientific literature. One question specifically addressed the interpretation of a non-statistically significant result, offering four response options. Another question evaluated the interpretation of a confidence interval (details described under the corresponding heading below). The authors reported that 58.8% of participants selected the correct answer regarding the interpretation of the non-statistically significant result. Overall, the findings in the study indicated that most respondents lacked the necessary biostatistical knowledge to adequately interpret many results presented in published clinical research.

The question regarding NHST inference developed by Windish et al. has been used in several subsequent studies. It was adapted using a different vignette for use in a study by Polychronopoulou et al. who investigated biostatistical knowledge among 127 postgraduate students in orthodontic programs from 10 European countries [17]. The authors reported that only 33% of respondents provided the correct response to the question concerning inferences drawn from a statistically non-significant result. Additionally, the question developed by Windish et al. appears to have been employed in a study by Bookstaver et al., who assessed biostatistical knowledge among resident pharmacists in the United States [18]. Although the authors indicated that they used the same questionnaire as Windish et al., they did not provide the specific questions or the complete questionnaire. Bookstaver et al. reported that 59.6% of the 161 respondents selected the correct answer. Their overall conclusion based on the complete questionnaire was that pharmacy residents' perception and understanding of biostatistics were inadequate. Lastly, Best et al. used a similar question to assess biostatistical knowledge in 112 dental surgeon residents [19]. Authors reported that only 46% identified the correct interpretation of a non-statistically significant finding.

Kalinowski et al. employed an adaptation of Oake's questions in a study involving 120 final-year undergraduate students enrolled in a behavioral science degree program in Australia [20]. The purpose of the study was to assess whether two different teaching interventions could mitigate the inverse probability fallacy - specifically, the misunderstanding that the probability of the observed data given the null hypothesis is equivalent to the probability of the null hypothesis given the observed data. Students rated their level of agreement with various statements using 7-point Likert scales at three time points: once before the intervention and twice afterward. The authors concluded that the inverse probability fallacy was prevalent prior to the interventions, with approximately 82% of students agreeing with at least one statement indicative of this fallacy. Following the interventions, the fallacy was reduced to about 49% and 48% in the respective intervention groups.

In a study conducted Castro Sotos et al., 144 Spanish college students were surveyed regarding common misinterpretations of hypothesis tests and p-values, using variations of Falk et al.'s questions [21]. Participants were also asked to assess their confidence in their knowledge. The results indicated that 56% of respondents correctly answered two questions regarding what NHST indicates about the proof or probability of the null hypothesis and the alternative hypothesis. Additionally, one question asked for the definition of the p-value, providing seven response options, only one of which was correct. The findings revealed that 52% identified the correct answer, while 46% selected the correct option exclusively. The authors concluded that a substantial number of participants harbored misconceptions about NHST inferences and noted that lower levels of self-assessed confidence were associated with a higher prevalence of misconceptions.

Westover et al. published a study, involving 246 physicians at three major teaching hospitals in the United States, using an adapted version of Falk et al.'s survey [22]. This adaptation included seven response alternatives (as opposed to five in the original survey) to address common misconceptions. Among these alternatives, only one was correct, indicating that none of the other six options were valid. This correct response was identified by only 6.3% of respondents.

In a study conducted by Bezzina et al., 80 individuals, primarily academics with a documented interest in research methodology in the field of business management, completed a survey containing 30 statements about statistics [23]. The response options included “agree”, “do not agree”, and “do not know”. Among these statements, two pertained specifically to NHST inference. The results revealed that 32% of respondents incorrectly agreed that the p-value represents the probability that the null hypothesis is true, while 42% mistakenly agreed that statistically significant results indicate strong relationships between variables or substantial differences between compared groups. The authors concluded that these findings suggest both a lack of knowledge and a widespread prevalence of statistical misconceptions among academics with a particular interest in business research methods. There was also a statement about what a confidence interval is, which is presented under that subsequent heading.

Badenes-Ribera et al. investigated four specific inference fallacies in two publications [24, 25]. The fallacies were: the inverse probability fallacy, the replication fallacy, and the magnitude fallacy and the fallacy of clinical significance, and the authors utilized a variation of previously employed questions with true or false response options. The studies were conducted across different populations: one involving a sample of 418 academics in Spain and another comprising 134 Italian and 30 Chilean psychologists. In the Spanish sample, only 6.2% correctly identified five statements regarding the inverse probability fallacy as false, while the corresponding figure in the Italian and Chilean population was 38.4%. The authors also presented findings indicating common misunderstandings related to replication and the implications of NHST results for effect size and clinical significance. They concluded that many academic psychologists struggle to correctly interpret p-values, a finding that is consistent with previous research.

Ferreira et al. reported results from a sample of clinical professionals (physicians, nurses, and others) in Portugal who were asked to evaluate their agreement with four false statements regarding the interpretation of statistically significant findings [26]. Only those who responded that they knew how to interpret “the information provided by the significance (p-value) of a randomized trial” were eligible to answer, thus respondents were a selection of those claiming to master inference. Three of the questions addressed the inverse probability fallacy, while one focused on the replication fallacy. The results demonstrated that misunderstandings were widespread, with only 0% to 3.2% of respondents across the investigated groups correctly identifying all four statements as false. The authors concluded that inferential misinterpretations remain prevalent among health professionals.

Lyu et al conducted a study, published in 2018, involving 362 Chinese students and researchers in the field of psychology, asking them to respond to six true or false questions, all of which were false, regarding the interpretation of statistically significant findings [27]. The results were presented graphically and indicated that between 40% and 60% of the participants endorsed each misunderstanding as true. Additionally, 99% of respondents provided at least one incorrect answer. The same research group replicated their study findings a few years later in a group of 1,479 participants from various scientific fields and backgrounds, recruited from social media groups in China. In this larger sample, the misunderstandings were endorsed as true by 30% to 70% of respondents, and 89% of participants had at least one error [28].

Rajcani et al. conducted a similar study in 2023 involving 65 Slovakian researchers, who were asked to respond to six questions related to misunderstandings about the interpretation of p-values and NHST [29]. The response options included true, false, and an option to opt out if they did not know the answer or did not wish to respond. The study also included questions regarding the interpretation of confidence intervals (described below). The findings revealed that misunderstandings were prevalent, leading the authors to conclude that common misconceptions about NHST and p-values remain widespread among Slovak researchers.

Lytsy et al. published a study in which 75 doctoral students at a medical faculty and 64 statisticians/epidemiologists were asked about the interpretation of statistically significant test results [30]. They investigated whether such results could be considered proof of, or a probability statement about, the truth or falsity of the null or alternative hypothesis, using a question similar to that employed by Falk et al.

Correct inferences to both questions, which is that a statistically significant finding cannot be inferred as either proof or a measure of a hypothesis’ probability, were given by 10.7% of doctoral students and 12.5% of statisticians/epidemiologists. In the latter group, 62.5 % had a PhD (of which 26.6% were in statistics) and over 90% had formal statistical education and worked professionally with biostatistics. Authors concluded that misinterpretation about p-values and statistically significant test results persists also among persons who have substantial statistical education and who work professionally with statistics.

In addition to the studies mentioned above, several other investigations have employed various questions and methodologies to assess different aspects of inferences drawn NHST.

In a study by Vallecillos et al., 436 students from seven distinct fields (Civil Engineering, Computer Science, Pedagogy, Psychology, Medicine, Mathematics, and Business Studies) in Spain were asked whether the statement “A statistical hypothesis test, when properly performed, establishes the truth of one of the two null or alternative hypotheses” was true or false [31]. Data were collected during 1991-1992. The authors reported that 42.7% of respondents incorrectly believed the statement to be true, while only 26.8% identified the correct response. The proportion of misunderstandings varied across different scientific fields. The authors wrote that a noticeable percentage of students had errors of basic aspects referring to the overall logic of the procedure of the hypotheses test.

In a study by Msaouel el al., 153 Greek medical residents responded to a questionnaire comprising seven questions about biostatistics [32]. One of the questions pertained to interpreting a statistically significant finding, offering respondents three true/false alternatives and an option to refrain from answering. The correct response was identified by 26.8% of respondents and authors concluded that a large number of medical residents are unable to correctly interpret crucial statistical concepts. This study also investigated a question about interpreting a confidence interval, which is presented under that heading.

Lecoutre et al. conducted a study in France involving 25 statisticians from pharmaceutical companies and 20 psychological researchers from various laboratories [33]. Participants were presented with four versions of results from a small experiment designed to assess the efficacy of a drug by comparing two groups. The versions varied in terms of effect size (small or large) and statistical significance (significant or not). Two scenarios were designed to conflict: one presented a statistically significant finding of a small effect, while the other reported a non-significant finding of a large effect. Participants were asked to draw conclusions regarding effect size, predict final outcomes if the number of subjects increased, and evaluate the results from an economic perspective. The authors concluded that, overall, respondents displayed a strong inclination to be impressed by statistically significant results; however, interpretations varied between individuals in certain situations.
van den Akker et al. investigated how different sets of experimental results, labeled as “significant” or “non-significant,” influenced the “beliefs in the theory” of researchers and reviewers in the fields of social and experimental psychology [34]. The findings revealed that belief in the theory increased with the number of statistically significant results reported. Furthermore, the results indicated that the effect of a direct replication (using the same method as the original study) on belief was stronger than that of a conceptual replication (using different methods or operationalizations).

In a study by Kühberger et al., 215 psychology students in Austria were presented with descriptions of results from two experiments labeled as either significant or non-significant [35]. Participants were given vignettes that included either statistically significant or non-significant findings and were asked to estimate the expected measures (e.g., sample sizes, means, and standard deviations) that would be reported in the results section of a hypothetical paper. The authors computed effect sizes based on the participants’ ratings to investigate their intuitive understanding of the concept of significance. The results indicated that labeling study results as significant was associated with participants estimating a large effect size, while non-significant results were estimated to have an effect size close to zero.

In a study conducted by Lai et al., authors from leading journals in psychology, medicine, and statistics were invited to participate in an investigation into anticipated and subjective intervals of p-value variability across replications [36]. The authors conducted three surveys: the first asked participants to imagine 10 replications of an initial experiment and to provide 10 plausible p-values for these replications; the second requested an 80% prediction interval for the replication of p; and the third was an extended version of the first survey. A total of 360 respondents provided useful data for analysis. The authors reported that participants generally found the task unfamiliar and challenging, with nearly all respondents providing intervals that were too short.

In a study conducted by Tam et al., a mixed-methods approach was employed to investigate how physicians conceptualize p-values [37]. A total of 247 respondents participated by completing an online survey after being invited through a social media group for general practitioners in Australia. Participants were presented with a dichotomous choice question asking whether it is true following a statistically significant finding that “This means that there is a 5% probability that this result is due to chance alone, or, there is a 95% probability that the conclusion is true.” (Note that the answer is correctly identified as false, by the authors, but note that it is not one, but two statements, which may cause ambiguity). Respondents were also asked to rate their confidence in their answer on a five-point Likert scale. Additionally, participants were prompted to elaborate in a few sentences on what a p-value of 0.05 signifies in the context of an effect presented in a vignette. The authors categorized the responses into six thematic categories. According to their findings, only 29% of respondents answered correctly regarding the meaning of statistical significance. The most frequently identified themes regarding the interpretation of p-values included perceptions of p-values as a real-world probability of a phenomenon (such as the truth or falsehood of a hypothesis), a threshold for determining effect superiority or non-superiority, or simply as an indication of statistical significance, often described in a self-referential manner.

McShane el al., published a study with results from two investigations exploring the influence of p-values on researchers’ interpretations of study findings [38]. The first investigation involved authors publishing in the New England Journal of Medicine, with 75 participants completing an online survey. Respondents were presented with a study summary describing an outcome variable associated with an intervention. The difference was randomly framed as either statistically significant (p = 0.01) or non-significant (p = 0.27). Participants were then asked to select one of four alternatives regarding the effect observed in the sample: greater, lesser, no different, or whether it could not be determined. The primary aim was to assess whether the inclusion of p-value information influenced their conclusions, which it should not – as they were asked to draw conclusions about the sample. The authors reported that participants were significantly more likely to answer correctly when the p-value was set at 0.01 compared to 0.27. They concluded that a focus on statistical significance tends to lead researchers to adopt a dichotomous view of evidence.

In the second investigation, the authors sought to determine whether this observed pattern extends from descriptive statements to the evaluation of evidence through likelihood judgments. The study included 261 authors from the American Journal of Epidemiology, who were randomly assigned to one of 16 conditions featuring different p-values and treatment magnitudes. Participants were asked to assess whether a randomly drawn individual from the same population would be more likely, less likely, equally likely, or unable to determine the outcome when given drug A compared to drug B. Additionally, participants were asked to provide advice for someone acting as a patient from the same population. The hypothesis tested was whether varying the level of the p-value would alter the correct response option; it was expected that drug A would consistently be described as having higher efficacy than drug B, regardless of the p-value. The authors concluded that their findings demonstrate that researchers across various fields are prone to making erroneous statements and judgments when presented with evidence that does not achieve statistical significance.

McShane et al. published results from a similar study in the Journal of the American Statistical Association [39]. 117 authors responded to the survey that asked the same question as described above, to draw conclusions about the sample, while manipulating a redundant p-value. The results were similar with the ones described above, with the difference that the latter study population (likely to be better educated in statistics) performed better than the previously investigated applied researchers. There was also an investigation of likelihood judgment in the study population being authors in the Journal of the American Statistical Association. The results were consistent with the first study, and it was concluded that the dichotomization of evidence intrinsic to the NHST paradigm leads even expert statisticians to think dichotomously.

These questions in the publications by McShane et al. are not solely concerned with descriptive differences in means between two groups; rather, they can be interpreted as attempts to estimate causal effects. If so, design-based inference to the sample should be conducted. We will further discuss these results under the heading Ambiguities and controversies, below.

**Inferring clinical significance from results from NHST**

Several studies have explored whether *clinical significance* can be inferred from *statistically significant findings*. Clinical significance refers to the practical importance or real-world relevance of a treatment effect. Determining clinical significance involves not only statistical results but also professional clinical judgment about whether the effect is meaningful for patient care. Thus, clinical significance cannot be inferred from statistically significance.

Such studies employed questionnaires with questions specifically designed to identify this fallacy, assessing participants agreement that a p<0.05 is one indication that the results are important [5-7]. All authors of these studies concluded respondents lack of understanding of statistical concepts.

In a 1999 study by Ferrill et al.—described previously—707 pharmacists and pharmacy students in the US responded to two questions regarding the inference of results from NHST [14]. Approximately 32.5% of respondents mistakenly believed that demonstrating statistical significance guarantees the clinical significance of a study's findings. This study also included additional questions on interpreting NHST results and confidence intervals (as discussed both above and under headings below).

Badenes-Ribera et al. conducted a study that examined the knowledge and use of effect size statistics, confidence intervals, and meta-analysis among 472 academic psychologists in Spain [40]. One question specifically asked respondents whether obtaining a statistically significant result implies that the detected effect is important. The results were analyzed based on whether respondents could name an effect size. Among those who could, 54.5% incorrectly endorsed the statement as true, while only 30.3% of those who could not name an effect size did so. In comparison, a similar study by the same research group, involving 159 Italian academic psychologists, reported lower rates of misunderstanding, with 10.2% and 8.5%, respectively [25].

**Questions about inferring confidence intervals**

The interpretation of confidence intervals (CIs) is less frequently examined than the analysis of results derived from NHST, such as p-values and “statistically significant” findings. However, a variety of approaches to investigating the understanding of confidence intervals have been used in the literature. The included studies that have investigated inferences of confidence intervals are summarized below according to the approaches used, by chronological order of appearance in the literature. Additionally, there are studies that investigate the relationship between NHST results and confidence intervals and how such results compare – these studies are presented under a separate heading below.

In the study by Ferrill et al., 707 pharmacists and pharmacy students in the US responded to a question about the characteristics of confidence intervals [14]. The respondents were given the following statements: a) confidence intervals provide an estimate of the range where the true mean value in the affected population is likely to reside, b) are abbreviated as CI or C.I., c) the most commonly used confidence interval is 95 percent, d) all of the above or e) I don’t know. Authors write that they consider d as the correct answer which was identified by 22.9% of the responders. As this standpoint involves an interpretation that is disputed (response alternative a), it is also discussed under the heading of Ambiguities and controverses below.

Cummings et al, conducted a study to investigate researchers' understanding of confidence intervals, standard error bars, and the concept of replication [41]. The authors reached out to authors having published in psychology, behavioral science, and medical journals, resulting in a sample of approximately 267 respondents. Participants were shown images of confidence intervals or standard error bars and were asked to create a plausible set of 10 replications based on the displayed information. The results revealed a consistent pattern aligned with what the authors termed the "confidence level misconception." Specifically, many respondents believed that approximately 95% of future means would fall within the original confidence interval. In fact, the authors provided simulations indicating that an observed 95% confidence intervals will, on average, capture about 83.4% of future replicated means. The conclusion was that the confidence level misconception was held by a majority of participants.

In 2005, the same research group, further investigated researchers' inferences regarding confidence intervals and error bars [42]. The study involved contacting authors of publications in various journals, resulting in a sample of 473 respondents. One key question required participants to graphically adjust confidence intervals for two independent groups until the means appeared to be just statistically different (p < 0.05). The findings revealed significant variability in responses, with only a small proportion of participants provided accurate adjustments. Many respondents tended to set the means too far apart, demonstrating a lack of understanding that achieving a p-value of less than 0.05 necessitates some overlap between the confidence intervals. The authors concluded that a considerable number of researchers hold fundamental and severe misconceptions about the appropriate use of confidence intervals to support inferences drawn from data.

In the investigation by Windish et al., the knowledge of various biostatistical concepts was assessed among 277 medical residents in the US [16]. One of the 20 questions asked respondents to use a 95% CI to determine the direction of an association and its statistical significance. This question required respondents to apply the confidence interval for hypothesis testing rather than interpreting it in terms of parameter estimation. Only 11.9% of the participants selected the correct response of the four possible response alternatives.

The same question, albeit with a slightly modified vignette, was used in both a pilot study and a larger study, where the latter targeted 123 plastic surgery residents enrolled in accredited training programs in the United States [43, 44]. The percentage of respondents providing the correct answer to the CI question was 45.5% in the pilot study and 26.0% in the larger study, indicating a persistent lack of understanding regarding how confidence intervals can be used in hypothesis testing.

In 2014 Hoekstra et al. published an article titled "Robust Misinterpretation of Confidence Intervals" [45]. The authors reported a study which examined the understanding of confidence intervals among bachelor and master’s students (n=442), as well as researchers (n=120) at Dutch universities. Participants completed a written survey that described a fictitious scenario in which a professor conducts an experiment and reports a 95% confidence interval (CI) for the mean ranging from 0.1 to 0.4. The participants were presented with six statements representing common misconceptions about confidence intervals and asked to indicate whether each statement was true or false. All statements were false, designed to mimic questions about null hypothesis significance testing previously used by Oakes and others. The questions used by Hoekstra et al. are presented in Table 2. The results showed that the mean number of incorrectly endorsed statements among the groups was as follows: first-year students (3.51), master’s students (3.24), and researchers (3.45). Alarmingly, only about 2% of participants across the entire group correctly identified all statements as false. The authors also analysed the relationship between self-assessed experience level and the number of endorsed misconceptions, finding a weak and non-significant correlation (r=0.04; 99% CI = [−0.20; 0.27]), which was contrary to expectations. The authors concluded that many researchers in psychology lack a correct understanding of confidence intervals, which is particularly concerning given that these intervals are essential tools for drawing conclusions from data.

Garcia-Perez et al. replicated Hoekstra et al.'s investigation with a Spanish sample comprising 313 first-year psychology students and 158 master’s students [46]. The authors critiqued Hoekstra et al.'s methods and questions, particularly noting that all statements in the original study were false. They introduced two additional statements that they claimed were correct (these statements will be further discussed under the heading Ambiguities and controversies, below). García-Pérez et al. focused their analyses on the differences between first year and master’s students, as well as the variations when compared to Hoekstra et al.’s results. Despite this emphasis, they found that the same statements used in Hoekstra et al.'s study were endorsed by approximately 50% to 80% of first-year students. Nevertheless, the authors concluded that their findings indicated that the misinterpretation of confidence intervals was not as prevalent and widespread as suggested by Hoekstra et al.

Two studies by Lyu et al. investigated inferences of CIs using the instrument developed by Hoekstra et al [27, 28]. The first study involved 362 university students and researchers in China, revealing that approximately 20% to 50% of respondents endorsed misconceptions about CIs, as assessed from graphical data. Notably, about 90% of participants endorsed at least one false statement. In the second study, 1,479 Chinese participants from various scientific fields and with different academic backgrounds were randomly assigned to one of two survey versions: one presented a CI vignette with a significant scenario (where CIs did not include zero) and the other featured a non-significant scenario (where CIs included zero). Participants were then asked to evaluate six statements about the meaning of the CI, all of which were false. They also rated their confidence in their judgments on a five-point Likert scale. Results from the significant CI scenario showed that endorsement of false statements ranged from 30% to 70% across different scientific fields, with a total of 94% of participants endorsing at least one false statement. For those presented the non-significant CI scenario, false statement endorsements ranged from 33% to 68%, with 91% of participants endorsing at least one false statement. The authors concluded that misinterpretations of CIs are prevalent within the Chinese scientific community, affecting participants regardless of the scenario presented.

In 2018, Kalinowski et al. published findings from a study involving 101 undergraduate and graduate students that explored their intuitions regarding the probability distribution underlying a confidence interval, which the authors referred to as the "subjective likelihood distribution" [47]. Participants engaged in three tasks: they were asked to rate the relative likelihood of several points, both inside and outside a visualized confidence interval, representing the population parameter. They also selected the shape of a distribution that best represented their subjective likelihood distribution and indicated the width of confidence intervals at different confidence levels. The authors claimed that the results revealed students harbored competing intuitions about confidence intervals, leading to the identification of several new misconceptions related to CIs. Overall, the study highlighted the complexity of students' understanding of confidence intervals and underscored the need for better education on the topic.

In 2021, Hayat et al. published the results of a study investigating statistics knowledge among nursing faculty in the United States [48]. A total of 164 participants responded to the questionnaire, representing a response rate of 7.7%. One of the questions asked participants to interpret a 95% confidence interval (CI). Among four response options, 42.7% of participants correctly identified the accurate interpretation: "If you draw repeated random samples and calculate a confidence interval for each, you can expect 95% of those intervals to contain the true mean.".

*Approaches to compare inferences of NHST and CIs*

Another approach in studies investigating inferences is to examine interpretations and misunderstandings when results described using NHST are compared with those presented through confidence intervals.

Fidler et al. conducted a study involving two surveys (also detailed in a thesis by [49]) with undergraduate students in environmental science and ecology at an Australian university [50]. The study aimed to determine whether CIs, compared to results presented as NHST, reduce the tendency to interpret statistical non-significance as indicating "no effect" in experimental scenarios with low statistical power. Despite having received multiple warnings about this misconception along with formal instruction on statistical power analysis during their coursework, the results showed that 39% of participants demonstrated the misconception in the NHST scenarios, while only 13% did so in the CI scenarios. The authors concluded that CIs possess a cognitive advantage over NHST in reducing the common misconception that statistical non-significance implies "no effect."

In 2010 Coulson et al. addressed a similar question in two studies reported in the same publication [51]. In the first study, they contacted authors of articles published in psychology, behavioral neuroscience, and medical journals, inviting them to interpret two fictitious studies that yielded similar results - one statistically significant and the other non-significant. The results from both studies were not conflicting. Respondents (n=330) were presented with various formats of NHST results, either in text or figure form, alongside CI results based on the same data. They were then asked to rate their agreement with the conclusions presented in the vignettes. The authors reported significant variability in responses, with interpretations generally being poor regardless of whether the results were presented as CIs or NHST. In a second study, presented in the same publication, involving academic staff from psychology departments (n=50), they confirmed that CIs tended to elicit better interpretations when NHST was not invoked.

In 2012, Hoekstra et al. published a study involving 66 PhD students from various universities in the Netherlands, who were asked to interpret statistical outcomes presented as confidence intervals or conventional statistics using t-tests and associated p-values [52]. Participants were given a vignette describing a double-blinded placebo-controlled experiment, where means were presented in both CI and NHST formats. The scenarios varied, with half of the cases indicating that means were greater in the CI scenario, while the other half indicated greater means in the NHST scenario. Four different p-value levels were employed: 0.02, 0.04, 0.06, and 0.13, with the order of presentation randomized for each participant. Respondents were asked about their confidence in the alternative hypothesis and the likelihood of replicating the study's outcomes. Specifically, they were asked to estimate the probability of an effect in the expected direction in the population and the probability of finding a significant effect in a repeat study. Responses were provided in percentage form. Participants also had to explain what these results indicated about the population and how they reached their conclusions. These textual answers were analyzed to identify inferential mistakes, such as incorrectly accepting the null hypothesis or the alternative hypothesis as true. The findings revealed that the mean confidence in the alternative hypothesis and estimates of replicability were, on average, lower for scenarios presented with CIs than for those using NHST. Contrary to the authors' expectations, a cliff effect - where respondents displayed a stark shift in confidence based on specific p-value thresholds - was observed more frequently in CI scenarios. However, there were also more inferential mistakes (i.e., incorrectly accepting the null hypothesis or the alternative hypothesis) in the NHST scenarios compared to the CI scenarios. The authors concluded that presenting results as CIs led to fewer misinterpretations of statistics, such as accepting the null hypothesis, and that references to effect size were more common in CI presentations.

**Ambiguities and controversies**

**Ambiguities about inferences of results from null hypothesis significance testing**

The literature on empirical investigations of inferential reasoning often aims to highlight common errors and misconceptions in interpreting statistical inferences. However, it can also contribute to ambiguities, uncertainties, and even controversies. One reason for this is that the underlying concepts and terminology are frequently vague and poorly defined. Null Hypothesis Significance Testing (NHST) is a procedure that combines p-values with significance levels (α) used in hypothesis testing. This merging of two distinct approaches—significance testing, which gauges the strength of evidence against a null hypothesis in the sample, and hypothesis testing in a population at a given significance level, to produce a binary decision aiming at controlling long term error rates-can lead to confusion when interpreting statistical outcomes and their implications. Several of the included studies claim to investigate misunderstandings about p-values, while their actual focus is wider, examining inferences of p-values within the context of results from NHST [12, 24, 37, 40, 53]. For example, in the article published by Tam et al. participants are presented with a vignette of a tested treatment where the authors claim that a test shows that a new drug was superior to the old drug at lowering blood pressure (p=0.05) [37]. Respondents were then, asked to interpret what p=0.05 means. While some may understand the vignette as presenting a statistically significant result, others may think that since no alfa-level is reported one cannot make such claim.

There are also examples of questions which are difficult to respond to. For example, Mittag et al. asked respondents to rate on a five graded Likert scale if they agree or not that “Finding that p < .05 is one indication that the results are important.” [5]. Even if you know that a statistically significant finding does not imply importance, many probably agree that p<.05 could certainly be *one* indication out of many to determine clinical or practical importance. So, facing a five-item rating scale, what is a rational answer?

A more serious concern is that authors themselves sometimes do not seem to fully understand the underlying theories and their implications, which results in questions and answers that promote misunderstandings. In an article by Alothman et al., respondents are asked that “If you reject the null hypothesis at 10% then a) it will be rejected at 5%, b) it could be rejected or accepted at 5% c) it can’t be rejected at 5% [54]. These response alternatives make no sense within the practice of hypothesis testing, as the significance level should be set by the analysist ahead of testing. The same study also asked participants what the p-value means and provided the following response alternatives a) probability to reject the null hypothesis when it’s true. b) chance of accept null hypothesis when it’s true. c) probability of reject alternative when it’s true. None of the alternatives are right, yet the authors incorrectly present a) as the correct alternative.

In two publications by Badenes-Ribera et al., participants were presented with ten statements, eight of which represented common fallacies about conclusions drawn from Null Hypothesis Significance Testing (NHST) [24, 25]. The vignette provided was as follows: “Suppose that a research article indicates a value of p = 0.001 in the results section (α = 0.05). Select the true statements. The authors identified the following statements as correct: “The probability of the result of the statistical test is known, assuming that the null hypothesis is true” and “Given that p = 0.001, the result obtained makes it possible to conclude that the differences are not due to chance.” However, both statements are incorrect. Neither the p-value nor the α-level can be interpreted as the probability of the test result given that the null hypothesis is true. Additionally, p-values and statistically significant findings do not provide legitimate grounds to conclude the existence of true differences between groups. These interpretations reflect fundamental misunderstandings of NHST principles and highlight common errors in reasoning about statistical significance.

In the studies by McShane et al. participants were asked a question regarding differences in means between two groups in a sample [38, 39]. As these difference stem from an intervention and an implicit randomized experiment (drug A vs drug B), the question, implicitly, refers to causal effects. With causal effects, inference needs to be conducted also to the sample. This then means that the p-values are not redundant as was the maintained assumption under model-based inference in [38, 39]. Thus, researchers responding that the difference were, greater with lower p-values could very well have been correct as they are aware of the design-based inference for causal effects. The authors also asked participants whether they assumed any specific statistical model when considering the question about differences in the sample. Most respondents indicated that they did not use any specific model, and only a few mentioned a permutation approach based on the randomization assumption.

**Ambiguities about inferences of confidence intervals**

There are also questions and statements about confidence intervals that increase uncertainty, ambiguities and even promote incorrect inferences.

In an from 1999 Ferrill et al. promote the following statement about confidence intervals as one being correct: “Confidence intervals provide an estimate of the range where the true mean value in the affected population is likely to reside” [14]. While this is a vague statement, perhaps supported by intuitive heuristics, it is not supported by the Neyman-Pearson model-based theory, as it has been recognized as the likelihood fallacy.

In an article by Bezzina et al., authors present the following statement about confidence intervals as true: “a confidence interval is a statement about the unknown population parameter” [23]. Arguably, that statement is not correct. A confidence interval is defined by how a valid and unbiased analysis will perform under unlimited repetitions of the study. It is not a statement about what an observed confidence interval says about the unknown population parameter, despite many believing so.

In an article by Msaouel et al, the authors claim that the most correct interpretation of a relative risk with a 95% confidence interval with a lower and upper limit of 1.10 to 2.45 is that “there is a 95% likelihood that the true relative risk falls between 1.10 and 2.45” [32]. Whether this is the most correct of the four alternatives may be disputed. However, it is incorrect and has been identified as a fallacy.

Another example is the article published by Garcia-Perez et al, a study described above [46]. The study used the same questionnaire as Hoekstra et al [45]. The vignette used was a statement from a researcher who, after conducting an experiment analyzing the data, reports that the 95% CI for the mean ranges from 0.1 to 0.4. The original questionnaire includes six false statements (see Table 2) and Garcia-Perez et al, therefore, added two statements which they claimed are correct interpretations of a confidence interval. These were*: “The claim ’The true mean lies between 0.1 and 0.4‘ is true with probability 0.95”* and *“The data are compatible with the notion that the true mean lies between 0.1 and 0.4”.* These added statements have been identified as fallacies [55, 56].

We have not reported results from one study [53]. In this study, the authors surveyed knowledge about p-values in 1,192 Peruvian students using 11 true-or-false questions. Several of the statements were unclear, and the authors did not indicate what they considered being correct answers, reporting only the proportion of students who answered each question correctly. This makes it impossible for readers to interpret the results meaningfully.

# References

1. Rosenthal R, Gaito J. The Interpretation of Levels of Significance by Psychological Researchers. The Journal of Psychology. 1963;55(1):33-8.

2. Beauchamp KL, May RB. Replication report: Interpretation of levels of significance by psychological researchers. Psychological Reports. 1964;14(1):272-.

3. Nelson N, Rosenthal R, Rosnow RL. Interpretation of significance levels and effect sizes by psychological researchers. American Psychologist. 1986;41(11):1299-301.

4. Poitevineau J, Lecoutre B. Interpretation of significance levels by psychological researchers: The .05 cliff effect may be overstated. Psychonomic Bulletin & Review. 2001;8(4):847-50.

5. Mittag KC, Thompson B. Research news and Comment: A National Survey of AERA Members’ Perceptions of Statistical Significance Tests and Other Statistical Issues. Educational Researcher. 2000;29(4):14-20.

6.Gordon H. American Vocational Education Research Association Members' Perceptions of Statistical Significance Tests and Other Statistical Controversies. Journal of Vocational Education Research. 2001;26(2):244-71.

7.Monterde-i-Bort H, Frías-Navarro D, Pascual-Llobell J. Uses and abuses of statistical significance tests and other statistical resources: a comparative study. European Journal of Psychology o f Education. 2010;25:429-47.

8. Weiss ST, Samet JM. An assessment of physician knowledge of epidemiology and biostatistics. J Med Educ. 1980;55(8):692-7.

9. Oakes M. Statistical Inference: A Commentary for the Social and Behavioural Sciences. Chichester, UK: J. Wiley & Sons, Inc; 1986.

10. Wulff HR, Andersen B, Brandenhoff P, Guttler F. What do doctors know about statistics? Stat Med. 1987;6(1):3-10.

11. Scheutz F, Andersen B, Wulff HR. What do dentists know about statistics? Scand J Dent Res. 1988;96(4):281-7.

12. Freeman PR. The role of p-values in analysing trial results. Stat Med. 1993;12(15-16):1443-52; discussion 53-8.

13. Falk R, Greenbaum CW. Significance Tests Die Hard:The Amazing Persistence of a Probabilistic Misconception. Theory & Psychology. 1995;5(1):75-98.

14. Ferrill M, Norton L, Blalock S. Determining the Statistical Knowledge of Pharmacy Practitioners: A Survey and Review of the Literature1. American Journal of Pharmaceutical Education. 1999;63.

15. Haller H, Krauss S. Misinterpretations of Significance: A Problem Students Share with Their Teachers? Methods of Psychological Research Online. 2002;7:1-20.

16. Windish DM, Huot SJ, Green ML. Medicine residents' understanding of the biostatistics and results in the medical literature. JAMA. 2007;298(9):1010-22.

17. Polychronopoulou A, Eliades T, Taoufik K, Papadopoulos MA, Athanasiou AE. Knowledge of European orthodontic postgraduate students on biostatistics. Eur J Orthod. 2011;33(4):434-40.

18. Bookstaver PB, Miller AD, Felder TM, Tice DL, Norris LB, Sutton SS. Assessing pharmacy residents' knowledge of biostatistics and research study design. Ann Pharmacother. 2012;46(7-8):991-9.

19. Best AM, Laskin DM. Oral and maxillofacial surgery residents have poor understanding of biostatistics. J Oral Maxillofac Surg. 2013;71(1):227-34.

20. Kalinowski P, Fidler F, Cumming G. Overcoming the inverse probability fallacy: A comparison of two teaching interventions. Methodology: European Journal of Research Methods for the Behavioral and Social Sciences. 2008;4(4):152-8.

21. Sotos Castro AE, Vanhoof S, Van den Noortgate W, Onghena P. How Confident are Students in their Misconceptions about Hypothesis Tests? Journal of Statistics Education. 2009;17(2):null-null.

22. Westover MB, Westover KD, Bianchi MT. Significance testing as perverse probabilistic reasoning. BMC Med. 2011;9:20.

23. Bezzina F, Saunders M. The pervasiveness and implications of statistical misconceptions among academics with a special interest in business research methods. Electronic Journal of Business Research Methods. 2014;12:103-14.

24. Badenes-Ribera L, Frias-Navarro D, Monterde-i-Bort H, Pascual-Soler M. Interpretation of the p value: A national survey study in academic psychologists from Spain. Psicothema. 2015;27(3):290-5.

25. Badenes-Ribera L, Frias-Navarro D, Iotti B, Bonilla-Campos A, Longobardi C. Misconceptions of the p-value among Chilean and Italian Academic Psychologists. Front Psychol. 2016;7:1247.

26. Ferreira C, Abreu T, Basto M. p-VALUE MISCONCEPTIONS IN HEALTH PROFESSIONALS AND PATIENTS. Advances and Applications in Statistics. 2020;63:75-83.

27. Lyu Z, Peng K, Hu CP. P-Value, Confidence Intervals, and Statistical Inference: A New Dataset of Misinterpretation. Front Psychol. 2018;9:868.

28. Lyu X, Xu Y, Zuo X-N, Chuan-Peng H. Beyond psychology: prevalence of p value and confidence interval misinterpretation across different fields. Journal of Pacific Rim Psychology. 2020;14.

29. Rajčáni J, Vargová L, Adamkovič M, Kačmár P. Statistical Misconceptions, Awareness, and Attitudes towards Open Science Practices in Slovak Psychology Researchers. Studia Psychologica. 2023;65:56-70.

30. Lytsy P, Hartman M, Pingel R. Misinterpretations of P-values and statistical tests persist among researchers and professionals working with statistics and epidemiology. Upsala Journal of Medical Sciences. 2022;127(1).

31. Vallecillos A. Understanding of the Logic of Hypothesis Testing Amongst University Students. Journal für Mathematik-Didaktik. 2000;21(2):101-23.

32. Msaouel P, Kappos T, Tasoulis A, Apostolopoulos AP, Lekkas I, Tripodaki ES, et al. Assessment of cognitive biases and biostatistics knowledge of medical residents: a multicenter, cross-sectional questionnaire study. Med Educ Online. 2014;19:23646.

33. Lecoutre M-P, Poitevineau J, Lecoutre B. Even statisticians are not immune to misinterpretations of Null Hypothesis Significance Tests. International Journal of Psychology. 2003:37-45.

34. van den Akker OR, Wicherts JM, Alvarez LD, Bakker M, van Assen M. How do psychology researchers interpret the results of multiple replication studies? Psychon Bull Rev. 2023;30(4):1609-20.

35. Kühberger A, Fritz A, Lermer E, Scherndl T. The significance fallacy in inferential statistics. BMC Research Notes. 2015;8(1):84.

36. Lai J, Fidler F, Cumming G. Subjective p intervals: Researchers underestimate the variability of p values over replication. Methodology: European Journal of Research Methods for the Behavioral and Social Sciences. 2012;8(2):51-62.

37. Tam CWM, Khan AH, Knight A, Rhee J, Price K, McLean K. How doctors conceptualise P values: A mixed methods study. Aust J Gen Pract. 2018;47(10):705-10.

38. McShane BB, Gal D. Blinding Us to the Obvious? The Effect of Statistical Training on the Evaluation of Evidence. Management Science. 2016;62(6):1707-18.

39. McShane B, Gal D. Statistical Significance and the Dichotomization of Evidence. Journal of the American Statistical Association. 2017;112:885-95.

40. Badenes-Ribera L, Frias-Navarro D, Pascual-Soler M, Monterde IBH. Knowledge level of effect size statistics, confidence intervals and meta-analysis in Spanish academic psychologists. Psicothema. 2016;28(4):448-56.

41. Cumming G, Williams J, Fidler F. Replication and Researchers' Understanding of Confidence Intervals and Standard Error Bars. Understanding Statistics. 2004;3:299-311.

42. Belia S, Fidler F, Williams J, Cumming G. Researchers misunderstand confidence intervals and standard error bars. Psychol Methods. 2005;10(4):389-96.

43. Susarla SM, Redett RJ. Plastic surgery residents' attitudes and understanding of biostatistics: a pilot study. J Surg Educ. 2014;71(4):574-9.

44. Susarla SM, Lifchez SD, Losee J, Hultman CS, Redett RJ. Plastic Surgery Residents' Understanding and Attitudes Toward Biostatistics: A National Survey. Ann Plast Surg. 2016;77(2):231-6.

45. Hoekstra R, Morey RD, Rouder JN, Wagenmakers EJ. Robust misinterpretation of confidence intervals. Psychon Bull Rev. 2014;21(5):1157-64.

46. Garcia-Perez MA, Alcala-Quintana R. The Interpretation of Scholars' Interpretations of Confidence Intervals: Criticism, Replication, and Extension of Hoekstra et al. (2014). Front Psychol. 2016;7:1042.

47. Kalinowski P, Lai J, Cumming G. A Cross-Sectional Analysis of Students' Intuitions When Interpreting CIs. Front Psychol. 2018;9:112.

48. Hayat MJ, Kim M, Schwartz TA, Jiroutek MR. A study of statistics knowledge among nurse faculty in schools with research doctorate programs. Nurs Outlook. 2021;69(2):228-33.

49. Fidler F. From statistical significance to effect estimation : statistical reform in psychology, medicine and ecology 2005.

50. Fidler F, Loftus GR. Why figures with error bars should replace p values: Some conceptual arguments and empirical demonstrations. Zeitschrift für Psychologie/Journal of Psychology. 2009;217(1):27-37.

51. Coulson M, Healey M, Fidler F, Cumming G. Confidence intervals permit, but don't guarantee, better inference than statistical significance testing. Frontiers in Psychology. 2010;1.

52. Hoekstra R, Johnson A, Kiers HAL. Confidence intervals make a difference: Effects of showing confidence intervals on inferential reasoning. Educational and Psychological Measurement. 2012;72(6):1039-52.

53. Vera-Ponce VJ, Torres-Malca JR, Zuzunaga-Montoya FE, Cruz-Vargas JADL, Cruz-Ausejo L. Factors associated with knowledge of p-value in a sample of medical students. Medicina clínica y social. 2022;6:103-8.

54. Alothman H, Masuadi, E. , Alothman, T. , Almehmadi, S. , Alenazi, M. , Alothman, Ferwana, M. What Physicians Know about Statistics in Saudi Arabia. Open Journal of Social Sciences. 2020;8:282-94.

55. Morey RD, Hoekstra R, Rouder JN, Lee MD, Wagenmakers EJ. The fallacy of placing confidence in confidence intervals. Psychon Bull Rev. 2016;23(1):103-23.

56. Greenland S, Senn SJ, Rothman KJ, Carlin JB, Poole C, Goodman SN, et al. Statistical tests, P values, confidence intervals, and power: a guide to misinterpretations. European Journal of Epidemiology. 2016;31(4):337-50.
