# Supplementary material for: Inferences of p-values, statistically significant results, and confidence intervals – A systematic review of empirical studies
Source: PLoS One. 2026 Nov 11;21(11):e0362071. doi: 10.1371/journal.pone.0362071 (PMC13754157; doi:10.1371/journal.pone.0362071)
Supplement: Supplementary file 5 [file pone.0362071.s006.docx]

**Response to Reviewers**

Dear Editor,

We thank you and both reviewers for your careful reading of our manuscript and for your constructive comments. We have revised the manuscript accordingly.

The most substantial change is a largely rewritten Results section, made in response to Reviewer 1's suggestion for a more concise and synthetic presentation. The Results section is now considerably shorter. This was not without challenges given the substantial heterogeneity across studies, and we therefore also suggest that the more detailed Results section is made available as a supplement.

We have not followed all suggestions. Reviewer 1's suggestion of a temporal analysis of the prevalence of misconceptions has not been implemented, as we do not believe any robust trend can be identified given the heterogeneity. We have, however, identified an important related question, which is now addressed in the Discussion. Reviewer 2's suggestion to make concrete solutions more prominent has likewise not been followed, as we believe this falls outside the scope of the present descriptive study; this is elaborated in the point-by-point response below.

In accordance with PLOS ONE's requirements for supporting information, we have added captions with titles and brief descriptions for all three supplementary files (S1 Text, S2 Table, and S3 Text) at the end of the main manuscript file. We have furthermore made some minor changes to wording and phrasing, available in track changes.

We have aimed to address all other comments and suggestions, and a point-by-point response is provided below. Should the editor or reviewers feel that further revisions are warranted, we remain happy to consider additional changes.

Yours sincerely,

Per Lytsy, corresponding author

**Response to Reviewer 1**

We thank Reviewer 1 for the positive assessment and for the detailed and constructive suggestions.

**Comment 1:** The Results section (lines 198–861) is too lengthy and reduces interpretability. A more concise and synthetic presentation is recommended, with detailed results moved to Supplementary Material.

**Response:** We understand and agree with this comment. We have restructured the Results section to provide a more concise and synthetic presentation of the main findings. This presented some challenges, given the substantial heterogeneity across included studies — both in study populations and in how similar misconceptions were operationalized. Some authors used single items to assess understanding, while others employed multi-item questionnaires, making direct synthesis difficult. The original structure also aimed to present studies in chronological order of appearance in the literature, which was harder to maintain once results were condensed thematically. We have therefore retained the detailed Results section in its original form as Supplementary Material.

Please see the revised manuscript (tracked changes version) for full details.

**Comment 2:** Typo – "for studies empirical studies" in the abstract (line 20).

**Response:** Corrected. Thank you. The word “studies” is omitted.

**Comment 3:** Typo – "Frace" instead of "France" in Table 1.

**Response**: Corrected. Thank you!

**Comment 4:** Inconsistent use of "US" and "USA" in Table 1.

**Response:** We have standardized the abbreviation throughout Table 1 and also made a few changes in the detailed results section (available as Supplementary Material), consistently using 'US' throughout.

**Comment 5**: Replace "people" with a more precise description of target populations (line 864).

**Response:** We have replaced the word, and the sentence now reads: "We conducted a systematic review to identify studies examining how researchers, educators, students, and professionals understand, infer, and interpret results from null hypothesis significance testing (NHST)—such as p-values and statistically significant findings—as well as from estimation approaches, such as confidence intervals”

**Comment 6:** Rephrase the difficult sentence about Neyman and confidence intervals (lines 961–963).

**Response:** We have rephrased the sentence to read: “Neyman himself was explicit that the practical use of confidence intervals does not involve inductive reasoning about the parameter, but rather an act of will — a deliberate decision to adjust our behavior in accordance with the confidence limits”

**Comment 7**: Include an analysis of whether the prevalence of misconceptions has increased or decreased over the decades, particularly following the emergence of the replication crisis debate (around 2015).

**Response**: We thank the reviewer for this interesting suggestion, and we have thoroughly considered it. We do, however, not believe a temporal analysis can be conducted in a meaningful way. The included studies differ substantially in population, setting, question format, and criteria for correct answers, making direct comparisons across time methodologically untenable. Any apparent trend would more likely reflect methodological heterogeneity than genuine change in prevalence.

We do, however, agree this deserves mention. We have added a brief discussion noting that the available evidence does not permit a robust test of whether the replication crisis debate has reduced misconception prevalence, and that the overall pattern across studies does not suggest any obvious decline — which is itself a noteworthy observation.

The following text has been added at the end of Discussion/Limitations section:

“Finally, the heterogeneity in populations, methodologies, and instruments precludes meaningful pooling of results and, for the same reason, any formal analysis of whether misconception prevalence has changed over time. To facilitate such comparisons in the future, there is a clear need to develop and validate standardized methods for assessing inferential reasoning. One might expect that the intensified methodological debate following the replication crisis around 2015 would have led to a measurable reduction in misconception prevalence, but nothing in the available evidence suggests any obvious decline.”

**Response to Reviewer 2**

We thank Reviewer 2 for the positive overall assessment and for the recommendation to streamline the presentation of results and include more concrete solutions.

**Comment 1**: The manuscript would better serve readers through a more streamlined presentation with synthesized findings.

**Response:** We agree. As described in our response to Reviewer 1 above, we have substantially rewritten the Results section to provide a more concise and thematically synthesized presentation, with the detailed study-by-study descriptions retained in Supplementary Material. Regarding quantitative summaries, we have added frequencies and percentages where feasible, but note that the substantial heterogeneity across studies limits the extent to which meaningful quantitative synthesis is possible.

**Comment 2:** Concrete solutions to address the identified problems should be more prominent.

**Response:** We thank the reviewer for this suggestion. However, we believe that concrete recommendations or solutions for addressing the identified problems fall outside the scope of the present study, whose aim was descriptive — to systematically identify and assess research on how statistical results are interpreted and inferred. We note that some of the included studies do examine teaching interventions, and these suggest that targeted instruction can reduce specific misconceptions. However, drawing firm conclusions about which interventions are effective, for which populations, and for which types of misconceptions, would require a dedicated systematic review. We would therefore risk doing so superficially without a sufficient basis for such recommendations. We do, however, highlight in the discussion that the findings point to a clear need for improved statistical education and standardized assessment methods. Specifically, the following text is included in the Discussion: "These pervasive misconceptions point to a systemic issue in statistical education and scientific communication. Our findings underscore an urgent need to improve statistical training, promote more nuanced interpretations of statistical results, and encourage reforms in how statistical evidence is presented in scientific research." We also encourage relevant statistical organizations — specifically the American Statistical Association — to take an active role by producing a formal statement on the interpretation and proper use of confidence intervals in research and decision-making.

**Comment 3 (Minor):** Line 147: Consider using "p-value" consistently throughout (lowercase "p") rather than "P-value" for standardization.

Response: Response: We thank the reviewer for this observation. The capitalised form "P-value" on line 147 refers to the specific search term used in the literature search strategy, and has therefore been retained as is. Throughout the remainder of the manuscript, "p-value" (lowercase) is used consistently.

**Comment 4 (Minor): Lines 203–207 – provide frequencies or percentages for each category mentioned.**

Response: We have added frequencies and percentages for each category. Thank you for this suggestion.

**Comment 5 (Minor):** Line 208 – provide measures of central tendency and variability alongside the range.

**Response:** We thank the reviewer for this suggestion. However, given that several studies reported participants across multiple subsamples, and that the distribution of sample sizes is highly skewed with a few very large studies, we feel that adding measures of central tendency would be more misleading than informative. We have therefore retained the range only.

**Comment 6 (Minor):** Line 209 – quantify "typically"

**Response:** We thank the reviewer for this suggestion. We have revised the sentence to read: "The great majority of included studies were cross-sectional surveys using questionnaires for the assessment of inference." A small number of studies had designs that were difficult to classify as strictly cross-sectional, due to features such as multiple sub-studies, pre/post intervention designs, or mixed-methods approaches.

**Comment 7 (Minor): Lines 210–234 and Results generally – supplement qualitative descriptions with quantitative summaries.**

**Response**: We have added quantitative summaries throughout the Results section where feasible, including frequencies and percentages in the introductory description of study characteristics and proportions of respondents endorsing specific misconceptions within each thematic subsection. Thank you for this suggestion

**Comment 8 (Minor): Lines 1031–1035 – repetition in passage, please revise.**

**Response:** We have revised this passage to eliminate the redundancy. Thank you!
