# Supplementary material for: Inferences of p-values, statistically significant results, and confidence intervals – A systematic review of empirical studies
Source: PLoS One. 2026 Nov 11;21(11):e0362071. doi: 10.1371/journal.pone.0362071 (PMC13754157; doi:10.1371/journal.pone.0362071)
Supplement: Supplementary file 6 [file pone.0362071.s007.docx]

Dear Editors,

We thank you and the reviewer for the constructive comments on our manuscript. We have revised the Results section to provide additional quantitative information, clarified the development and registration status of the research plan, and addressed the comment regarding the availability of the complete search strategies.

Below, we provide a point-by-point response to the reviewer’s comments. We have also made minor corrections to the language and presentation of the manuscript.

Yours sincerely,
Per Lytsy
Corresponding author

**Reviewers' comments and responses,**

Review Comments to the Author

**Reviewer #1:** I thank the authors for their careful and constructive revision of the manuscript. The paper has improved substantially compared to the previous version, particularly in the restructuring of the Results section, which is now more readable and thematically organized.
At the same time, some issues remain that, in my opinion, should be addressed before the manuscript can be considered for publication.

**Response**. Thank you and sorry about the time delay. We have tried to address and clarify the questions you raise, please see details below.

**- Minor Issues:**

**1. Level of detail in the Results section**
While the effort to synthesize the Results section is appreciated and represents a clear improvement, the current version at times becomes too condensed, with a loss of informative detail.
In several instances, statements about participants’ misunderstandings remain qualitative and somewhat generic, without providing quantitative support. For example:
“[...] responses were frequently unsatisfactory, with many participants providing erroneous or illogical answers [...]”
“A majority of the participants endorsed several of these incorrect statements as true [...]”
Such formulations reduce interpretability and make it difficult for the reader to assess the magnitude of the reported effects.
In contrast, passages where proportions are reported (e.g., “Only 13.2% of participants correctly identified…”) are much more informative and should be preferred.

**Suggestion:** I recommend that the authors systematically include key quantitative indicators (e.g., percentages of correct/incorrect responses) in the main text when available, while still keeping extended details in the Supplementary Materials. This would improve both clarity and interpretability without substantially increasing length.

**Response:** We thank the reviewer for this helpful suggestion. We carefully reviewed the entire Results section and have added proportions of participants providing correct or incorrect responses for several of the main approaches and instruments where such information was available and informative. We have also replaced or supplemented several general qualitative descriptions with specific numerical results and added a short statement explaining our approach to quantitative reporting.

The included studies are highly heterogeneous in their populations, questions, instruments, and response formats, and their findings are therefore often not directly comparable. We have consequently aimed to provide representative quantitative examples where they aid interpretation, while avoiding potentially misleading comparisons or giving disproportionate weight to individual studies or instruments.

Detailed study-level results remain available in the Supplementary Materials. We believe these revisions improve the clarity and interpretability of the Results while retaining the thematic synthesis.

**- Major Issues:**

**1. Pre-registration and transparency of the review protocol**
The manuscript states that:
“A project plan was developed prior to initiating the study and is available at: https://osf.io/qzxut.”
However, the OSF record appears to have been created in May 27, 2025 and subsequently modified in September 1, 2025, whereas the manuscript reports that the literature search was conducted as early as December 2021 and updated in April 2023 and August 2025.
This raises a serious concern regarding the claim of a priori protocol development. As it stands, the review does not appear to have been pre-registered before data collection began.
This is not a minor issue. Pre-registration is a key component of methodological transparency in systematic reviews, as it allows readers and reviewers to assess the risk of selective reporting and analytic flexibility.

**Required clarification:**
The authors should explicitly clarify the timeline of protocol development.
If the review was not pre-registered prior to data collection, this must be clearly stated in the manuscript.
The current wording should be revised to avoid any implication of prospective registration if this was not the case.
Failure to clarify this point risks misleading the reader about the level of methodological rigor and transparency of the study.

**Response:** We thank the reviewer for raising this important issue and agree that the previous wording was insufficiently clear. The project was initiated in late 2019, and a research plan was developed at the outset, specifying the objectives, research questions, eligibility criteria, and main review procedures. However, the review was not prospectively registered, and the research plan was not made publicly available on OSF until May 2025.

We recognise the value of prospective registration, particularly in reducing the possibility that eligibility criteria or methodological decisions are modified in response to the evidence identified during a review. In our review, the research question and eligibility criteria were deliberately broad: the aim was to identify and map empirical studies investigating inferences from p-values, NHST results, and confidence intervals. We did not exclude otherwise eligible studies based on their findings or methodological limitations. Given this broad and inclusive approach, we consider the risk that the lack of prospective registration influenced study selection to be limited.

We have revised the Methods to clarify the timeline and explicitly state that the review was not prospectively registered. We have also acknowledged the lack of prospective registration as a limitation.

The following text has been added to the Methods section:

“A research plan was developed at the outset of the project but was not prospectively registered. The plan was made publicly available on the Open Science Framework (OSF) in May 2025 ([https://osf.io/qzxut).”](https://osf.io/qzxut).)

The following text has been added to the Limitations section:

“Although a research plan was developed at the outset of the project, it was not prospectively registered, which limits the possibility of independently verifying that the review methods were specified in advance.”

**2. Reproducibility of the search strategy**
The manuscript indicates that detailed search strings are provided in “S1 Text”. However, this material is not accessible (neither in the manuscript nor in the Supplementary files made available for review).
Without the full search strings, the systematic review is not reproducible, and the “systematic” nature of the review cannot be independently evaluated.
This is a fundamental requirement for systematic reviews.

**Required action:**
The authors must provide the complete search strategies for all databases (including exact strings, limits, and filters used).
The referenced “S1 Text” should be included and clearly linked or uploaded as Supplementary Material.

**Response:** We thank the reviewer for pointing this out. The complete search strategies were included in the submitted Supporting Information, including the exact search terms, search fields, combinations, limits, and dates for all four databases. We believe that the confusion may have arisen from inconsistent labelling: the Methods section referred to this material as “S1 Appendix”, whereas the Supporting Information caption referred to it as “S1 Text”.

We have now used “S1 Appendix” consistently throughout the manuscript and Supporting Information and have ensured that the corresponding file is clearly labelled and included with the revised submission.
