# Supplementary material for: Inferences of p-values, statistically significant results, and confidence intervals – A systematic review of empirical studies
Source: PLoS One. 2026 Nov 11;21(11):e0362071. doi: 10.1371/journal.pone.0362071 (PMC13754157; doi:10.1371/journal.pone.0362071)
Supplement: Peer review history

# Peer review: Decision Letter 0

**DOI:** 10.1371/journal.pone.0362071.r001

**Authors:** Elisa Scerrati

11 Feb 2026

Dear Dr. Lytsy,

Please submit your revised manuscript by Mar 28 2026 11:59PM. If you will need more time than this to complete your revisions, please reply to this message or contact the journal office at plosone@plos.org. When you’re ready to submit your revision, log on to https://www.editorialmanager.com/pone/ and select the 'Submissions Needing Revision' folder to locate your manuscript file.

- A letter that responds to each point raised by the academic editor and reviewer(s). You should upload this letter as a separate file labeled 'Response to Reviewers'.
- A marked-up copy of your manuscript that highlights changes made to the original version. You should upload this as a separate file labeled 'Revised Manuscript with Track Changes'.
- An unmarked version of your revised paper without tracked changes. You should upload this as a separate file labeled 'Manuscript'.

If you would like to make changes to your financial disclosure, please include your updated statement in your cover letter. Guidelines for resubmitting your figure files are available below the reviewer comments at the end of this letter.

If applicable, we recommend that you deposit your laboratory protocols in protocols.io to enhance the reproducibility of your results. Protocols.io assigns your protocol its own identifier (DOI) so that it can be cited independently in the future. For instructions see: https://journals.plos.org/plosone/s/submission-guidelines#loc-laboratory-protocols. Additionally, PLOS ONE offers an option for publishing peer-reviewed Lab Protocol articles, which describe protocols hosted on protocols.io. Read more information on sharing protocols at https://plos.org/protocols?utm_medium=editorial-email&utm_source=authorletters&utm_campaign=protocols.

We look forward to receiving your revised manuscript.

Kind regards,

Elisa Scerrati

Academic Editor

PLOS One

Journal Requirements:

When submitting your revision, we need you to address these additional requirements.

1. Please ensure that your manuscript meets PLOS ONE's style requirements, including those for file naming. The PLOS ONE style templates can be found at

https://journals.plos.org/plosone/s/file?id=wjVg/PLOSOne_formatting_sample_main_body.pdf and

https://journals.plos.org/plosone/s/file?id=ba62/PLOSOne_formatting_sample_title_authors_affiliations.pdf

2. Please include captions for your Supporting Information files at the end of your manuscript, and update any in-text citations to match accordingly. Please see our Supporting Information guidelines for more information: http://journals.plos.org/plosone/s/supporting-information.

3. If the reviewer comments include a recommendation to cite specific previously published works, please review and evaluate these publications to determine whether they are relevant and should be cited. There is no requirement to cite these works unless the editor has indicated otherwise.

[Note: HTML markup is below. Please do not edit.]

Reviewers' comments:

Reviewer's Responses to Questions

**Comments to the Author**

1. Is the manuscript technically sound, and do the data support the conclusions?

Reviewer #1: Yes

Reviewer #2: Yes

**********

2. Has the statistical analysis been performed appropriately and rigorously?

Reviewer #1: N/A

Reviewer #2: N/A

**********

3. Have the authors made all data underlying the findings in their manuscript fully available?

The PLOS Data policy

Reviewer #1: Yes

Reviewer #2: Yes

**********

4. Is the manuscript presented in an intelligible fashion and written in standard English?

Reviewer #1: Yes

Reviewer #2: Yes

**********

Reviewer #1: The paper addresses through a systematic review the spread of misconceptions about p-values, confidence intervals and statistical inference among scholars, university students, academics and professionals. The results highlight current and long-going misconceptions about statistical inference among these populations.

The work is timely and important, given the ongoing debate about the replication crisis involving psychology and other sciences. The manuscript is technically sound and the conclusions are appropriate. Suggestions regarding how to deal with current misconceptions about statistical inference are also pertinent and reasonable.

I identified only one main limitation:

1. I find the "Results" section (lines 198–861) rather lengthy, making it challenging to clearly grasp the core findings and thus reducing the overall interpretability of the results. I would suggest offering a more concise and synthetic presentation of the main outcomes, integrating results from similar empirical studies within the same paragraphs to enhance readability and understanding. A comprehensive and detailed report of all results could be provided as Supplementary Material..

I would also suggest correcting the following typos:

- "for studies empirical studies" in the abstract (line 20);

- "Frace" instead of "France" in Table 1 (p. 13).

In addition I recommend:

- aligning the use of "US" and "USA" in Table 1, by choosing and consistently using a single acronym, for consistency;

- replacing the term “people” with a more precise description of the target populations of the studies included in the systematic review in the following sentence: "We conducted a systematic review to identify studies examining how people understand, infer, and interpret results from null hypothesis significance testing (NHST)" (line 864) in order to provide a more accurate narrative for the reader;

- rephrasing the following sentence, which is currently difficult to understand: "Jerzy Neyman, was clear the practical use of confidence intervals did not involve any mental process of inductive reasoning but an act of will to adjust our behavior in accordance with the values of the confidence limits." (lines 961-963).

Overall, I find this work to be a useful contribution to the current literature on the spread of misconceptions about statistical inference among scholars, professors, students, and professionals.

Finally, I believe that including an analysis of whether the prevalence of these misconceptions has increased or decreased over the decades - particularly following the emergence of the replication crisis debate (around 2015) - could further enhance the value of the manuscript.

I remain available for further review rounds or clarifications, if needed.

Reviewer #2: (I have uploaded my review as an attachment.)

The manuscript presents a comprehensive and well-executed systematic review that

documents the pervasive nature of statistical misunderstandings.

The authors have undertaken substantial work confirming a persistent problem in statistical

literacy. However, the manuscript would better serve readers through a more streamlined

presentation with synthesized findings and concrete solutions to address the identified

problems.

**********

what does this mean?). If published, this will include your full peer review and any attached files.

If you choose “no”, your identity will remain anonymous but your review may still be made public.

**Do you want your identity to be public for this peer review?**  For information about this choice, including consent withdrawal, please see our Privacy Policy

Reviewer #1: **Yes:**  Matteo Manente

Reviewer #2: **Yes:**  Margherita Calderan

**********

[NOTE: If reviewer comments were submitted as an attachment file, they will be attached to this email and accessible via the submission site. Please log into your account, locate the manuscript record, and check for the action link "View Attachments". If this link does not appear, there are no attachment files.]

To ensure your figures meet our technical requirements, please review our figure guidelines: https://journals.plos.org/plosone/s/figures

You may also use PLOS’s free figure tool, NAAS, to help you prepare publication quality figures: https://journals.plos.org/plosone/s/figures#loc-tools-for-figure-preparation.

NAAS will assess whether your figures meet our technical requirements by comparing each figure against our figure specifications.

Attachment Submitted filename: review.pdf

# Author comment: Author response to Decision Letter 1

**DOI:** 10.1371/journal.pone.0362071.r002

30 Mar 2026

Response to Reviewers

Dear Editor,

We thank you and both reviewers for your careful reading of our manuscript and for your constructive comments. We have revised the manuscript accordingly.

The most substantial change is a largely rewritten Results section, made in response to Reviewer 1's suggestion for a more concise and synthetic presentation. The Results section is now considerably shorter. This was not without challenges given the substantial heterogeneity across studies, and we therefore also suggest that the more detailed Results section is made available as a supplement.

We have not followed all suggestions. Reviewer 1's suggestion of a temporal analysis of the prevalence of misconceptions has not been implemented, as we do not believe any robust trend can be identified given the heterogeneity. We have, however, identified an important related question, which is now addressed in the Discussion. Reviewer 2's suggestion to make concrete solutions more prominent has likewise not been followed, as we believe this falls outside the scope of the present descriptive study; this is elaborated in the point-by-point response below.

In accordance with PLOS ONE's requirements for supporting information, we have added captions with titles and brief descriptions for all three supplementary files (S1 Text, S2 Table, and S3 Text) at the end of the main manuscript file. We have furthermore made some minor changes to wording and phrasing, available in track changes.

We have aimed to address all other comments and suggestions, and a point-by-point response is provided below. Should the editor or reviewers feel that further revisions are warranted, we remain happy to consider additional changes.

Yours sincerely,

Per Lytsy, corresponding author

Response to Reviewer 1

We thank Reviewer 1 for the positive assessment and for the detailed and constructive suggestions.

Comment 1: The Results section (lines 198–861) is too lengthy and reduces interpretability. A more concise and synthetic presentation is recommended, with detailed results moved to Supplementary Material.

Response: We understand and agree with this comment. We have restructured the Results section to provide a more concise and synthetic presentation of the main findings. This presented some challenges, given the substantial heterogeneity across included studies — both in study populations and in how similar misconceptions were operationalized. Some authors used single items to assess understanding, while others employed multi-item questionnaires, making direct synthesis difficult. The original structure also aimed to present studies in chronological order of appearance in the literature, which was harder to maintain once results were condensed thematically. We have therefore retained the detailed Results section in its original form as Supplementary Material.

Please see the revised manuscript (tracked changes version) for full details.

Comment 2: Typo – "for studies empirical studies" in the abstract (line 20).

Response: Corrected. Thank you. The word “studies” is omitted.

Comment 3: Typo – "Frace" instead of "France" in Table 1.

Response: Corrected. Thank you!

Comment 4: Inconsistent use of "US" and "USA" in Table 1.

Response: We have standardized the abbreviation throughout Table 1 and also made a few changes in the detailed results section (available as Supplementary Material), consistently using 'US' throughout.

Comment 5: Replace "people" with a more precise description of target populations (line 864).

Response: We have replaced the word, and the sentence now reads: "We conducted a systematic review to identify studies examining how researchers, educators, students, and professionals understand, infer, and interpret results from null hypothesis significance testing (NHST)—such as p-values and statistically significant findings—as well as from estimation approaches, such as confidence intervals”

Comment 6: Rephrase the difficult sentence about Neyman and confidence intervals (lines 961–963).

Response: We have rephrased the sentence to read: “Neyman himself was explicit that the practical use of confidence intervals does not involve inductive reasoning about the parameter, but rather an act of will — a deliberate decision to adjust our behavior in accordance with the confidence limits”

Comment 7: Include an analysis of whether the prevalence of misconceptions has increased or decreased over the decades, particularly following the emergence of the replication crisis debate (around 2015).

Response: We thank the reviewer for this interesting suggestion, and we have thoroughly considered it. We do, however, not believe a temporal analysis can be conducted in a meaningful way. The included studies differ substantially in population, setting, question format, and criteria for correct answers, making direct comparisons across time methodologically untenable. Any apparent trend would more likely reflect methodological heterogeneity than genuine change in prevalence.

We do, however, agree this deserves mention. We have added a brief discussion noting that the available evidence does not permit a robust test of whether the replication crisis debate has reduced misconception prevalence, and that the overall pattern across studies does not suggest any obvious decline — which is itself a noteworthy observation.

The following text has been added at the end of Discussion/Limitations section:

“Finally, the heterogeneity in populations, methodologies, and instruments precludes meaningful pooling of results and, for the same reason, any formal analysis of whether misconception prevalence has changed over time. To facilitate such comparisons in the future, there is a clear need to develop and validate standardized methods for assessing inferential reasoning. One might expect that the intensified methodological debate following the replication crisis around 2015 would have led to a measurable reduction in misconception prevalence, but nothing in the available evidence suggests any obvious decline.”

Response to Reviewer 2

We thank Reviewer 2 for the positive overall assessment and for the recommendation to streamline the presentation of results and include more concrete solutions.

Comment 1: The manuscript would better serve readers through a more streamlined presentation with synthesized findings.

Response: We agree. As described in our response to Reviewer 1 above, we have substantially rewritten the Results section to provide a more concise and thematically synthesized presentation, with the detailed study-by-study descriptions retained in Supplementary Material. Regarding quantitative summaries, we have added frequencies and percentages where feasible, but note that the substantial heterogeneity across studies limits the extent to which meaningful quantitative synthesis is possible.

Comment 2: Concrete solutions to address the identified problems should be more prominent.

Response: We thank the reviewer for this suggestion. However, we believe that concrete recommendations or solutions for addressing the identified problems fall outside the scope of the present study, whose aim was descriptive — to systematically identify and assess research on how statistical results are interpreted and inferred. We note that some of the included studies do examine teaching interventions, and these suggest that targeted instruction can reduce specific misconceptions. However, drawing firm conclusions about which interventions are effective, for which populations, and for which types of misconceptions, would require a dedicated systematic review. We would therefore risk doing so superficially without a sufficient basis for such recommendations. We do, however, highlight in the discussion that the findings point to a clear need for improved statistical education and standardized assessment methods. Specifically, the following text is included in the Discussion: "These pervasive misconceptions point to a systemic issue in statistical education and scientific communication. Our findings underscore an urgent need to improve statistical training, promote more nuanced interpretations of statistical results, and encourage reforms in how statistical evidence is presented in scientific research." We also encourage relevant statistical organizations — specifically the American Statistical Association — to take an active role by producing a formal statement on the interpretation and proper use of confidence intervals in research and decision-making.

Comment 3 (Minor): Line 147: Consider using "p-value" consistently throughout (lowercase "p") rather than "P-value" for standardization.

Response: Response: We thank the reviewer for this observation. The capitalised form "P-value" on line 147 refers to the specific search term used in the literature search strategy, and has therefore been retained as is. Throughout the remainder of the manuscript, "p-value" (lowercase) is used consistently.

Comment 4 (Minor): Lines 203–207 – provide frequencies or percentages for each category mentioned.

Response: We have added frequencies and percentages for each category. Thank you for this suggestion.

Comment 5 (Minor): Line 208 – provide measures of central tendency and variability alongside the range.

Response: We thank the reviewer for this suggestion. However, given that several studies reported participants across multiple subsamples, and that the distribution of sample sizes is highly skewed with a few very large studies, we feel that adding measures of central tendency would be more misleading than informative. We have therefore retained the range only.

Comment 6 (Minor): Line 209 – quantify "typically"

Response: We thank the reviewer for this suggestion. We have revised the sentence to read: "The great majority of included studies were cross-sectional surveys using questionnaires for the assessment of inference." A small number of studies had designs that were difficult to classify as strictly cross-sectional, due to features such as multiple sub-studies, pre/post intervention designs, or mixed-methods approaches.

Comment 7 (Minor): Lines 210–234 and Results generally – supplement qualitative descriptions with quantitative summaries.

Response: We have added quantitative summaries throughout the Results section where feasible, including frequencies and percentages in the introductory description of study characteristics and proportions of respondents endorsing specific misconceptions within each thematic subsection. Thank you for this suggestion

Comment 8 (Minor): Lines 1031–1035 – repetition in passage, please revise.

Response: We have revised this passage to eliminate the redundancy. Thank you!

Attachment Submitted filename: Response to reviewers.docx

# Peer review: Decision Letter 1

**DOI:** 10.1371/journal.pone.0362071.r003

**Authors:** Elisa Scerrati

22 Apr 2026

Dear Dr. Lytsy,

Please submit your revised manuscript by Jun 06 2026 11:59PM. If you will need more time than this to complete your revisions, please reply to this message or contact the journal office at plosone@plos.org. When you're ready to submit your revision, log on to https://www.editorialmanager.com/pone/ and select the 'Submissions Needing Revision' folder to locate your manuscript file.

- A letter that responds to each point raised by the academic editor and reviewer(s). You should upload this letter as a separate file labeled 'Response to Reviewers'.
- A marked-up copy of your manuscript that highlights changes made to the original version. You should upload this as a separate file labeled 'Revised Manuscript with Track Changes'.
- An unmarked version of your revised paper without tracked changes. You should upload this as a separate file labeled 'Manuscript'.

If you would like to make changes to your financial disclosure, please include your updated statement in your cover letter. Guidelines for resubmitting your figure files are available below the reviewer comments at the end of this letter.

If applicable, we recommend that you deposit your laboratory protocols in protocols.io to enhance the reproducibility of your results. Protocols.io assigns your protocol its own identifier (DOI) so that it can be cited independently in the future. For instructions see: https://journals.plos.org/plosone/s/submission-guidelines#loc-laboratory-protocols. Additionally, PLOS ONE offers an option for publishing peer-reviewed Lab Protocol articles, which describe protocols hosted on protocols.io. Read more information on sharing protocols at https://plos.org/protocols?utm_medium=editorial-email&utm_source=authorletters&utm_campaign=protocols.

As the corresponding author, your ORCID iD is verified in the submission system and will appear in the published article. PLOS supports the use of ORCID, and we encourage all coauthors to register for an ORCID iD and use it as well. Please encourage your coauthors to verify their ORCID iD within the submission system before final acceptance, as unverified ORCID iDs will not appear in the published article. *Only*  the individual author can complete the verification step; PLOS staff *cannot*  verify ORCID iDs on behalf of authors.

We look forward to receiving your revised manuscript.

Kind regards,

Elisa Scerrati

Academic Editor

PLOS One

Journal Requirements:

If the reviewer comments include a recommendation to cite specific previously published works, please review and evaluate these publications to determine whether they are relevant and should be cited. There is no requirement to cite these works unless the editor has indicated otherwise.

[Note: HTML markup is below. Please do not edit.]

Reviewers' comments:

Reviewer's Responses to Questions

**Comments to the Author**

Reviewer #1: (No Response)

Reviewer #2: All comments have been addressed

**********

2. Is the manuscript technically sound, and do the data support the conclusions?

Reviewer #1: Partly

Reviewer #2: Yes

**********

3. Has the statistical analysis been performed appropriately and rigorously?

Reviewer #1: N/A

Reviewer #2: N/A

**********

4. Have the authors made all data underlying the findings in their manuscript fully available?

The PLOS Data policy

Reviewer #1: No

Reviewer #2: Yes

**********

5. Is the manuscript presented in an intelligible fashion and written in standard English?

Reviewer #1: Yes

Reviewer #2: Yes

**********

Reviewer #1: I thank the authors for their careful and constructive revision of the manuscript. The paper has improved substantially compared to the previous version, particularly in the restructuring of the Results section, which is now more readable and thematically organized.

At the same time, some issues remain that, in my opinion, should be addressed before the manuscript can be considered for publication.

- Minor Issues:

1. Level of detail in the Results section

While the effort to synthesize the Results section is appreciated and represents a clear improvement, the current version at times becomes too condensed, with a loss of informative detail.

In several instances, statements about participants’ misunderstandings remain qualitative and somewhat generic, without providing quantitative support. For example:

“[...] responses were frequently unsatisfactory, with many participants providing erroneous or illogical answers [...]”

“A majority of the participants endorsed several of these incorrect statements as true [...]”

Such formulations reduce interpretability and make it difficult for the reader to assess the magnitude of the reported effects.

In contrast, passages where proportions are reported (e.g., “Only 13.2% of participants correctly identified…”) are much more informative and should be preferred.

Suggestion: I recommend that the authors systematically include key quantitative indicators (e.g., percentages of correct/incorrect responses) in the main text when available, while still keeping extended details in the Supplementary Materials. This would improve both clarity and interpretability without substantially increasing length.

- Major Issues:

1. Pre-registration and transparency of the review protocol

The manuscript states that:

“A project plan was developed prior to initiating the study and is available at: https://osf.io/qzxut.”

However, the OSF record appears to have been created in May 27, 2025 and subsequently modified in September 1, 2025, whereas the manuscript reports that the literature search was conducted as early as December 2021 and updated in April 2023 and August 2025.

This raises a serious concern regarding the claim of a priori protocol development. As it stands, the review does not appear to have been pre-registered before data collection began.

This is not a minor issue. Pre-registration is a key component of methodological transparency in systematic reviews, as it allows readers and reviewers to assess the risk of selective reporting and analytic flexibility.

Required clarification:

The authors should explicitly clarify the timeline of protocol development.

If the review was not pre-registered prior to data collection, this must be clearly stated in the manuscript.

The current wording should be revised to avoid any implication of prospective registration if this was not the case.

Failure to clarify this point risks misleading the reader about the level of methodological rigor and transparency of the study.

2. Reproducibility of the search strategy

The manuscript indicates that detailed search strings are provided in “S1 Text”. However, this material is not accessible (neither in the manuscript nor in the Supplementary files made available for review).

Without the full search strings, the systematic review is not reproducible, and the “systematic” nature of the review cannot be independently evaluated.

This is a fundamental requirement for systematic reviews.

Required action:

The authors must provide the complete search strategies for all databases (including exact strings, limits, and filters used).

The referenced “S1 Text” should be included and clearly linked or uploaded as Supplementary Material.

Overall recommendation

The manuscript has improved and addresses an important and timely topic. However, the two major issues outlined above - particularly those related to transparency and reproducibility - are methodological and not merely editorial.

At this stage, I recommend a major revision

Reviewer #2: (No Response)

**********

what does this mean?). If published, this will include your full peer review and any attached files.

If you choose “no”, your identity will remain anonymous but your review may still be made public.

**Do you want your identity to be public for this peer review?**  For information about this choice, including consent withdrawal, please see our Privacy Policy

Reviewer #1: **Yes:**  Matteo Manente

Reviewer #2: **Yes:**  Margherita Calderan

**********

[NOTE: If reviewer comments were submitted as an attachment file, they will be attached to this email and accessible via the submission site. Please log into your account, locate the manuscript record, and check for the action link "View Attachments". If this link does not appear, there are no attachment files.]

To ensure your figures meet our technical requirements, please review our figure guidelines: https://journals.plos.org/plosone/s/figures

You may also use PLOS’s free figure tool, NAAS, to help you prepare publication quality figures: https://journals.plos.org/plosone/s/figures#loc-tools-for-figure-preparation.

NAAS will assess whether your figures meet our technical requirements by comparing each figure against our figure specifications.

# Author comment: Author response to Decision Letter 2

**DOI:** 10.1371/journal.pone.0362071.r004

7 Oct 2026

See attached Response to Reviewers file.

Attachment Submitted filename: Response_to_reviewers_auresp_2.docx

# Peer review: Decision Letter 2

**DOI:** 10.1371/journal.pone.0362071.r005

**Authors:** Elisa Scerrati

20 Oct 2026

Inferences of p-values, statistically significant results, and confidence intervals – a systematic review of empirical studies

PONE-D-26-01407R2

Dear Dr. Lytsy,

We’re pleased to inform you that your manuscript has been judged scientifically suitable for publication and will be formally accepted for publication once it meets all outstanding technical requirements.

Within one week, you’ll receive an e-mail detailing the required amendments. When these have been addressed, you’ll receive a formal acceptance letter and your manuscript will be scheduled for publication.

An invoice will be generated when your article is formally accepted. Please note, if your institution has a publishing partnership with PLOS and your article meets the relevant criteria, all or part of your publication costs will be covered. Please make sure your user information is up-to-date by logging into Editorial Manager at Editorial Manager® and clicking the ‘Update My Information' link at the top of the page. For questions related to billing, please contact billing support.

If your institution or institutions have a press office, please notify them about your upcoming paper to help maximize its impact. If they’ll be preparing press materials, please inform our press team as soon as possible -- no later than 48 hours after receiving the formal acceptance. Your manuscript will remain under strict press embargo until 2 pm Eastern Time on the date of publication. For more information, please contact onepress@plos.org.

Kind regards,

Elisa Scerrati

Academic Editor

PLOS One

Additional Editor Comments (optional):

Reviewers' comments:

Reviewer's Responses to Questions

**Comments to the Author**

Reviewer #1: All comments have been addressed

**********

2. Is the manuscript technically sound, and do the data support the conclusions?

Reviewer #1: Yes

**********

3. Has the statistical analysis been performed appropriately and rigorously?

Reviewer #1: N/A

**********

4. Have the authors made all data underlying the findings in their manuscript fully available?

The PLOS Data policy

Reviewer #1: Yes

**********

5. Is the manuscript presented in an intelligible fashion and written in standard English?

Reviewer #1: Yes

**********

Reviewer #1: I thank the authors for their careful revision and for addressing my previous comments.

I have reviewed the revised manuscript and I am satisfied with the changes. The additional quantitative information has improved the Results section, the issue of preregistration has been properly clarified, and the search strategies are now available in the Supplementary Materials.

Overall, I believe that my previous concerns have been adequately addressed and that the manuscript now meets the standards for publication in PLOS ONE. I have no further comments and recommend acceptance.

**********

what does this mean?). If published, this will include your full peer review and any attached files.

If you choose “no”, your identity will remain anonymous but your review may still be made public.

**Do you want your identity to be public for this peer review?**  For information about this choice, including consent withdrawal, please see our Privacy Policy

Reviewer #1: **Yes:**  Matteo Manente

**********

# Editor report: Acceptance letter

**DOI:** 10.1371/journal.pone.0362071.r006

**Authors:** Elisa Scerrati

PONE-D-26-01407R2

PLOS One

Dear Dr. Lytsy,

I'm pleased to inform you that your manuscript has been deemed suitable for publication in PLOS One. Congratulations! Your manuscript is now being handed over to our production team.

At this stage, our production department will prepare your paper for publication. This includes ensuring the following:

* All references, tables, and figures are properly cited

* All relevant supporting information is included in the manuscript submission,

* There are no issues that prevent the paper from being properly typeset

You will receive further instructions from the production team, including instructions on how to review your proof when it is ready. Please keep in mind that we are working through a large volume of accepted articles, so please give us a few days to review your paper and let you know the next and final steps.

Lastly, if your institution or institutions have a press office, please let them know about your upcoming paper now to help maximize its impact. If they'll be preparing press materials, please inform our press team within the next 48 hours. Your manuscript will remain under strict press embargo until 2 pm Eastern Time on the date of publication. For more information, please contact onepress@plos.org.

You will receive an invoice from PLOS for your publication fee after your manuscript has reached the completed accept phase. If you receive an email requesting payment before acceptance or for any other service, this may be a phishing scheme. Learn how to identify phishing emails and protect your accounts at https://explore.plos.org/phishing.

If we can help with anything else, please email us at customercare@plos.org.

Thank you for submitting your work to PLOS One and supporting open access.

Kind regards,

PLOS One Editorial Office Staff

on behalf of

Dr. Elisa Scerrati

Academic Editor

PLOS One
